# Lineage-Specific Profiling Delineates the Emergence and Progression of Naive Pluripotency in Mammalian Embryogenesis

**DOI:** 10.1016/j.devcel.2015.10.011

**Published:** 2015-11-09

**Authors:** Thorsten Boroviak, Remco Loos, Patrick Lombard, Junko Okahara, Rüdiger Behr, Erika Sasaki, Jennifer Nichols, Austin Smith, Paul Bertone

**Affiliations:** 1 Wellcome Trust–Medical Research Council Stem Cell Institute, University of Cambridge, Tennis Court Road, Cambridge CB2 1QR, UK; 2 European Bioinformatics Institute, European Molecular Biology Laboratory, Wellcome Trust Genome Campus, Cambridge CB10 1SD, UK; 3 Deutsches Primatenzentrum (German Primate Center), Leibniz-Institut für Primatenforschung, Kellnerweg 4, 37077 Göttingen, Germany; 4 DZHK (German Center for Cardiovascular Research), Wilhelmsplatz 1, 37073 Göttingen, Germany; 5 Department of Applied Developmental Biology, Central Institute for Experimental Animals, 3-25-12 Tonomachi, Kawasaki-ku, Kanagawa 210-0821, Japan; 6 Keio Advanced Research Center, Keio University, Shinjuku-ku, Tokyo 160-8582, Japan; 7 Department of Physiology, Development and Neuroscience, University of Cambridge, Tennis Court Road, Cambridge CB2 3EG, UK; 8 Department of Biochemistry, University of Cambridge, Tennis Court Road, Cambridge CB2 1GA, UK; 9 Genome Biology and Developmental Biology Units, European Molecular Biology Laboratory, Meyerhofstraße 1, 69117 Heidelberg, Germany

**Keywords:** pluripotency, inner cell mass, diapause, embryonic stem cell, primate

## Abstract

Naive pluripotency is manifest in the preimplantation mammalian embryo. Here we determine transcriptome dynamics of mouse development from the eight-cell stage to postimplantation using lineage-specific RNA sequencing. This method combines high sensitivity and reporter-based fate assignment to acquire the full spectrum of gene expression from discrete embryonic cell types. We define expression modules indicative of developmental state and temporal regulatory patterns marking the establishment and dissolution of naive pluripotency in vivo. Analysis of embryonic stem cells and diapaused embryos reveals near-complete conservation of the core transcriptional circuitry operative in the preimplantation epiblast. Comparison to inner cell masses of marmoset primate blastocysts identifies a similar complement of pluripotency factors but use of alternative signaling pathways. Embryo culture experiments further indicate that marmoset embryos utilize WNT signaling during early lineage segregation, unlike rodents. These findings support a conserved transcription factor foundation for naive pluripotency while revealing species-specific regulatory features of lineage segregation.

## Highlights

• Transcriptome profiling establishes a reference map for early mouse development
• Lineage-specific RNA-seq captures the full dynamic range of gene expression
• ESCs and diapaused and preimplantation epiblasts share common pluripotency circuitry
• Primate early ICM utilizes additional signaling pathways for lineage specification

## Teaser

In this Resource article, Boroviak et al. perform lineage-specific transcriptome analysis of early mouse, diapaused, and marmoset embryos, defining regulatory modules mediating the establishment and loss of naive pluripotency. Cross-species comparisons reveal a conserved pluripotency transcriptional network but distinct signaling pathways for segregation of the inner cell mass.

## Introduction

Pluripotency emerges in the mammalian epiblast during preimplantation blastocyst development. At the eight-cell stage, the embryo undergoes compaction and outer cells are directed toward the trophectoderm lineage. Interior cells become the inner cell mass (ICM) and subsequently diverge into pluripotent epiblast and extraembryonic primitive endoderm (PrE). In rodents, the preimplantation epiblast state can be captured in vitro as embryonic stem cells (ESCs) (Evans and Kaufman, 1981, Martin, 1981) and sustained indefinitely using defined media (Buehr et al., 2008, Li et al., 2008, Ying et al., 2008). The unrestricted potential of ESCs to generate all somatic tissues and the germline is termed “naive” pluripotency, and differs from other in vitro pluripotent states with respect to gene expression, signaling requirements, and epigenetic status (Brons et al., 2007, Leitch et al., 2013, Marks et al., 2012, Nichols and Smith, 2009, Tesar et al., 2007).

Individual epiblast cells rapidly lose the ability to give rise to self-renewing ESC colonies upon implantation (Boroviak et al., 2014), evidencing the transient nature of naive identity in normal development. The transition from the naive state is poorly characterized in vivo and in vitro. Upon implantation, epiblast cells acquire epithelial polarity (Bedzhov and Zernicka-Goetz, 2014) and lose naive pluripotency marker expression entirely by embryonic day (E)5.5 (Boroviak et al., 2014). However, the transcriptional network associated with this progression has been ill defined.

Pluripotency can be suspended in utero during diapause, a facultative condition of embryonic arrest (Mantalenakis and Ketchel, 1966, Mead, 1993, Renfree and Shaw, 2000). Embryonic diapause by delayed implantation has evolved to overcome conditions unfavorable for reproduction (Ketchel et al., 1966, Mantalenakis and Ketchel, 1966, Mead, 1993) and occurs in over 100 mammalian species (Renfree and Shaw, 2000). The distinction between transient, diapaused, and self-renewing pluripotent states remains elusive (Nichols and Smith, 2012).

Human and nonhuman primate pluripotent cells are maintained in vitro with culture regimes distinct from those used for mouse ESCs. They exhibit marked differences in gene expression compared to primate preimplantation embryos (Nakatsuji and Suemori, 2002, Thomson et al., 1996, Thomson et al., 1998, Yan et al., 2013). Propagation of nonhuman primate ESCs competent for chimera contribution has proven elusive (Kishi et al., 2014, Tachibana et al., 2012), although recent progress was reported for rhesus macaque using altered culture conditions (Fang et al., 2014). It is unclear why primate embryonic cells are more refractory to authentic ESC derivation compared to mouse. One contributing factor could be differences in signaling pathways driving lineage commitment and segregation in the ICM, which have not been defined in the primate.

Advances in sequencing protocols have enabled quantitative analysis of picogram amounts of RNA from individual cells and facilitated the study of early mammalian development at unprecedented resolution (Guo et al., 2010, Kurimoto et al., 2006, Tang et al., 2009). However, sequencing from minute quantities of starting material compromises the detection of low-abundance transcripts and impairs accurate estimation of expression levels (Brennecke et al., 2013, Grün et al., 2014). These limitations reduce the potential for comprehensive molecular characterization of individual cells. Here we show that this can be mitigated by applying single-cell sample preparation methods to small groups of cells. We adapted single-cell reverse-transcription and preamplification (Tang et al., 2009) for compatibility with Illumina sequencing and profiled clusters of 8–20 cells. This approach yields substantial enhancement in transcript detection and is suitable for experiments that call for accurate analysis of small cell numbers without a requirement to measure cell-to-cell variability.

We apply this technique to produce a transcriptional map of early mouse development from morula to postimplantation epiblast, and compare in vitro cultured ESCs and diapaused epiblasts in the context of this developmental sequence. We extend this analysis to the common marmoset (*Callithrix jacchus*), a small New World monkey presenting several practical advantages as a model for primate embryology. Profiling transcriptional activity in the inner cell mass of early-, mid-, and late-stage marmoset blastocysts allows direct comparison of rodent and primate development. These data reveal differences in regulatory timing and utilization of signaling pathways that we investigate through embryo culture experiments. Our results provide a framework for delineating the emergence and developmental progression of pluripotency in diverse mammals.

## Results

### A Transcriptional Map of Early Mouse Development

**Figure 1:**
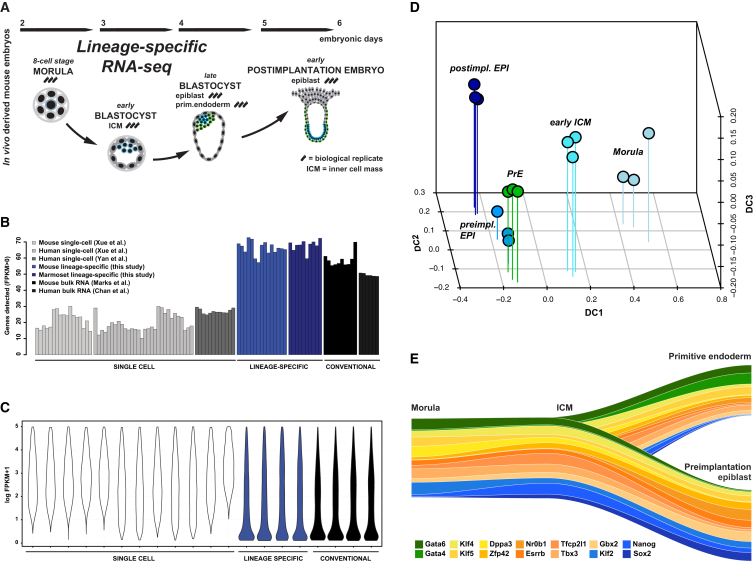
Transcriptome Profiling of Mouse Embryonic Lineages (A) Overview of the developmental sequence analyzed. (B) Percentage of detected genes in RNA-seq data from single cells (white), small numbers of cells (blue), and conventional bulk RNA (black) on comparable cell types (Xue et al., 2013, Yan et al., 2013, Marks et al., 2012). (C) Distribution of nonzero expression values in log_2_ FPKM (fragments per kilobase of exon per million fragments mapped) for RNA-seq data from single cells (white), small numbers of cells (blue), and conventional bulk RNA (black). (D) Diffusion map of embryonic samples from morula to postimplantation epiblast; DC, diffusion coefficient. (E) Marker expression delineates the divergence of epiblast and PrE lineages. Genes specific to PrE and the preimplantation epiblast are marked in green and blue, respectively; shared genes are depicted in orange. Track width is scaled to relative expression normalized to the mean across all stages displayed.

We previously showed by qRT-PCR that a moderate increase in starting material for whole-transcriptome amplification (Tang et al., 2009) substantially increases signal fidelity and reproducibility (Boroviak et al., 2014). We extend this approach to RNA sequencing (RNA-seq) to profile cell lineages from individual mouse embryos. Four time points were analyzed from the eight-cell stage at E2.5 to the early postimplantation epiblast at E5.5 (Figure 1A; Table S1). *Pdgfra::GFP* knockin mice (Hamilton et al., 2003, Plusa et al., 2008) enabled fluorescence-based separation of PrE from epiblast cells in E4.5 and E5.5 blastocysts.

We assessed transcript detection and expression-level estimation relative to previously published single-cell (Xue et al., 2013, Yan et al., 2013) and conventional RNA-seq data (Chan et al., 2013, Marks et al., 2012). Transcription was measured from up to 30% of annotated genes by single-cell RNA-seq, consistent with previous reports (Brennecke et al., 2013, Grün et al., 2014). RNA-seq from 10–20 cells (8 in the case of E2.5 morulae) yielded detection rates of 60%–70%, comparable to the performance of sequencing protocols from microgram quantities of RNA (Figure 1B). Similar distribution profiles were observed from bulk RNA and small numbers of cells, with many genes expressed at low and intermediate levels and a small proportion showing high expression (Figure 1C). In contrast, single-cell data exhibit high expression-level estimates for many genes and missing values for low-abundance transcripts (Kharchenko et al., 2014). These results demonstrate that profiling small cell clusters overcomes limitations in sensitivity of single-cell analysis and allows quantification of gene expression levels comparable to that of conventional transcriptome sequencing.

Analysis of biological replicates spanning the five embryonic stages produced discrete clusters, recapitulating their developmental sequence (Figure S1A). Visualization by diffusion map, a nonlinear dimensionality reduction method (Lafon et al., 2006), shows that samples cluster primarily by stage, with the first coefficient capturing progression of development (Figure 1D). PrE and preimplantation epiblast cells retain a high degree of similarity despite the divergent developmental potential of the two lineages (Figure 1D; Figure S1A).

We examined pluripotency and lineage markers in more detail. Our data confirm the sequential activation of PrE specifiers previously described (Figure S1B) (Artus et al., 2011, Chazaud et al., 2006, Gerbe et al., 2008, Guo et al., 2010, Kurimoto et al., 2006, Ohnishi et al., 2014), whereas preimplantation epiblast cells exhibited robust expression of exclusive markers including *Sox2*, *Nanog*, *Klf2*, *Bmp4*, and *Fgf4* (Guo et al., 2010, Kurimoto et al., 2006, Tang et al., 2010a). Strikingly, however, several transcription factors associated with pluripotency were expressed in both lineages. *Pou5f1* (*Oct4*) was expressed at lower levels but was still present in PrE, consistent with known protein distribution (Frum et al., 2013, Le Bin et al., 2014, Palmieri et al., 1994). This pattern was evident for *Klf4*, *Dppa3*, *Nr0b1*, *Esrrb*, and *Zfp42* (*Rex1*). *Klf5* and *Tbx3* were expressed at higher levels in PrE (Figure 1E). This observation is reflected in the cluster proximity of PrE and the preimplantation epiblast (Figure 1D). These results illustrate that divergent potency of related embryonic lineages can be conferred by a small subset of regulatory factors in the context of a globally similar transcriptome.

### Defining Stage-Specific Gene Expression Modules in Early Mouse Development

**Figure 2:**
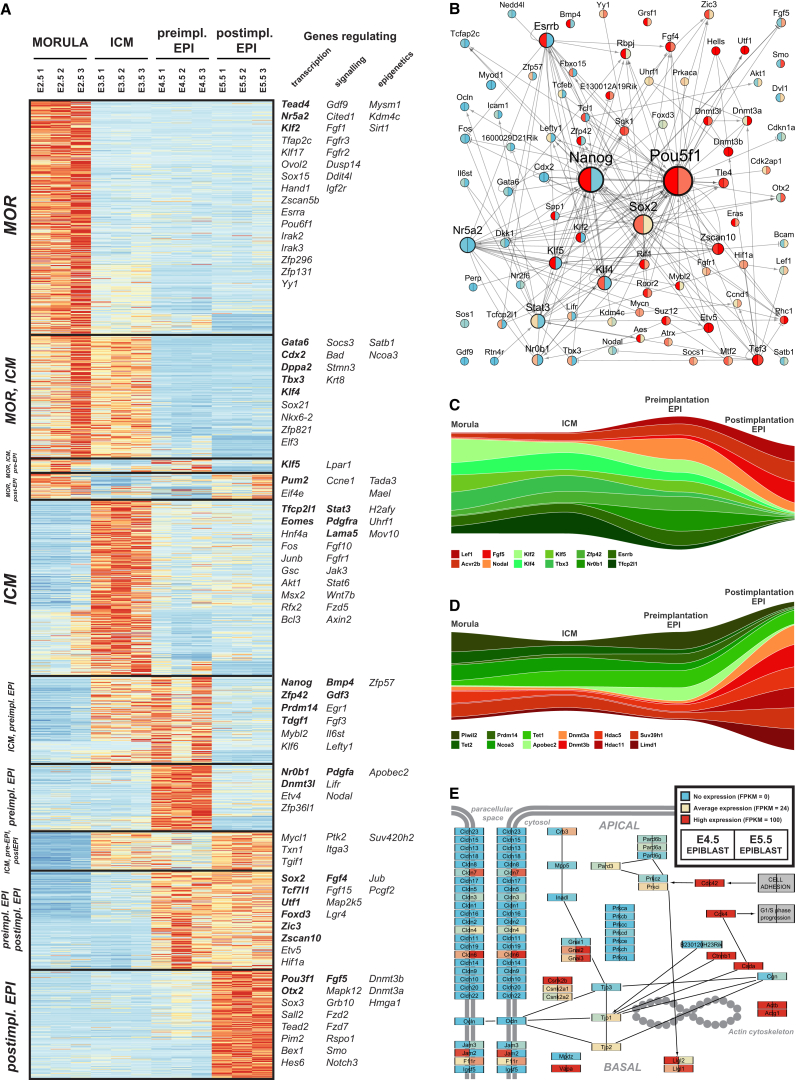
Expression Modules Identified in Early Mouse Development and the Preimplantation-to-Postimplantation Epiblast Transition (A) Expression of dynamically expressed genes. Modules were derived by hierarchical clustering of scaled expression values. Selected transcription factors, signaling pathway components, and epigenetic regulators are shown with pluripotency-associated genes in bold. (B) PluriNet82 at the transition from pre- to postimplantation stages. Label and node sizes reflect interaction number. Colors represent expression in FPKM for preimplantation (E4.5; left) and postimplantation epiblasts (E5.5; right). Arrows indicate positive interactions; T bars indicate inhibitions. (C) Genes characteristic of preimplantation (green) and early postimplantation development (red). Track width is scaled to relative expression normalized to the mean across all stages displayed. (D) Epigenetic modifiers expressed predominantly at preimplantation (green) or early postimplantation (red) stages. (E) Simplified representation of the KEGG “tight junction” pathway, with nodes colored according to expression in the preimplantation (left) and postimplantation epiblast (right).

Lineage-specific profiling facilitated identification of gene sets indicative of developmental state, lineage segregation, and progression. We defined dynamically expressed genes as those transcribed differentially between at least two of the four embryonic stages analyzed and robustly detected in at least one (fragments per kilobase of exon per million fragments mapped [FPKM] > 10). The resultant set comprises 1,857 genes (Table S2), and hierarchical clustering revealed ten expression modules (Figure 2A; Figure S2A).

Genes maximally expressed in morulae included *Tead4*, *Sox15*, *Klf17*, *Nr5a2*, and *Tfap2c*. Those common to morulae and the ICM were *Gata6*, as well as trophectodermal markers *Hand1*, *Elf3*, and *Eomes*. The latter group may underlie the capacity of early ICM cells to regenerate trophectoderm (Handyside, 1978, Nichols and Gardner, 1984, Rossant and Lis, 1979). Genes expressed throughout preimplantation development but at varying levels included ESC markers *Klf2*, *Klf4*, *Klf5*, *Nr0b1*, *Zfp42*, *Prdm14*, *Tbx3*, *Tfcp2l1*, and *Esrrb*. These data provide a comprehensive set of transcription factors and other genes downregulated upon implantation (Table S2).

In addition to *Pou5f1* (*Oct4*) and *Sall4*, pluripotency markers associated with both pre- and postimplantation epiblast include *Utf1*, *Foxd3*, *Zic3*, and *Fgf4*. *Tcf7l1* (*Tcf3*), a gene expressed in embryonic stem cells and involved in pluripotency repression (Martello et al., 2012, Wray et al., 2011, Yi et al., 2011), was upregulated at E4.5, potentially to prepare naive epiblast cells for transition. Notably, 220 genes were specifically upregulated during the preimplantation-to-postimplantation epiblast transition. These include known regulators of early postimplantation development, such as *Pou3f1* (*Oct6*), *Fgf5*, *Otx2*, and *Sox3*, as well as candidate regulators *Tead2*, *Sall2*, *Mapk12*, *Fzd2*, *Rspo1*, *Smo*, and *Notch3*. We also observed strong upregulation of de novo methyltransferases *Dnmt3a* and *Dnmt3b*, consistent with hypomethylation in naive pluripotent cells (Leitch et al., 2013, Smith et al., 2012) and increased DNA methylation upon implantation (Lee et al., 2014).

Genes expressed at high levels throughout the developmental stages analyzed are listed in Table S3, and expression modules specific to the epiblast versus PrE segregation process are provided in Figure S2B and Table S4. These results expand the recent description of PrE-associated genes by single-cell microarray profiling (Ohnishi et al., 2014) and provide a resource to identify regulators of PrE differentiation.

The initial progression from naive pluripotency in embryonic development has not been well characterized. We analyzed the E4.5-to-E5.5 transition before epiblast cells undergo lineage priming in vivo and provide the complete set of differentially expressed genes in Table S5. To examine the behavior of an independent set of pluripotency-associated genes, we used PluriNetWork, curated from published interaction data (Som et al., 2010). Focusing on dynamically expressed genes and the core regulators *Pou5f1*, *Nanog*, and *Sox2*, we reduced the network to 82 nodes (PluriNet82; Figure 2B). Importantly, the condensed network includes all validated regulators of ESC pluripotency. Those downregulated during the E4.5-to-E5.5 transition include naive markers Esrrb, Nr0b1, Klf2, Klf4, Klf5 and Zfp42 (Rex1), as well as Lifr, Il6st (gp130), Spp1, Tcl1, and Zfp57. Conversely, we observed upregulation of *Foxd3*, *Lef1*, *Ccnd1*, *Zscan10*, *Phc1*, and *Nr216* upon implantation.

Visualization of temporal patterns shows robust expression of naive markers during preimplantation development followed by an abrupt shutdown and replacement with factors such as *Nodal*, *Lef1*, and *Fgf5*, along with the activin receptor *Acvr2b* (Figure 2C). Epigenetic modifiers associated with a permissive chromatin state (*Tet1*, *Tet2*, *Prdm14*, *Ncoa3*) were predominant during preimplantation development (Figure 2D). Upon implantation, these were exchanged for epigenetic regulators mediating DNA methylation (*Dnmt1*, *Dnmt3a*, *Dnm3b*) and transition to more closed chromatin configurations (*Hdac5*, *Hdac11*, *Suv39h1*).

We integrated the RNA-seq data with 229 annotated Kyoto Encyclopedia of Genes and Genomes (KEGG) pathways and generated expression maps for pairwise comparison of developmental stages (http://pathway-atlas.stemcells.cam.ac.uk). At the preimplantation-to-postimplantation epiblast transition, we noted differential expression of genes involved in tight junction formation (Figure 1E). In particular, we detected upregulation of *Cldn6*, important for epithelial formation (Turksen and Troy, 2001) and upregulated in mouse epiblast-derived stem cells (Tesar et al., 2007), and *Cldn7* with *Tjp1*, essential for tight junction establishment (Matter and Balda, 1999, Sleeman and Thiery, 2011). We also noted induction of *Crb3* and *Pard3*, pivotal for the establishment and maintenance of apical-basal polarity (Shin et al., 2006). These results comprise a reference dataset for regulatory network and pathway analysis during epiblast progression in vivo.

### The Transcriptional Circuitry for ESC Identity Is Assembled in the Preimplantation Epiblast

**Figure 3:**
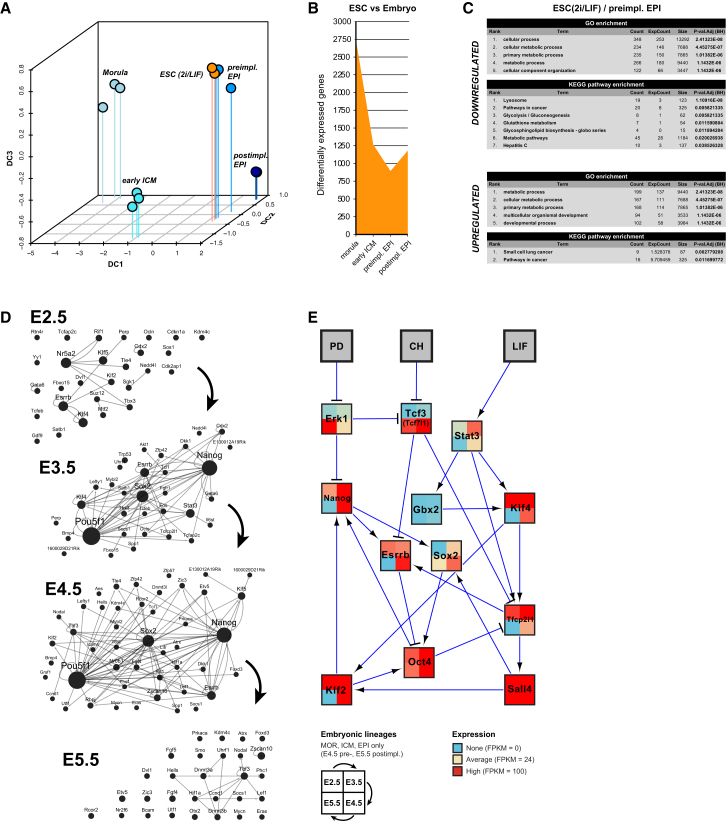
ESCs Retain Expression Modules Defining the Preimplantation Epiblast (A) Diffusion map from morula to postimplantation epiblast and ESC cultured in 2i/LIF, based on dynamically expressed genes. (B) Genes differentially expressed (p < 0.05) between ESCs and embryonic samples. (C) Most significantly enriched GO and KEGG pathways based on up- and downregulated genes in ESC versus preimplantation epiblast. (D) Changes in expression of PluriNet82 genes in the embryonic lineage. A node is displayed if the corresponding gene is predominantly active at that developmental stage, defined as positive log-transformed expression relative to the mean across all stages. An edge is displayed if both source and target nodes are active. (E) Minimal set of transcription factors operative in mouse ESCs (Dunn et al., 2014). Colors represent gene expression in FPKM. Expression levels are depicted for morula, early ICM, and pre- and postimplantation epiblasts clockwise from the top left.

We sequenced RNA from ESCs cultured in 2i/LIF (leukemia inhibitory factor) without feeders or serum (Ying et al., 2008), processed identically to embryonic samples to allow direct comparison. Global analysis showed tight correlation between biological replicates and confirmed the shared identity of ESCs and the preimplantation epiblast (Figures S3A and S3B). Restricting the comparison to dynamically expressed genes reveals concordance in the developmental state, with ESCs displaying a near-identical profile to the E4.5 epiblast (Figure 3A; Figure S3C). Consistent with this, genes differentially expressed between ESCs and embryonic cells were fewest for the E4.5 epiblast, indicating the greatest correspondence with emergent pluripotent cells in the blastocyst (Figure 3B). Pathway enrichment analyses showed most changes in ESCs to be associated with metabolism, potentially rooted in the biophysical environment and nutrient utilization (Figure 3C).

We examined the behavior of regulatory networks governing pluripotency in ESCs in the context of in vivo development. To capture changes over time, we computed normalized expression relative to the mean level of each gene across all developmental stages and defined genes with positive values as preferentially active at a given stage. Mapping these values to PluriNet82 reveals changes in network topology during early mouse development (Figure 3D). Few pluripotency genes exhibited robust expression at the morula stage, with substantially more upregulated in the early ICM. Most peaked at the preimplantation epiblast stage, resulting in maximum interconnectivity. This was abolished following implantation, marking the dissolution of naive pluripotency in vivo.

We proposed a reduced set of abstract Boolean network models for ESC self-renewal comprising 11 transcription factors and the extracellular signal-regulated kinase (Erk)/mitogen-activated protein (MAP) kinase (Dunn et al., 2014). To relate this to the embryo, we asked whether a developmental stage can be identified at which all components are present. Intersecting data from morula, early ICM, and pre- and postimplantation epiblast cells revealed that the only time point at which these regulators were coexpressed was the preimplantation epiblast (Figure 3E). Thus, transcriptional regulation in cultured ESCs correlates specifically with the naive phase of pluripotency in the embryo.

### Diapaused Epiblasts Maintain All Features of Naive Pluripotency

**Figure 4:**
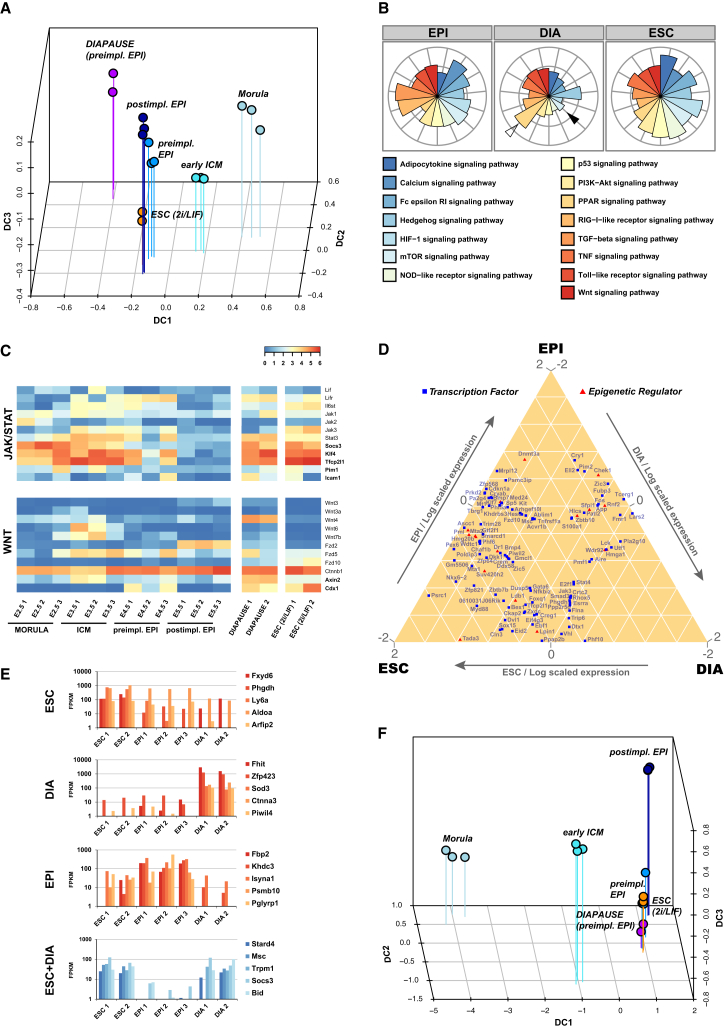
Relationship between Diapaused Epiblast, Normal Embryonic Development, and ESC (A) Diffusion map of developmental stages from morula to postimplantation epiblast, ESC, and diapaused epiblast. (B) Expression scores for selected signaling pathways, scaled to the mean across the three cell types, calculated by summing FPKM values of individual components followed by normalization for pathway size. (C) Selected components of WNT and JAK/STAT signaling pathways for the samples indicated. (D) Ternary plot of the most divergent transcriptional and epigenetic regulators between preimplantation epiblast, ESC, and diapaused epiblast. FPKM values are scaled to the mean across the three cell types and are log transformed. (E) Differentially expressed genes in FPKM between preimplantation epiblast, diapaused epiblast, and ESC, as indicated. (F) Diffusion map based on dynamically expressed genes from morula to postimplantation epiblast, ESC, and diapaused epiblast.

Rodents have evolved the capacity for facultative developmental arrest at the late blastocyst stage (Mantalenakis and Ketchel, 1966, Mead, 1993, Renfree and Shaw, 2000). ESCs were first obtained from embryos in diapause (Evans and Kaufman, 1981), and the condition is known to facilitate ESC derivation (Brook and Gardner, 1997). We induced implantation delay by ovariectomy (Weitlauf and Greenwald, 1968) and isolated diapaused epiblasts for RNA-seq as above. Diapaused embryos vary from those undergoing normal development (Figure 4A) and cluster separately by correlation analysis (Figure S4A). To characterize these differences, we examined Gene Ontology (GO) term enrichment for differentially expressed genes (Figure S4B). In diapause, the most significant downregulated processes relate to metabolism, cell cycle, and biosynthesis. Conversely, those upregulated include negative regulation of metabolism and biosynthetic processes. Apart from peroxisome proliferator-activated receptor (PPAR) signaling, pathway expression scores were generally reduced in diapaused epiblasts and particularly components of the mechanistic target of rapamycin (mTOR) pathway (Figure 4B). These features likely reflect a general state of dormancy in diapaused embryos.

We found all pluripotency factors to be expressed in the diapaused epiblast, indicating retention of naive identity (Figure S4C). It has been shown that *Lifr* and *Il6st* serve an essential function in epiblast maintenance during diapause (Nichols et al., 2001), implicating the Janus kinase (JAK)/signal transducer and activator of transcription (STAT) pathway in sustaining pluripotency. Analysis of JAK/STAT components revealed that receptors, signal transducers, and downstream targets are robustly expressed in normal preimplantation development, ESCs, and diapaused epiblasts but are not maintained postimplantation (Figure 4C). Pluripotency factor targets of Stat3, Klf4, and Tfcp2l1 (Bourillot et al., 2009, Hall et al., 2009, Martello et al., 2013, Niwa et al., 2009, Ye et al., 2013) were expressed at high levels in diapause. We also observed substantial upregulation of *Wnt4*, concomitant with high expression of the WNT targets *Esrrb* (Martello et al., 2012), *Axin2* (Lustig et al., 2002), and *Cdx1* (Pilon et al., 2007) (Figure 4C; Figure S4C). *Wnt* expression in normal development was mainly confined to the early ICM (*Wnt6*, *Wnt7b*), with low levels of *Wnt4* present in all stages analyzed. Thus, WNT signaling may play a specific role in extended maintenance of the pluripotency network during diapause.

We assessed genome-wide differences in expression of transcription factors and epigenetic regulators between the preimplantation epiblast, diapause, and ESC (Figure 4D). Few factors were highly expressed in only one condition and most were present in at least two, in particular ESC and preimplantation epiblast or ESC and diapause. Expression modules including all genes for preimplantation epiblast, diapause, and ESC appear in Table S4. ESC-specific transcripts include *Phgdh* and *Aldoa*, involved in glycolysis and L-serine biosynthesis, respectively (Figure 4E). *Piwil4*, a repressor of transposable elements, and *Sod3*, a superoxide dismutase, may act to maintain genomic integrity during diapause. Genes highly upregulated in both diapause and ESC included *Socs3*, a JAK/STAT downstream target, and *Stard4*, encoding a putative lipid transfer protein. We examined the relationship of diapaused epiblasts to embryo samples and ESCs based on all 1,857 genes dynamically expressed during normal development. Remarkably, diapause samples were placed directly adjacent to the preimplantation epiblast and ESC in the diffusion map (Figure 4F). These results show self-renewing ESCs and diapaused embryos to embody an arrested state of the transient E4.5 epiblast that retains developmental identity, despite substantial changes in environment in the case of ESCs and profound changes in metabolism, proliferation, and biosynthetic activity in diapause.

### Direct Comparison of Rodent and Primate ICM

Preimplantation embryogenesis in primates is protracted relative to rodents, and detailed molecular and functional characterizations are lacking. Protocols for minimally invasive embryo recovery have been developed for marmoset, providing access to embryos that have undergone normal gestation in utero (Thomson et al., 1994, Hanazawa et al., 2012), a resource that is not accessible from humans. We therefore utilized the common marmoset to investigate the transcriptional makeup of primate ICM in comparison to early mouse development.

**Figure 5:**
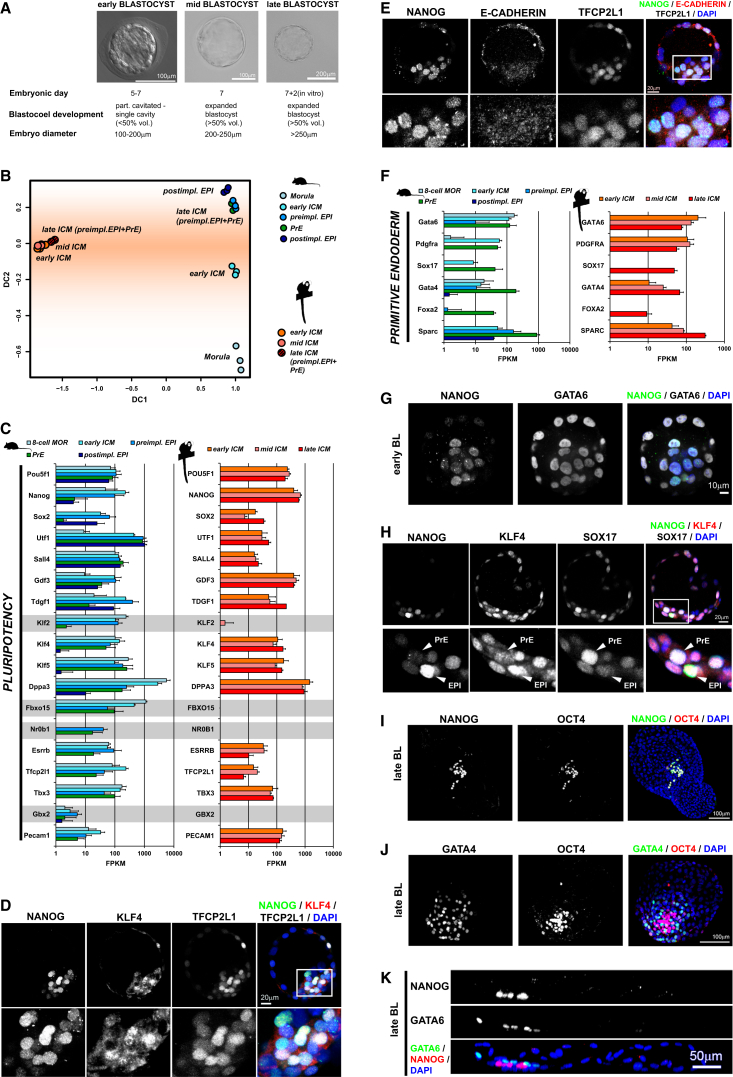
Pluripotency Factors Are Conserved, whereas Signaling Receptors Diverge, in Mouse and Marmoset ICM (A) Staging criteria for isolation of early, mid, and late marmoset ICM and images of the blastocysts analyzed. (B) Diffusion map of mouse and marmoset embryonic samples. (C) Pluripotency gene expression for mouse and marmoset embryonic samples. Error bars represent SD. (D and E) Confocal z projections of whole-mount marmoset early-mid blastocyst immunofluorescence stainings for the markers indicated. (F) PrE-associated gene expression for mouse and marmoset embryonic samples. Error bars represent SD. (G–J) Immunofluorescence stainings of early, mid (H), and late marmoset blastocyst. (K) z-x cross-section of marmoset blastocyst stained for the markers indicated.

Marmoset embryos were collected by nonsurgical uterine flush. The ICMs of early-, mid-, and late-stage blastocysts were isolated by immunosurgery (Figures S5A–S5C) and profiled by RNA-seq (Table S6). Marmoset ICMs were processed whole due to lack of reporter systems in the primate. Mixed expression of epiblast and PrE markers is therefore expected from late ICM (Figures 5C and 5D). Samples clustered largely by developmental stage (Figure S5H). Substantial differences between species were apparent (Figure 5B), embodied primarily in the first diffusion coefficient. The second coefficient appears to reflect progression of development in both mammals.

Expression of many pluripotency factors, including *Pou5f1*, *Sox2*, *Nanog*, *Esrrb*, *Klf4*, *Tbx3*, and *Tdgf1*, was conserved between species (Figure 5C). However, we noted changes in PluriNet82 connectivity imparted by the absence of *KLF2*, *FBXO15*, *NR0B1*, and *GBX2* in marmoset (Figure 2B). Two of these, *Klf2* and *Gbx2*, are proposed core naive pluripotency regulators in mouse ESCs (Figure 3E). We then assessed localization of naive pluripotency factors by immunofluorescence at the early-mid blastocyst stage. KLF4 and TFCP2L1 were predominantly expressed in the ICM and largely correlated with NANOG, albeit with slightly broader distribution (Figures 5D and 5E). These factors are thus coexpressed in a subset of ICM cells in the marmoset blastocyst, as also observed in human (Takashima et al., 2014). Intriguingly, E-CADHERIN staining was intense in the trophectoderm but diffuse in the ICM (Figure 5E).

Distinctions evident from the wider pluripotency network prompted us to investigate potential differences in epiblast and PrE specification in primate ICM. In mouse, sequential activation of early PrE markers (*Gata6*, *Pdgfra*) is followed by upregulation of late markers (*Gata4*, *Foxa2*) upon lineage segregation (Figure 5F). The majority of key PrE regulators, including *GATA6*, *SOX17*, and *GATA4*, were also present at high abundance in marmoset ICM. We performed further immunostaining for *GATA4* and *GATA6* to assess specificity to PrE. Initially, NANOG and GATA6 were coexpressed in the early ICM (Figure 5G), similar to mouse (Plusa et al., 2008, Schrode et al., 2014) and human (Roode et al., 2012). At the mid-blastocyst stage, we observed mutually exclusive staining of NANOG and SOX17 (Figure 5H). KLF4 was expressed at low levels throughout the embryo, but with stronger signal in NANOG-positive cells of the ICM (Figure 5H). OCT4 tightly colocalized with NANOG in the presumptive epiblast of late blastocysts (Figure 5I). In contrast, GATA4 and OCT4 staining were mutually exclusive in the ICM, with GATA4 specific to PrE and OCT4 confined to the epiblast (Figure 5J). Confocal microscopy of late marmoset blastocysts revealed that GATA6-positive cells formed a distinct layer overlying NANOG-positive cells within the ICM, indicating restriction of GATA6 to PrE (Figure 5K). We conclude that mouse and marmoset share the majority of PrE specifiers.

### FGF and WNT Signaling Are Required for Lineage Segregation in the Marmoset Blastocyst

To further relate mouse and marmoset embryos, we combined data from E4.5 epiblast and PrE mouse samples to allow comparison to the late marmoset ICM (Figure S5J; Table S7). Hierarchical clustering of an independent panel of genes selected by the International Stem Cell Initiative (Adewumi et al., 2007) revealed broad conservation in expression patterns of many pluripotency and lineage markers but differences in signaling pathway components, including *Lifr*, *Fgf4*, and *Nodal* (Figure S5J). Knowledge of pathways regulating PrE segregation and epiblast specification in primates is limited. We focused on the regulation of pathway components at the early ICM stage, when *NANOG* and *GATA6* are coexpressed (Figure 5G) and individual cells are indistinguishable at the transcriptome level in mouse (Ohnishi et al., 2014). The most significant differentially expressed pathways in marmoset included ascorbate and aldorate, inositol phosphate and lipoic acid metabolism, lysine biosynthesis, and PPAR signaling (Figure S5I). Pathways upregulated in mouse included arginine and proline metabolism and proteasome, estrogen receptor, and mTOR signaling. These results highlight metabolic differences, in particular with regard to amino acid biosynthesis, between the mouse and marmoset embryo.

**Figure 6:**
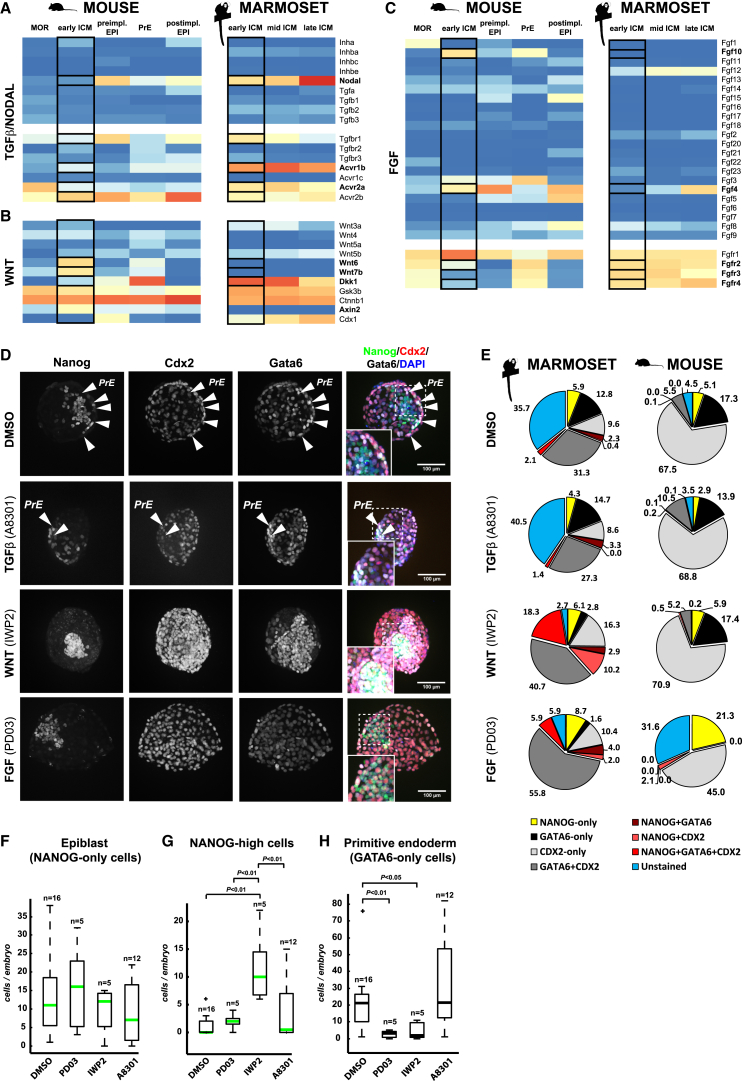
FGF and WNT Inhibition Disrupt Lineage Segregation in the Marmoset Blastocyst (A–C) Expression of selected components of the TGF-β/NODAL, FGF, and WNT signaling pathways. (D) Confocal z projections of inhibitor-treated marmoset late blastocysts stained for NANOG, CDX2, GATA6, and DAPI. (E) Fluorescence signal from cells labeled for lineage markers in mouse and marmoset embryos. Morulae were cultured under identical conditions to the late blastocyst stage in the presence of A8301 (3 μM), PD0325901 (3 μΜ), IWP2 (3 μΜ), or DMSO (control) for 3 and 4 days in mouse and marmoset, respectively. (F–H) Quantification of (F) NANOG-only, (G) NANOG-high, and (H) GATA6-only cells. Plotted are the first and second quartiles of data points (boxed) with error bars at minimum and maximum values. Outliers are indicated with a cross. NANOG-high cells displayed at least 1.5× average NANOG fluorescence intensity. p values were computed by one-way ANOVA with Tukey HSD (honest significant difference) testing.

We then examined signaling pathways by combining gene expression with KEGG pathway maps. We observed pronounced differences in major cascades such as transforming growth factor β (TGF-β), fibroblast growth factor (FGF), and WNT (Figures 6A–6C; Figure S6). Marmoset early ICM cells expressed high levels of *ACVR1B* (*ALK4*), *TGFBR1* (*ALK5*), and *ACVR2A* (Figure 6A; Figure S6A). Interestingly, *BMP4* is not detected in marmoset (Figure S6D), but *NODAL* is expressed from the early blastocyst stage (Figure 6A; Figure S6A). *FGF4* is absent in the early ICM and is upregulated at later stages. Additionally, we noted altered levels of FGF receptor expression (Figure 6C; Figure S6C). In relation to WNT signaling, the secreted Wnt inhibitor *DKK1* and the negative regulator of β-catenin *GSK3β* were highly upregulated, which together with reduced β-catenin (*CTNNB1*) (Figure 6B; Figure S6B) may indicate suppression of WNT signaling in the early primate ICM. At the late blastocyst stage, however, *DKK1* diminished, concomitant with an increase in *CDX1*. This suggests the possibility of a specific role for the WNT pathway in the marmoset blastocyst. Thus, although transcription factor expression is largely conserved between the rodent and primate embryo stages examined, the repertoire of signaling pathway components differs.

To investigate the functional relevance of these expression patterns, marmoset morulae were cultured to the late blastocyst stage in the presence of selective pathway inhibitors (Figure 6D). Embryos were subsequently immunostained for NANOG, GATA6, and CDX2. Cell number and fluorescence intensities were measured with automated analysis software (Volocity; PerkinElmer) (Figure 6E, left column; Figures S7A–S7F). Mouse embryos were cultured and analyzed under identical conditions for direct comparison. Inhibitor treatment did not affect total cell number (Figure S7G). In marmoset DMSO-treated controls, the epiblast and PrE had segregated in late blastocysts, as indicated by mutually exclusive NANOG (NANOG-only) and GATA6 (GATA6-only) staining in subsets of inner cells (Figure 6D, arrowheads). GATA6 was also expressed in the trophectoderm, but at lower levels and in combination with CDX2 (Figures S7G and S7H). The ratio of epiblast (NANOG-only) to PrE (GATA6-only) cells was higher in marmoset embryos compared to mouse (Figure 6E).

In mouse embryos, inhibition of FGF signaling ablated PrE formation and increased the epiblast compartment (Figure 6E), whereas inhibition of WNT and TGF-β/Nodal signaling did not elicit significant effects, consistent with previous reports (Biechele et al., 2013, Nichols et al., 2009, Yamanaka et al., 2010). In marmoset, blocking the type I TGF-β/activin/Nodal receptor with A8301 did not disrupt lineage segregation (arrowheads for GATA6-only cells in Figure 6D). However, inhibition of ERK or WNT signaling led to coexpression patterns rarely observed in control embryos (Figure 6E; Figure S7H). NANOG-only cells in marmoset embryos increased upon ERK pathway inhibition (Figures 6D–6F). WNT signaling inhibition led to strong upregulation of NANOG, GATA6, and CDX2, and in particular the number of NANOG-high cells was substantially greater (Figures 6D, 6E, and 6G). Strikingly, emergence of GATA6-only PrE cells was suppressed by both ERK and WNT signaling inhibition (Figure 6H; Figure S7I).

**Figure 7:**
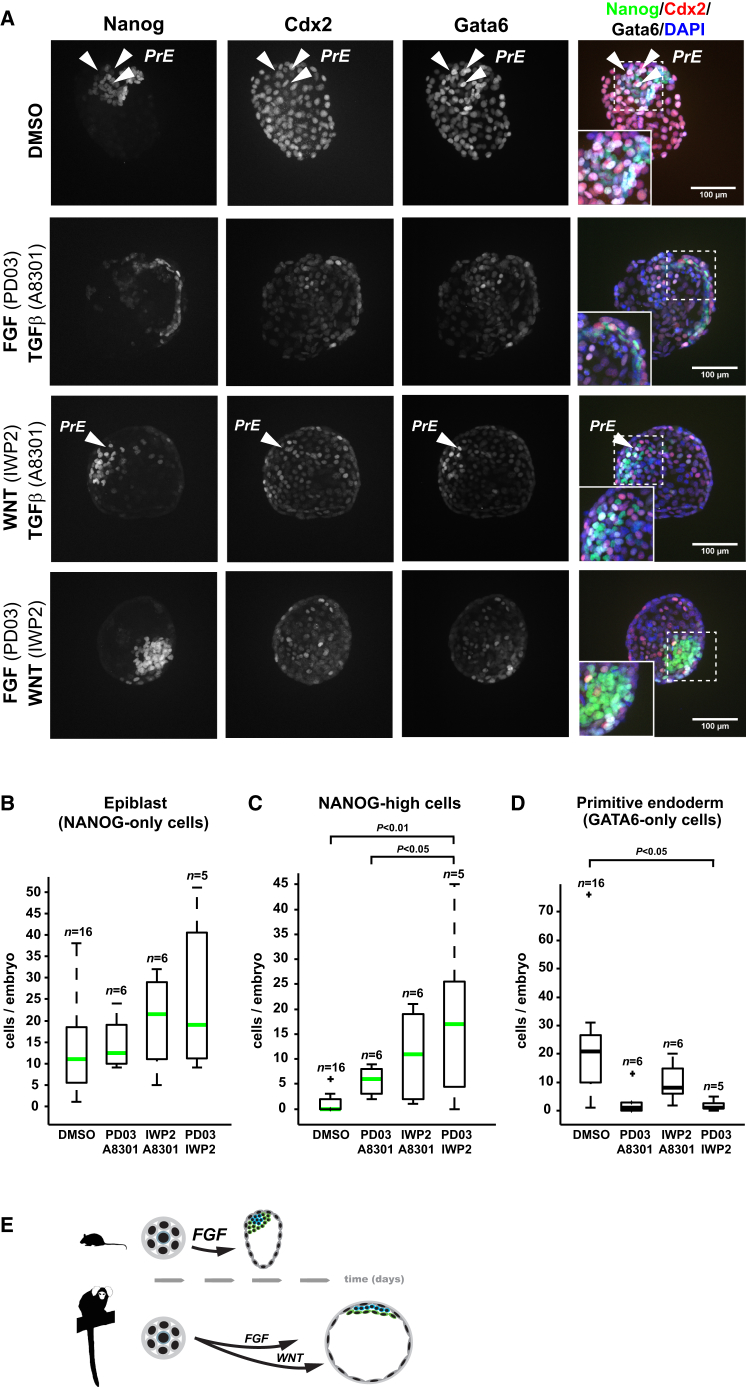
Dual FGF and WNT Inhibition Increases NANOG Levels and Blocks PrE Formation (A) Confocal z projections of inhibitor-treated marmoset late blastocysts stained for NANOG, CDX2, GATA6, and DAPI. (B–D) Fluorescence quantification of (B) NANOG-only, (C) NANOG-high, and (D) GATA6-only cells. Outliers are indicated with a cross. NANOG-high cells displayed at least 1.5× average NANOG fluorescence intensity. p values were computed by one-way ANOVA with Tukey HSD testing. (E) Model of pathways driving lineage specification in mouse and marmoset ICM.

We assessed whether the observed effects of TGF-β, WNT, and ERK inhibition might be combined by carrying out dual-inhibition experiments (Figure 7A). Conditions including WNT inhibition led to greater proportions of NANOG-only cells (Figure 7B), with a substantial increase in NANOG-high cells (Figure 7C). PrE formation was impaired in all dual-inhibition experiments (Figures 7A and 7D). The most profound reduction occurred when both WNT and ERK signaling were inhibited (Figure 7D), indicating an additive effect. These results support a conserved role for FGF/ERK signaling and a marmoset-specific function for WNT signaling in ICM lineage segregation (Figure 7E).

## Discussion

In this study, we applied single-cell RNA-seq protocols to clusters of cells to determine genome-wide transcriptional activity in developing embryonic lineages. This analysis defined stage-specific gene expression modules of lineage identity and fate specification. Furthermore, our data establish that mouse ESCs and diapaused epiblasts sustain preimplantation epiblast identity despite major environmental or metabolic changes. We found the regulatory network governing naive pluripotency in ESCs to be progressively activated in the embryo and maximally around E4.5. Heterogeneous ICM cells may individually progress to this stage over the preceding 12–18 hr. Indeed, from E3.75 onward, ESCs can be derived from single ICM cells in stringent 2i/LIF culture (Boroviak et al., 2014).

The gene expression module of the pre- and postimplantation epiblast contains drivers that act to dismantle the naive pluripotency circuitry. Prominent examples include *Fgf4*, an activator of Erk signaling and subsequent differentiation (Kunath et al., 2007), and *Tcf7l1* (*Tcf3*), a repressor of naive pluripotency (Martello et al., 2012, Wray et al., 2011, Yi et al., 2008). Consequently, naive pluripotency factors are abruptly eliminated upon implantation. These data are inconsistent with the proposition that pluripotency is an inherently precarious balance, wherein pluripotency factors act continuously as competing lineage specifiers (Loh and Lim, 2011). Restriction to preimplantation development precludes these factors from playing a role in lineage specification. We propose that pluripotency is not intrinsically poised for differentiation but progresses through at least three phases: naive, formative, and primed (Kalkan and Smith, 2014). Lineage-specific profiling of the E5.5 epiblast is expected to capture the fundamental attributes of the formative phase.

We uncovered parallels in signaling activity between ESC and diapause. Stabilization of β-catenin via GSK3 inhibition is important for maintenance of naive pluripotency in mouse ESCs (Wray et al., 2011, Ying et al., 2008). Abrogation of *Tcf7l1* function by β-catenin results in stable expression of the key pluripotency factors *Esrrb*, *Klf2*, and *Nanog* (Martello et al., 2012, Wray et al., 2011). This effect of GSK3 inhibition can be partially reproduced by WNT, and together with either LIF stimulation or MAPK/ERK kinase (MEK) inhibition allows continuous propagation of mouse ESCs (Dunn et al., 2014, ten Berge et al., 2011, Wray et al., 2011, Ying et al., 2008). In diapaused epiblasts, we identified high levels of the Jak/Stat downstream targets *Klf4* and *Tfcp2l1* (Bourillot et al., 2009, Martello et al., 2013) and expression of *Lifr* and *Il6st* (*gp130*), reflecting the essential role of LIF signaling in diapause (Nichols et al., 2001). We also observed strong *Wnt4* and *Axin2* expression in diapause, potentially indicative of WNT signaling activity. Because *Tcf7l1* is expressed during diapause, WNT signaling may be important for sustaining naive pluripotency in the dormant epiblast. We speculate that pathways evolved to mediate developmental arrest in vivo may facilitate in vitro capture of the naive state (Nichols et al., 2001, Nichols and Smith, 2012).

In primate embryos, we found the majority of pluripotency-associated genes to be expressed in the ICM. However, absence of KLF2, NR0B1, FBXO15, and BMP4 suggests adaptations in the wider pluripotency network. Similar patterns are observed in human embryos (Blakeley et al., 2015, Yan et al., 2013), lending support for a high degree of conservation of core regulatory interactions in primates. Indeed, the majority of human epiblast-specific factors including KLF17, LEFTY1, and NODAL (Blakeley et al., 2015) are also expressed in the late marmoset ICM. These data confirm similarities between human and marmoset, and highlight the relevance of tractable nonhuman primate species as a model for early development.

Transcriptional data revealed dissimilar expression of FGF, WNT, and TGF-β/Nodal pathway genes. In human, there are conflicting reports regarding the role of TGF-β/Nodal signaling in the embryo (Blakeley et al., 2015, Van der Jeught et al., 2014). We show that NANOG expression in the marmoset ICM does not require FGF, WNT, and TGF-β/Nodal signaling. In particular, we noted an increase in NANOG-positive cells when ERK activation is inhibited by PD03. Robust expression of *Nanog* in the absence of FGF/ERK signaling is reported in a variety of species, including mouse, rat, bovine, and human blastocysts (Kuijk et al., 2012, Nichols et al., 2009, Roode et al., 2012), and may present a general feature of naive pluripotency in mammals. Recent advances in the generation of naive pluripotent human ESCs (Takashima et al., 2014, Theunissen et al., 2014) provide support for this hypothesis.

Suppression of MEK via PD03 blocks PrE formation in rodent embryos (Arman et al., 1998, Kuijk et al., 2012, Nichols et al., 2009, Ralston and Rossant, 2005, Roode et al., 2012, Yamanaka et al., 2010). This effect seems to be attenuated in human (Kuijk et al., 2012, Roode et al., 2012), suggesting involvement of additional mechanisms. We found that WNT inhibition increased NANOG, GATA6, and CDX2 in marmoset embryos, leading to a blurring of lineage boundaries. Cells failing to undergo lineage specification may remain trapped at an earlier developmental stage when NANOG and GATA6 are coexpressed. Interference with ERK signaling reduced the number of PrE cells and broadened expression of CDX2 and NANOG. We propose that lineage specification in primate preimplantation development is regulated by both WNT and FGF/ERK pathways, in contrast to mouse, where FGF/ERK is the primary and sufficient driver (Figure 7E).

This study provides a comprehensive resource for identifying the factors and pathways that ignite and extinguish naive pluripotency in vivo. Knowledge gained should be valuable for evaluating and optimizing ESC differentiation and reprogramming to pluripotency in vitro. A further application may be to influence lineage decisions in embryo culture in order to facilitate stem cell derivation or even to improve human blastocyst development for assisted conception.

## Experimental Procedures

### Mouse Strains and Embryo Collection

Mice used were intercrosses of *Pdgfra::GFP* (Hamilton et al., 2003) and strain 129 estrus-checked females. Embryos were collected at the relevant stages from the oviduct or uterus in M2 medium (Sigma). Embryonic diapause was induced by surgical removal of both ovaries on the morning of the third day of pregnancy (E2.5). Diapaused embryos were flushed on the seventh day of pregnancy. Experiments were performed in accordance with EU guidelines for the care and use of laboratory animals, and under authority of UK governmental legislation. Use of animals in this project was approved by the ethical review committee for the University of Cambridge, and relevant Home Office licenses are in place.

### Marmoset Colony Maintenance and Embryo Collection

Primate embryos were obtained from the German Primate Center, Göttingen (Deutsches Primatenzentrum; DPZ) and the Central Institute for Experimental Animals, Kanagawa, Japan (CIEA). Marmoset blastocysts were retrieved by nonsurgical uterine flush according to established methods using recently developed devices (Takahashi et al., 2014, Thomson et al., 1994). Because the time of conception must be determined retrospectively and with an accuracy of ±24 hr, we applied additional criteria to embryo staging such as blastocoel formation and diameter (Figure 5A). Staging of female reproductive cycles and protocols for embryo retrieval have been described (Hanazawa et al., 2012). Animals were obtained from self-sustaining colonies and housed according to standard husbandry guidelines. Protocols for the use of animals and institutional regulations for the care and experimental use of marmosets were strictly followed. Experiments at the DPZ were conducted under license number AZ 33.42502–066/06. Experiments using marmosets at the CIEA were approved by the animal research committee (CIEA 11028) and performed in compliance with guidelines set forth by the Science Council of Japan.

### Isolation of Single-Cell Suspensions

Mouse (E3.25–E4.5) and marmoset blastocysts were subjected to immunosurgery as previously described (Nichols et al., 1998, Solter and Knowles, 1975). ICMs were subsequently dissociated from residual trophectoderm with a narrowly fitting Pasteur pipette. For postimplantation mouse embryos, the epiblast was isolated by manual dissection. Dissociation of morulae, ICM, and postimplantation epiblast was carried out in a mixture of trypsin and chick serum (see Supplemental Experimental Procedures). Cells were dissociated by repetitive pipetting using blunted microcapillaries.

### Embryonic Stem Cell Culture

E14TG2a cells derived from mouse strain 129/Ola (Hooper et al., 1987) were used as a reference ESC line and cultured in 2i/LIF conditions (Ying et al., 2008). N2B27 (1:1 DMEM/F-12 and Neurobasal media, N2 [in-house] and B27 [GIBCO] additives, 2 mM L-glutamine, and 100 μM β-mercaptoethanol) was supplemented with 1 μM PD0325901, 3 μM CHIR99021, and 10 ng/ml LIF (in-house), and cells were maintained in gelatin-coated (0.1%) culture vessels. Cells were dissociated by conventional methods, and 20 cells per sample were manually selected with a blunt microcapillary.

### Transcriptome Analysis

Library construction was carried out using whole-transcriptome preamplification (Tang et al., 2009, Tang et al., 2010b) followed by sonication of cDNA and preparation of Illumina-compatible sequencing constructs (see Supplemental Experimental Procedures). Sequencing reads were processed to remove preamplification adapters and were aligned with GSNAP (Wu and Nacu, 2010). Global analyses were based on variance-stabilized counts computed with the Bioconductor package DESeq (Anders and Huber, 2010). Differential expression analysis was performed in DESeq. Gene clusters were identified by hierarchical clustering on scaled FPKMs. The GOstats R package (Falcon and Gentleman, 2007) was used for GO category and KEGG pathway enrichment analysis. High-level comparative analysis of KEGG pathways was based on the mean expression of constituent genes. For analysis of individual pathways, expression values were mapped onto pathway nodes using PathVisio (Kutmon et al., 2015). PluriNet82 was created by intersecting PluriNetWork (Som et al., 2010) with dynamically expressed genes. Preferential activity at a given embryonic stage was defined as positive-scaled log-normalized expression for a network node; a network edge was required to have active source and target nodes. Cytoscape (Smoot et al., 2011) was used for network visualization.

## Author Contributions

P.B., A.S., and J.N. conceived the study; T.B., P.B., and J.N. developed the experimental approach and embryo sequencing workflow; T.B. and P.B. processed samples and prepared sequencing libraries; R.L., P.L., and P.B. performed computational analyses; T.B. and J.O. carried out marmoset embryo experiments under the guidance of E.S. and R.B.; T.B., R.L., A.S., and P.B. wrote the paper.

## Accession Numbers

The accession numbers for the mouse and marmoset sequencing data reported in this paper are ArrayExpress: E-MTAB-2958, E-MTAB-2959.

## Supplemental Information

Document S1. Supplemental Experimental Procedures and Figures S1–S7

Table S1. Mouse Gene Expression Levels for the Developmental Stages and Samples Analyzed in Fragments per Kilobase of Exon per Million Fragments Mapped, Related to Figure 1

Table S2. Dynamically Expressed Genes in Mouse Embryonic Lineages during Development from the E2.5 Morula Stage to E5.5 Postimplantation, Related to Figure 2

Table S3. Constitutively Expressed Genes in Mouse Development from Morula to Postimplantation Stages, Related to Figure 2

Table S4. Mouse Gene Expression Modules for the Developmental Stages and Samples Analyzed; Modules Were Derived by Hierarchical Clustering of Scaled Expression Values, Related to Figure 2

Table S5. Genes Differentially Expressed between Pairs of Conditions in Mouse Embryonic Development, Diapaused Epiblasts, and Cultured ESCs, Related to Figure 2

Table S6. Marmoset Gene Expression Levels over the Developmental Stages Analyzed in FPKMs, Related to Figure 5

Table S7. Comparison of Mouse and Marmoset ICM; Embryo-Matched Lineage-Specific Data from Mouse Epiblast and PrE Samples Were Merged for Compatibility with Marmoset ICM, Related to Figure 5

Document S2. Article plus Supplemental Information

## References

[bib1] Adewumi O., Aflatoonian B., Ahrlund-Richter L., Amit M., Andrews P.W., Beighton G., Bello P.A., Benvenisty N., Berry L.S., Bevan S., International Stem Cell Initiative (2007). Characterization of human embryonic stem cell lines by the International Stem Cell Initiative. Nat. Biotechnol..

[bib2] Anders S., Huber W. (2010). Differential expression analysis for sequence count data. Genome Biol..

[bib3] Arman E., Haffner-Krausz R., Chen Y., Heath J.K., Lonai P. (1998). Targeted disruption of fibroblast growth factor (FGF) receptor 2 suggests a role for FGF signaling in pregastrulation mammalian development. Proc. Natl. Acad. Sci. USA.

[bib4] Artus J., Piliszek A., Hadjantonakis A.K. (2011). The primitive endoderm lineage of the mouse blastocyst: sequential transcription factor activation and regulation of differentiation by Sox17. Dev. Biol..

[bib5] Bedzhov I., Zernicka-Goetz M. (2014). Self-organizing properties of mouse pluripotent cells initiate morphogenesis upon implantation. Cell.

[bib6] Biechele S., Cockburn K., Lanner F., Cox B.J., Rossant J. (2013). Porcn-dependent Wnt signaling is not required prior to mouse gastrulation. Development.

[bib7] Blakeley P., Fogarty N.M., Del Valle I., Wamaitha S.E., Hu T.X., Elder K., Snell P., Christie L., Robson P., Niakan K.K. (2015). Defining the three cell lineages of the human blastocyst by single-cell RNA-seq. Development.

[bib8] Boroviak T., Loos R., Bertone P., Smith A., Nichols J. (2014). The ability of inner-cell-mass cells to self-renew as embryonic stem cells is acquired following epiblast specification. Nat. Cell Biol..

[bib9] Bourillot P.Y., Aksoy I., Schreiber V., Wianny F., Schulz H., Hummel O., Hubner N., Savatier P. (2009). Novel STAT3 target genes exert distinct roles in the inhibition of mesoderm and endoderm differentiation in cooperation with Nanog. Stem Cells.

[bib10] Brennecke P., Anders S., Kim J.K., Kołodziejczyk A.A., Zhang X., Proserpio V., Baying B., Benes V., Teichmann S.A., Marioni J.C., Heisler M.G. (2013). Accounting for technical noise in single-cell RNA-seq experiments. Nat. Methods.

[bib11] Brons I.G., Smithers L.E., Trotter M.W., Rugg-Gunn P., Sun B., Chuva de Sousa Lopes S.M., Howlett S.K., Clarkson A., Ahrlund-Richter L., Pedersen R.A., Vallier L. (2007). Derivation of pluripotent epiblast stem cells from mammalian embryos. Nature.

[bib12] Brook F.A., Gardner R.L. (1997). The origin and efficient derivation of embryonic stem cells in the mouse. Proc. Natl. Acad. Sci. USA.

[bib13] Buehr M., Meek S., Blair K., Yang J., Ure J., Silva J., McLay R., Hall J., Ying Q.L., Smith A. (2008). Capture of authentic embryonic stem cells from rat blastocysts. Cell.

[bib14] Chan Y.S., Göke J., Ng J.H., Lu X., Gonzales K.A., Tan C.P., Tng W.Q., Hong Z.Z., Lim Y.S., Ng H.H. (2013). Induction of a human pluripotent state with distinct regulatory circuitry that resembles preimplantation epiblast. Cell Stem Cell.

[bib15] Chazaud C., Yamanaka Y., Pawson T., Rossant J. (2006). Early lineage segregation between epiblast and primitive endoderm in mouse blastocysts through the Grb2-MAPK pathway. Dev. Cell.

[bib16] Dunn S.J., Martello G., Yordanov B., Emmott S., Smith A.G. (2014). Defining an essential transcription factor program for naïve pluripotency. Science.

[bib17] Evans M.J., Kaufman M.H. (1981). Establishment in culture of pluripotential cells from mouse embryos. Nature.

[bib18] Falcon S., Gentleman R. (2007). Using GOstats to test gene lists for GO term association. Bioinformatics.

[bib19] Fang R., Liu K., Zhao Y., Li H., Zhu D., Du Y., Xiang C., Li X., Liu H., Miao Z. (2014). Generation of naive induced pluripotent stem cells from rhesus monkey fibroblasts. Cell Stem Cell.

[bib20] Frum T., Halbisen M.A., Wang C., Amiri H., Robson P., Ralston A. (2013). Oct4 cell-autonomously promotes primitive endoderm development in the mouse blastocyst. Dev. Cell.

[bib21] Gerbe F., Cox B., Rossant J., Chazaud C. (2008). Dynamic expression of Lrp2 pathway members reveals progressive epithelial differentiation of primitive endoderm in mouse blastocyst. Dev. Biol..

[bib22] Grün D., Kester L., van Oudenaarden A. (2014). Validation of noise models for single-cell transcriptomics. Nat. Methods.

[bib23] Guo G., Huss M., Tong G.Q., Wang C., Li Sun L., Clarke N.D., Robson P. (2010). Resolution of cell fate decisions revealed by single-cell gene expression analysis from zygote to blastocyst. Dev. Cell.

[bib24] Hall J., Guo G., Wray J., Eyres I., Nichols J., Grotewold L., Morfopoulou S., Humphreys P., Mansfield W., Walker R. (2009). Oct4 and LIF/Stat3 additively induce Krüppel factors to sustain embryonic stem cell self-renewal. Cell Stem Cell.

[bib25] Hamilton T.G., Klinghoffer R.A., Corrin P.D., Soriano P. (2003). Evolutionary divergence of platelet-derived growth factor alpha receptor signaling mechanisms. Mol. Cell. Biol..

[bib26] Hanazawa K., Mueller T., Becker T., Heistermann M., Behr R., Sasaki E. (2012). Minimally invasive transabdominal collection of preimplantation embryos from the common marmoset monkey (*Callithrix jacchus*). Theriogenology.

[bib27] Handyside A.H. (1978). Time of commitment of inside cells isolated from preimplantation mouse embryos. J. Embryol. Exp. Morphol..

[bib28] Hooper M., Hardy K., Handyside A., Hunter S., Monk M. (1987). HPRT-deficient (Lesch-Nyhan) mouse embryos derived from germline colonization by cultured cells. Nature.

[bib29] Kalkan T., Smith A. (2014). Mapping the route from naive pluripotency to lineage specification. Philos. Trans. R. Soc. Lond. B Biol. Sci..

[bib30] Ketchel M.M., Banik U.K., Mantalenakis S.J. (1966). A study of delayed implantation caused by parabiosis in pregnant rats. J. Reprod. Fertil..

[bib31] Kharchenko P.V., Silberstein L., Scadden D.T. (2014). Bayesian approach to single-cell differential expression analysis. Nat. Methods.

[bib32] Kishi N., Sato K., Sasaki E., Okano H. (2014). Common marmoset as a new model animal for neuroscience research and genome editing technology. Dev. Growth Differ..

[bib33] Kuijk E.W., van Tol L.T., Van de Velde H., Wubbolts R., Welling M., Geijsen N., Roelen B.A. (2012). The roles of FGF and MAP kinase signaling in the segregation of the epiblast and hypoblast cell lineages in bovine and human embryos. Development.

[bib34] Kunath T., Saba-El-Leil M.K., Almousailleakh M., Wray J., Meloche S., Smith A. (2007). FGF stimulation of the Erk1/2 signalling cascade triggers transition of pluripotent embryonic stem cells from self-renewal to lineage commitment. Development.

[bib35] Kurimoto K., Yabuta Y., Ohinata Y., Ono Y., Uno K.D., Yamada R.G., Ueda H.R., Saitou M. (2006). An improved single-cell cDNA amplification method for efficient high-density oligonucleotide microarray analysis. Nucleic Acids Res..

[bib36] Kutmon M., van Iersel M.P., Bohler A., Kelder T., Nunes N., Pico A.R., Evelo C.T. (2015). PathVisio 3: an extendable pathway analysis toolbox. PLoS Comput. Biol..

[bib37] Lafon S., Keller Y., Coifman R.R. (2006). Data fusion and multicue data matching by diffusion maps. IEEE Trans. Pattern Anal. Mach. Intell..

[bib38] Le Bin G.C., Muñoz-Descalzo S., Kurowski A., Leitch H., Lou X., Mansfield W., Etienne-Dumeau C., Grabole N., Mulas C., Niwa H. (2014). Oct4 is required for lineage priming in the developing inner cell mass of the mouse blastocyst. Development.

[bib39] Lee H.J., Hore T.A., Reik W. (2014). Reprogramming the methylome: erasing memory and creating diversity. Cell Stem Cell.

[bib40] Leitch H.G., McEwen K.R., Turp A., Encheva V., Carroll T., Grabole N., Mansfield W., Nashun B., Knezovich J.G., Smith A. (2013). Naive pluripotency is associated with global DNA hypomethylation. Nat. Struct. Mol. Biol..

[bib41] Li P., Tong C., Mehrian-Shai R., Jia L., Wu N., Yan Y., Maxson R.E., Schulze E.N., Song H., Hsieh C.L. (2008). Germline competent embryonic stem cells derived from rat blastocysts. Cell.

[bib42] Loh K.M., Lim B. (2011). A precarious balance: pluripotency factors as lineage specifiers. Cell Stem Cell.

[bib43] Lustig B., Jerchow B., Sachs M., Weiler S., Pietsch T., Karsten U., van de Wetering M., Clevers H., Schlag P.M., Birchmeier W., Behrens J. (2002). Negative feedback loop of Wnt signaling through upregulation of conductin/axin2 in colorectal and liver tumors. Mol. Cell. Biol..

[bib44] Mantalenakis S.J., Ketchel M.M. (1966). Frequency and extent of delayed implantation in lactating rats and mice. J. Reprod. Fertil..

[bib45] Marks H., Kalkan T., Menafra R., Denissov S., Jones K., Hofemeister H., Nichols J., Kranz A., Stewart A.F., Smith A., Stunnenberg H.G. (2012). The transcriptional and epigenomic foundations of ground state pluripotency. Cell.

[bib46] Martello G., Sugimoto T., Diamanti E., Joshi A., Hannah R., Ohtsuka S., Göttgens B., Niwa H., Smith A. (2012). Esrrb is a pivotal target of the Gsk3/Tcf3 axis regulating embryonic stem cell self-renewal. Cell Stem Cell.

[bib47] Martello G., Bertone P., Smith A. (2013). Identification of the missing pluripotency mediator downstream of leukaemia inhibitory factor. EMBO J..

[bib48] Martin G.R. (1981). Isolation of a pluripotent cell line from early mouse embryos cultured in medium conditioned by teratocarcinoma stem cells. Proc. Natl. Acad. Sci. USA.

[bib49] Matter K., Balda M.S. (1999). Occludin and the functions of tight junctions. Int. Rev. Cytol..

[bib50] Mead R.A. (1993). Embryonic diapause in vertebrates. J. Exp. Zool..

[bib51] Nakatsuji N., Suemori H. (2002). Embryonic stem cell lines of nonhuman primates. ScientificWorldJournal.

[bib52] Nichols J., Gardner R.L. (1984). Heterogeneous differentiation of external cells in individual isolated early mouse inner cell masses in culture. J. Embryol. Exp. Morphol..

[bib53] Nichols J., Smith A. (2009). Naive and primed pluripotent states. Cell Stem Cell.

[bib54] Nichols J., Smith A. (2012). Pluripotency in the embryo and in culture. Cold Spring Harb. Perspect. Biol..

[bib55] Nichols J., Zevnik B., Anastassiadis K., Niwa H., Klewe-Nebenius D., Chambers I., Schöler H., Smith A. (1998). Formation of pluripotent stem cells in the mammalian embryo depends on the POU transcription factor Oct4. Cell.

[bib56] Nichols J., Chambers I., Taga T., Smith A. (2001). Physiological rationale for responsiveness of mouse embryonic stem cells to gp130 cytokines. Development.

[bib57] Nichols J., Silva J., Roode M., Smith A. (2009). Suppression of Erk signalling promotes ground state pluripotency in the mouse embryo. Development.

[bib97] Niwa H., Ogawa K., Shimosato D., Adachi K. (2009). A parallel circuit of LIF signalling pathways maintains pluripotency of mouse ES cells. Nature.

[bib58] Ohnishi Y., Huber W., Tsumura A., Kang M., Xenopoulos P., Kurimoto K., Oleś A.K., Araúzo-Bravo M.J., Saitou M., Hadjantonakis A.K., Hiiragi T. (2014). Cell-to-cell expression variability followed by signal reinforcement progressively segregates early mouse lineages. Nat. Cell Biol..

[bib59] Palmieri S.L., Peter W., Hess H., Schöler H.R. (1994). Oct-4 transcription factor is differentially expressed in the mouse embryo during establishment of the first two extraembryonic cell lineages involved in implantation. Dev. Biol..

[bib60] Pilon N., Oh K., Sylvestre J.R., Savory J.G., Lohnes D. (2007). Wnt signaling is a key mediator of Cdx1 expression in vivo. Development.

[bib61] Plusa B., Piliszek A., Frankenberg S., Artus J., Hadjantonakis A.K. (2008). Distinct sequential cell behaviours direct primitive endoderm formation in the mouse blastocyst. Development.

[bib62] Ralston A., Rossant J. (2005). Genetic regulation of stem cell origins in the mouse embryo. Clin. Genet..

[bib63] Renfree M.B., Shaw G. (2000). Diapause. Annu. Rev. Physiol..

[bib64] Roode M., Blair K., Snell P., Elder K., Marchant S., Smith A., Nichols J. (2012). Human hypoblast formation is not dependent on FGF signalling. Dev. Biol..

[bib65] Rossant J., Lis W.T. (1979). Potential of isolated mouse inner cell masses to form trophectoderm derivatives in vivo. Dev. Biol..

[bib66] Schrode N., Saiz N., Di Talia S., Hadjantonakis A.K. (2014). GATA6 levels modulate primitive endoderm cell fate choice and timing in the mouse blastocyst. Dev. Cell.

[bib67] Shin K., Fogg V.C., Margolis B. (2006). Tight junctions and cell polarity. Annu. Rev. Cell Dev. Biol..

[bib68] Sleeman J.P., Thiery J.P. (2011). SnapShot: the epithelial-mesenchymal transition. Cell.

[bib69] Smith Z.D., Chan M.M., Mikkelsen T.S., Gu H., Gnirke A., Regev A., Meissner A. (2012). A unique regulatory phase of DNA methylation in the early mammalian embryo. Nature.

[bib70] Smoot M.E., Ono K., Ruscheinski J., Wang P.L., Ideker T. (2011). Cytoscape 2.8: new features for data integration and network visualization. Bioinformatics.

[bib71] Solter D., Knowles B.B. (1975). Immunosurgery of mouse blastocyst. Proc. Natl. Acad. Sci. USA.

[bib72] Som A., Harder C., Greber B., Siatkowski M., Paudel Y., Warsow G., Cap C., Schöler H., Fuellen G. (2010). The PluriNetWork: an electronic representation of the network underlying pluripotency in mouse, and its applications. PLoS ONE.

[bib73] Tachibana M., Sparman M., Ramsey C., Ma H., Lee H.S., Penedo M.C., Mitalipov S. (2012). Generation of chimeric rhesus monkeys. Cell.

[bib74] Takahashi T., Hanazawa K., Inoue T., Sato K., Sedohara A., Okahara J., Suemizu H., Yagihashi C., Yamamoto M., Eto T. (2014). Birth of healthy offspring following ICSI in in vitro-matured common marmoset (*Callithrix jacchus*) oocytes. PLoS ONE.

[bib75] Takashima Y., Guo G., Loos R., Nichols J., Ficz G., Krueger F., Oxley D., Santos F., Clarke J., Mansfield W. (2014). Resetting transcription factor control circuitry toward ground-state pluripotency in human. Cell.

[bib76] Tang F., Barbacioru C., Wang Y., Nordman E., Lee C., Xu N., Wang X., Bodeau J., Tuch B.B., Siddiqui A. (2009). mRNA-seq whole-transcriptome analysis of a single cell. Nat. Methods.

[bib77] Tang F., Barbacioru C., Bao S., Lee C., Nordman E., Wang X., Lao K., Surani M.A. (2010). Tracing the derivation of embryonic stem cells from the inner cell mass by single-cell RNA-seq analysis. Cell Stem Cell.

[bib78] Tang F., Barbacioru C., Nordman E., Li B., Xu N., Bashkirov V.I., Lao K., Surani M.A. (2010). RNA-seq analysis to capture the transcriptome landscape of a single cell. Nat. Protoc..

[bib79] ten Berge D., Kurek D., Blauwkamp T., Koole W., Maas A., Eroglu E., Siu R.K., Nusse R. (2011). Embryonic stem cells require Wnt proteins to prevent differentiation to epiblast stem cells. Nat. Cell Biol..

[bib80] Tesar P.J., Chenoweth J.G., Brook F.A., Davies T.J., Evans E.P., Mack D.L., Gardner R.L., McKay R.D. (2007). New cell lines from mouse epiblast share defining features with human embryonic stem cells. Nature.

[bib81] Theunissen T.W., Powell B.E., Wang H., Mitalipova M., Faddah D.A., Reddy J., Fan Z.P., Maetzel D., Ganz K., Shi L. (2014). Systematic identification of culture conditions for induction and maintenance of naive human pluripotency. Cell Stem Cell.

[bib82] Thomson J.A., Kalishman J., Hearn J.P. (1994). Nonsurgical uterine stage preimplantation embryo collection from the common marmoset. J. Med. Primatol..

[bib83] Thomson J.A., Kalishman J., Golos T.G., Durning M., Harris C.P., Hearn J.P. (1996). Pluripotent cell lines derived from common marmoset (*Callithrix jacchus*) blastocysts. Biol. Reprod..

[bib84] Thomson J.A., Itskovitz-Eldor J., Shapiro S.S., Waknitz M.A., Swiergiel J.J., Marshall V.S., Jones J.M. (1998). Embryonic stem cell lines derived from human blastocysts. Science.

[bib85] Turksen K., Troy T.C. (2001). Claudin-6: a novel tight junction molecule is developmentally regulated in mouse embryonic epithelium. Dev. Dyn..

[bib86] Van der Jeught M., Heindryckx B., O’Leary T., Duggal G., Ghimire S., Lierman S., Van Roy N., Chuva de Sousa Lopes S.M., Deroo T., Deforce D., De Sutter P. (2014). Treatment of human embryos with the TGFβ inhibitor SB431542 increases epiblast proliferation and permits successful human embryonic stem cell derivation. Hum. Reprod..

[bib87] Weitlauf H.M., Greenwald G.S. (1968). Survival of blastocysts in the uteri of ovariectomized mice. J. Reprod. Fertil..

[bib88] Wray J., Kalkan T., Gomez-Lopez S., Eckardt D., Cook A., Kemler R., Smith A. (2011). Inhibition of glycogen synthase kinase-3 alleviates Tcf3 repression of the pluripotency network and increases embryonic stem cell resistance to differentiation. Nat. Cell Biol..

[bib89] Wu T.D., Nacu S. (2010). Fast and SNP-tolerant detection of complex variants and splicing in short reads. Bioinformatics.

[bib90] Xue Z., Huang K., Cai C., Cai L., Jiang C.Y., Feng Y., Liu Z., Zeng Q., Cheng L., Sun Y.E. (2013). Genetic programs in human and mouse early embryos revealed by single-cell RNA sequencing. Nature.

[bib91] Yamanaka Y., Lanner F., Rossant J. (2010). FGF signal-dependent segregation of primitive endoderm and epiblast in the mouse blastocyst. Development.

[bib92] Yan L., Yang M., Guo H., Yang L., Wu J., Li R., Liu P., Lian Y., Zheng X., Yan J. (2013). Single-cell RNA-seq profiling of human preimplantation embryos and embryonic stem cells. Nat. Struct. Mol. Biol..

[bib93] Ye S., Li P., Tong C., Ying Q.L. (2013). Embryonic stem cell self-renewal pathways converge on the transcription factor Tfcp2l1. EMBO J..

[bib94] Yi F., Pereira L., Merrill B.J. (2008). Tcf3 functions as a steady-state limiter of transcriptional programs of mouse embryonic stem cell self-renewal. Stem Cells.

[bib95] Yi F., Pereira L., Hoffman J.A., Shy B.R., Yuen C.M., Liu D.R., Merrill B.J. (2011). Opposing effects of Tcf3 and Tcf1 control Wnt stimulation of embryonic stem cell self-renewal. Nat. Cell Biol..

[bib96] Ying Q.L., Wray J., Nichols J., Batlle-Morera L., Doble B., Woodgett J., Cohen P., Smith A. (2008). The ground state of embryonic stem cell self-renewal. Nature.

