# Supplementary material for: Lineage-Specific Profiling Delineates the Emergence and Progression of Naive Pluripotency in Mammalian Embryogenesis
Source: Dev Cell. 2015 Nov 9;35(3):366–82. doi: 10.1016/j.devcel.2015.10.011 (PMC4643313; doi:10.1016/j.devcel.2015.10.011)
Supplement: Document S1. Supplemental Experimental Procedures and Figures S1–S7 [file mmc1.pdf]

Developmental Cell

Supplemental Information

Lineage-Specific Profiling Delineates the Emergence and Progression of Naive Pluripotency in Mammalian Embryogenesis

Thorsten Boroviak, Remco Loos, Patrick Lombard, Junko Okahara, Rüdiger Behr, Erika Sasaki, Jennifer Nichols, Austin Smith, and Paul Bertone

Supplemental Figures

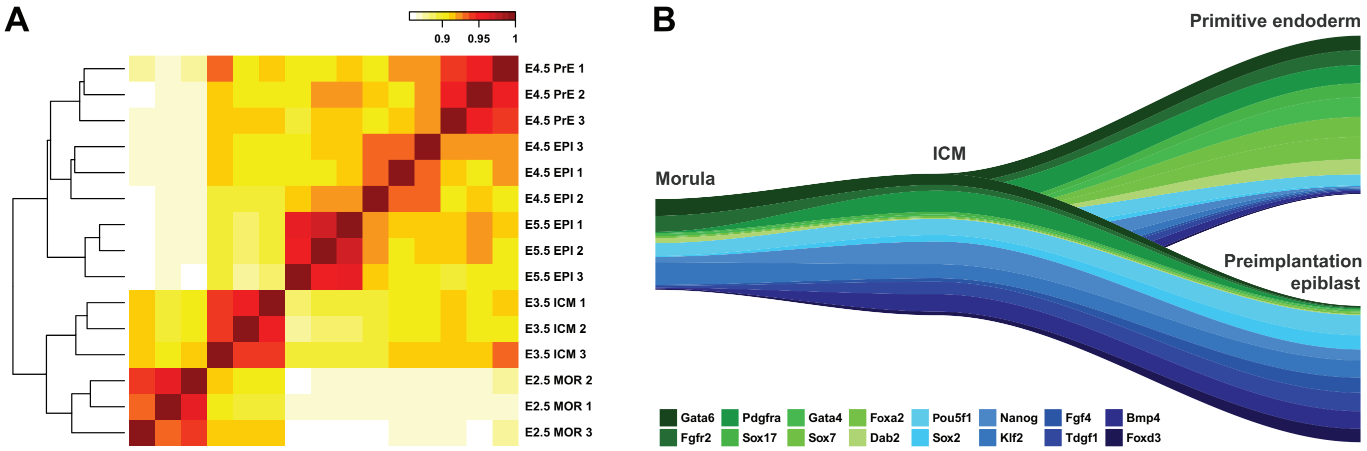

Figure S1, related to Figure 1: (A) Global correlation of mouse embryonic samples. (B) Stream plot of 16 lineage markers for PrE (green) and preimplantation epiblast (blue). Track widths represent the relative expression of the gene normalized to the mean expression across the developmental stages displayed. Genes specifically expressed in PrE or preimplantation epiblast include all established lineage markers. The early PrE markers *Gata6* and *Fgfr2* were expressed from the morula stage, with upregulation of *Pdgfra* in the early ICM. Late PrE markers, including *Gata4*, *Sox7*, *Foxa2* and *Dab2*, were specifically upregulated in PrE at E4.5, with *Gata6*, *Fgfr2* and *Pdgfra* expression maintained, consistent with the proposed sequential activation of Gata6, Sox17, Gata4 and Sox7 during PrE specification (Artus et al., 2011) and expression patterns observed by others (Chazaud et al., 2006; Gerbe et al., 2008; Guo et al., 2010; Ohnishi et al., 2014). Conversely, preimplantation epiblast cells exhibited robust expression of epiblast-specific genes, including *Sox2*, *Nanog*, *Klf2*, *Bmp4* and *Fgf4* (Guo et al., 2010; Kurimoto et al., 2006; Tang et al., 2010a).

A

MOR

Term	P-val. Adj (BH)
amine metabolic process	0.02269575
cellular biogenic amine metabolic process	0.02269575
cellular amine metabolic process	0.0292063
in utero embryonic development	0.04885411

MOR, ICM

Term	P-val. Adj (BH)
organ development	5.065E-05
multicellular organismal process	0.0004266
system development	0.0004266
regulation of cell differentiation	0.00494365
tissue development	0.00641332
regulation of multicellular organismal process	0.00641332
multicellular organismal development	0.00641332
regulation of developmental process	0.01695561
cell differentiation	0.02443758
cell development	0.02450709
anatomical structure development	0.02478382
regulation of multicellular organismal development	0.04258611
positive regulation of developmental process	0.0484731
small molecule biosynthetic process	0.04881227
response to external stimulus	0.05219916
cellular developmental process	0.05219916

MOR, ICM, pre-EPI

Term	P-val. Adj (BH)
detoxification of copper ion	0.01028629
nitric oxide mediated signal transduction	0.01537011
regulation of humoral immune response	0.01537011
response to copper ion	0.01537011
regulation of autophagy	0.02056179
regulation of acute inflammatory response	0.02176294
regulation of developmental growth	0.02176294
regulation of response to external stimulus	0.02176294
humoral immune response	0.02176294
response to external stimulus	0.02176294
developmental growth	0.02571741
negative regulation of autophagy	0.03044577
cellular zinc ion homeostasis	0.04756802
zinc ion homeostasis	0.04756802
positive regulation of developmental process	0.04909514
humoral immune response	0.04909514
response to metal ion	0.04940919
positive regulation of developmental growth	0.04940919
autophagy	0.04940919
acute inflammatory response	0.04940919
cellular response to metal ion	0.04940919
cellular divalent inorganic cation homeostasis	0.04940919
response to toxin	0.04940919
cellular response to inorganic substance	0.04940919
divalent inorganic cation homeostasis	0.04940919
cellular response to chemical stimulus	0.04940919
immunoglobulin mediated immune response	0.04940919
B cell mediated immunity	0.04940919
cell projection assembly	0.04940919
second-messenger-mediated signaling	0.04940919
positive regulation of transmembrane transport	0.04940919
growth	0.04940919
developmental cell growth	0.04940919

ICM

Term	P-val. Adj (BH)
response to chemical stimulus	0.00052285
response to wounding	0.00175971
intracellular protein kinase cascade	0.00175971
cellular response to chemical stimulus	0.00175971
cell adhesion	0.00412043
biological adhesion	0.00412043
response to stimulus	0.00461262
odontogenesis	0.00461262
cellular response to organic substance	0.00764043
regulation of multicellular organismal process	0.00865531
regulation of intracellular protein kinase cascade	0.00865531
multicellular organismal process	0.00977462
organ development	0.01234617
cell communication	0.01375272
phospholipid homeostasis	0.01375272
defense response	0.01375272
response to external stimulus	0.01458803
response to organic substance	0.01458803
inflammatory response	0.01458803
regulation of response to stress	0.01642693
cell-cell adhesion	0.01642693
gland development	0.01642693
odontogenesis of dentin-containing tooth	0.01642693
response to metal ion	0.01646228
MAPK cascade	0.01714087
cytokine production	0.01857364
system development	0.01999361

ICM, preiml. EPI

Term	P-val. Adj (BH)
response to chemical stimulus	0.00010514
multicellular organismal process	0.00013049
response to stimulus	0.00254199
cellular response to organic substance	0.00254199
response to organic substance	0.00254199
developmental process	0.00254199
anatomical structure development	0.00297988
cell migration	0.00323982
cellular response to chemical stimulus	0.00347489
cell motility	0.00585354
localization of cell	0.00585354
multicellular organismal development	0.00698981
cellular response to cytokine stimulus	0.00839442
anatomical structure morphogenesis	0.00839442
cell-cell signaling	0.00839442
immune system process	0.00839442
negative regulation of multicellular organismal process	0.00839442
secretion	0.00839442
segmentation	0.00839442
cytokine secretion	0.00839442
regulation of cell migration	0.00948099
secretion by cell	0.00948099
BMP signaling pathway	0.00991232
tissue development	0.01001977
response to nutrient	0.0102139
polysaccharide metabolic process	0.01024069
regulation of cell motility	0.01129983
cell activation	0.01129983
locomotion	0.01129983
regulation of locomotion	0.01214547
regulation of multicellular organismal process	0.01214547

ICM, pre-EPI, postEPI

Term	P-val. Adj (BH)
oxidation-reduction process	0.00796682

preimpl. EPI

Term	P-val. Adj (BH)
multicellular organismal process	0.00029974
high-density lipoprotein particle clearance	0.01591473
developmental process	0.01591473
multicellular organismal development	0.02802533
anatomical structure development	0.03692507
trophoblast cell migration	0.03692507
regulation of trophoblast cell migration	0.03692507
negative regulation of trophoblast cell migration	0.03692507
negative regulation of cell migration	0.03692507
negative regulation of cell motility	0.04204448
negative regulation of locomotion	0.04775401
negative regulation of cellular component movement	0.04871758
trophectodermal cellular morphogenesis	0.05865734
negative regulation of platelet activation	0.05865734
chylomicron remnant clearance	0.05865734
triglyceride-rich lipoprotein particle clearance	0.05865734
blood vessel development	0.05865734
system development	0.05865734
regulation of tube size	0.05865734
regulation of blood vessel size	0.05865734
embryo implantation	0.06594336

preimpl. EPI, postimpl. EPI

Term	P-val. Adj (BH)
response to chemical stimulus	0.00516425
cell proliferation	0.00516425
organ morphogenesis	0.00516425
positive regulation of cell development	0.00516425
positive regulation of MAP kinase activity	0.00516425
generation of neurons	0.00516425
neural crest cell migration	0.00516425
anatomical structure morphogenesis	0.00516425
positive regulation of neurogenesis	0.00516425
branching morphogenesis of a tube	0.00516425
cell morphogenesis involved in differentiation	0.00516425
cell death	0.00516425
organ development	0.00516425
cellular response to chemical stimulus	0.00516425
programmed cell death	0.00516425
small molecule metabolic process	0.00516425
death	0.00516425
response to superoxide	0.00516425
response to oxygen radical	0.00516425
negative regulation of cellular process	0.00589999
regulation of developmental process	0.00589999
response to organic substance	0.00589999
neurogenesis	0.00643218
apoptotic process	0.00675768

postimpl. EPI

Term	P-val. Adj (BH)
nervous system development	7.0003E-05
cell differentiation	7.0003E-05
cell communication	7.0003E-05
signaling	7.0003E-05
anatomical structure development	7.0003E-05
system development	9.567E-05
cellular developmental process	0.00015016
multicellular organismal development	0.00015016
positive regulation of biological process	0.00016447
positive regulation of cellular process	0.00016447
regulation of multicellular organismal development	0.00021907
signal transduction	0.00021907
developmental process	0.00022631
neurogenesis	0.00022631
regulation of response to stimulus	0.00032091
multicellular organismal process	0.00032091
cellular response to stimulus	0.00043936
response to stimulus	0.00063096
neuron differentiation	0.00078691
cell surface receptor signaling pathway	0.00079899
regulation of cell differentiation	0.00095642
generation of neurons	0.0016368
regulation of signaling	0.00197571
regulation of nervous system development	0.00253872

B

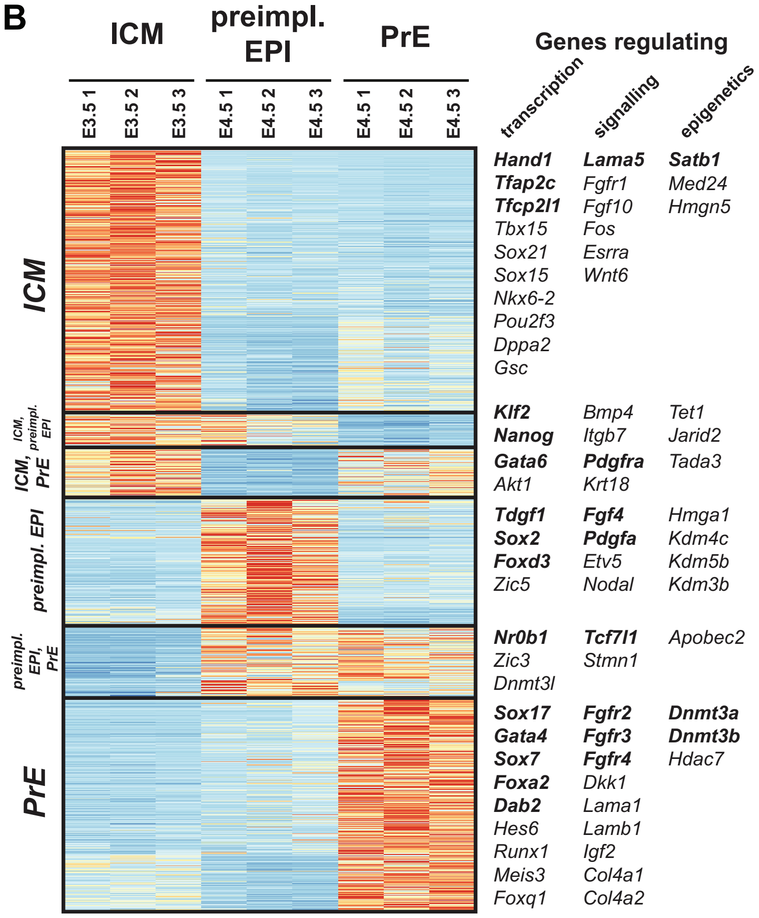

Figure S2, related to Figure 2: (A) GO enrichment for stage-specific expression in early mouse development. (B) Dynamically expressed genes during epiblast and PrE specification. Selected transcription factors, signaling pathway components and epigenetic regulators are shown to the right, with a subset of lineage markers in bold.

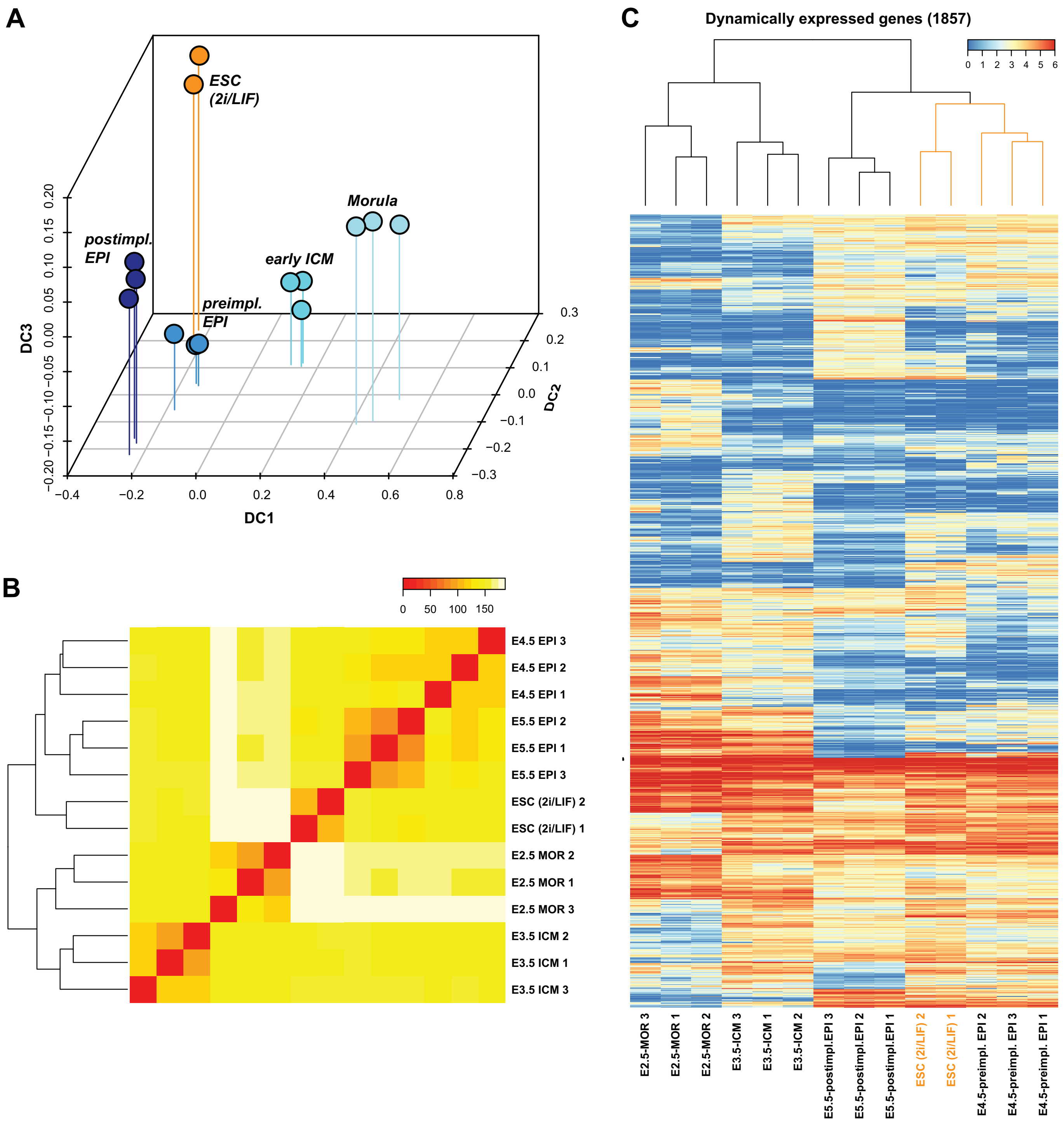

Figure S3, related to Figure 3: (A) Diffusion map of mouse embryonic samples and ESC cultured in 2i/LIF, based on the full transcriptome; DC = diffusion coefficient. (B) Global gene expression and hierarchical clustering of mouse embryonic samples and ESC in 2i/LIF. (C) Heatmap and hierarchical clustering based on the expression of dynamically regulated genes. The subgroup shared by ESC and E4.5 epiblast is marked in orange.

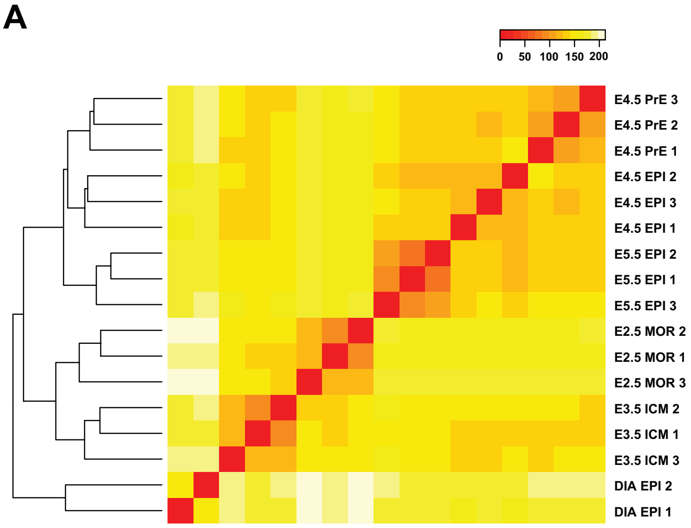

B

ESC(2i/LIF) / preimpl. EPI

DOWNREGULATED

GO enrichment					
Rank	Term	Count	ExpCount	Size	P-val.Adj (BH)
1.	cellular metabolic process	191	109	7688	4.4926E-17
2.	primary metabolic process	193	111	7865	4.9243E-17
3.	metabolic process	214	133	9440	4.8979E-16
4.	cellular process	255	188	13292	1.6293E-11
5.	cellular protein metabolic process	92	41	2908	2.0459E-11
6.	protein metabolic process	101	51	3574	6.8434E-10
7.	catabolic process	66	26	1845	6.8434E-10
8.	cellular macromolecule metabolic process	137	79	5619	6.8434E-10
9.	macromolecule metabolic process	149	91	6434	1.9745E-09
10.	cellular component organization or biogenesis	99	51	3578	2.905E-09
11.	cellular component organization	94	49	3447	2.4584E-08
12.	translation	32	8	598	3.0756E-08
13.	cellular catabolic process	55	22	1584	1.1601E-07
14.	cellular component biogenesis	50	21	1451	9.455E-07
15.	small molecule metabolic process	60	27	1937	1.1433E-06
16.	cell cycle	42	16	1132	2.0568E-06
17.	cellular component organization or biogenesis at cellular level	77	40	2851	2.1182E-06
18.	biosynthetic process	101	60	4235	3.8393E-06
19.	macromolecular complex subunit organization	35	13	944	3.1918E-05
20.	cellular biosynthetic process	95	58	4093	3.9861E-05
21.	organophosphate metabolic process	40	17	1183	4.1803E-05
22.	cellular component organization at cellular level	70	39	2731	6.6093E-05
23.	cellular component assembly	42	19	1312	8.0252E-05
24.	cell cycle process	30	11	776	8.2357E-05
25.	heterocycle metabolic process	37	16	1127	0.00020108

UPREGULATED

GO enrichment					
Rank	Term	Count	ExpCount	Size	P-val.Adj (BH)
1.	cellular biosynthetic process	31	12	4093	0.00028531
2.	biosynthetic process	31	13	4235	0.00028531
3.	cellular process	60	40	13292	0.00028531
4.	melanosome organization	3	0	11	0.0017611
5.	pigment granule organization	3	0	12	0.00187446
6.	positive regulation of aldosterone metabolic process	2	0	2	0.0021249
7.	positive regulation of aldosterone biosynthetic process	2	0	2	0.0021249
8.	cellular metabolic process	41	23	7688	0.00239135
9.	establishment of localization	24	10	3277	0.00317487
10.	regulation of cellular biosynthetic process	22	9	2903	0.0037436
11.	negative regulation of cellular biosynthetic process	12	3	959	0.0037436
12.	cellular pigmentation	3	0	20	0.0037436
13.	negative regulation of biosynthetic process	12	3	976	0.0037436
14.	regulation of biosynthetic process	22	9	2938	0.0037436
15.	cellular nitrogen compound metabolic process	29	14	4674	0.00391938
16.	positive regulation of transcription, DNA-dependent	12	3	998	0.00391938
17.	cellular component organization	24	10	3447	0.00391938
18.	nitrogen compound metabolic process	29	14	4716	0.00422368
19.	positive regulation of RNA metabolic process	12	3	1020	0.00422368
20.	nucleobase-containing compound metabolic process	28	13	4490	0.00433432
21.	negative regulation of cellular macromolecule biosynthetic process	11	3	893	0.00525252
22.	cellular component organization or biogenesis	24	11	3578	0.00525252
23.	cellular macromolecule biosynthetic process	23	10	3345	0.00525252
24.	negative regulation of nucleobase-containing compound metabolic p	11	3	903	0.00525252
25.	negative regulation of nitrogen compound metabolic process	11	3	916	0.00566842

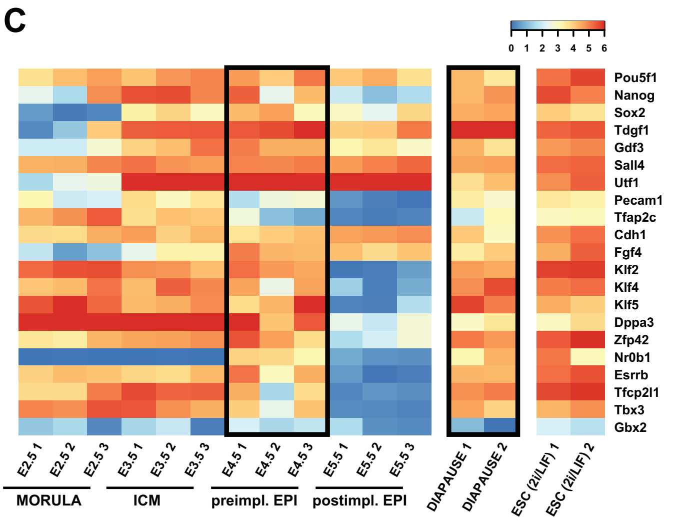

Figure S4, related to Figure 4: (A) Global expression and hierarchical clustering of mouse embryonic samples, ESC cultured in 2i/LIF and diapaused preimplantation epiblast. (B) GO term enrichment analysis based on up- and downregulated genes in normal preimplantation versus diapaused epiblast. (C) Expression of pluripotency markers in mouse embryonic samples, ESC in 2i/LIF and diapaused epiblast.

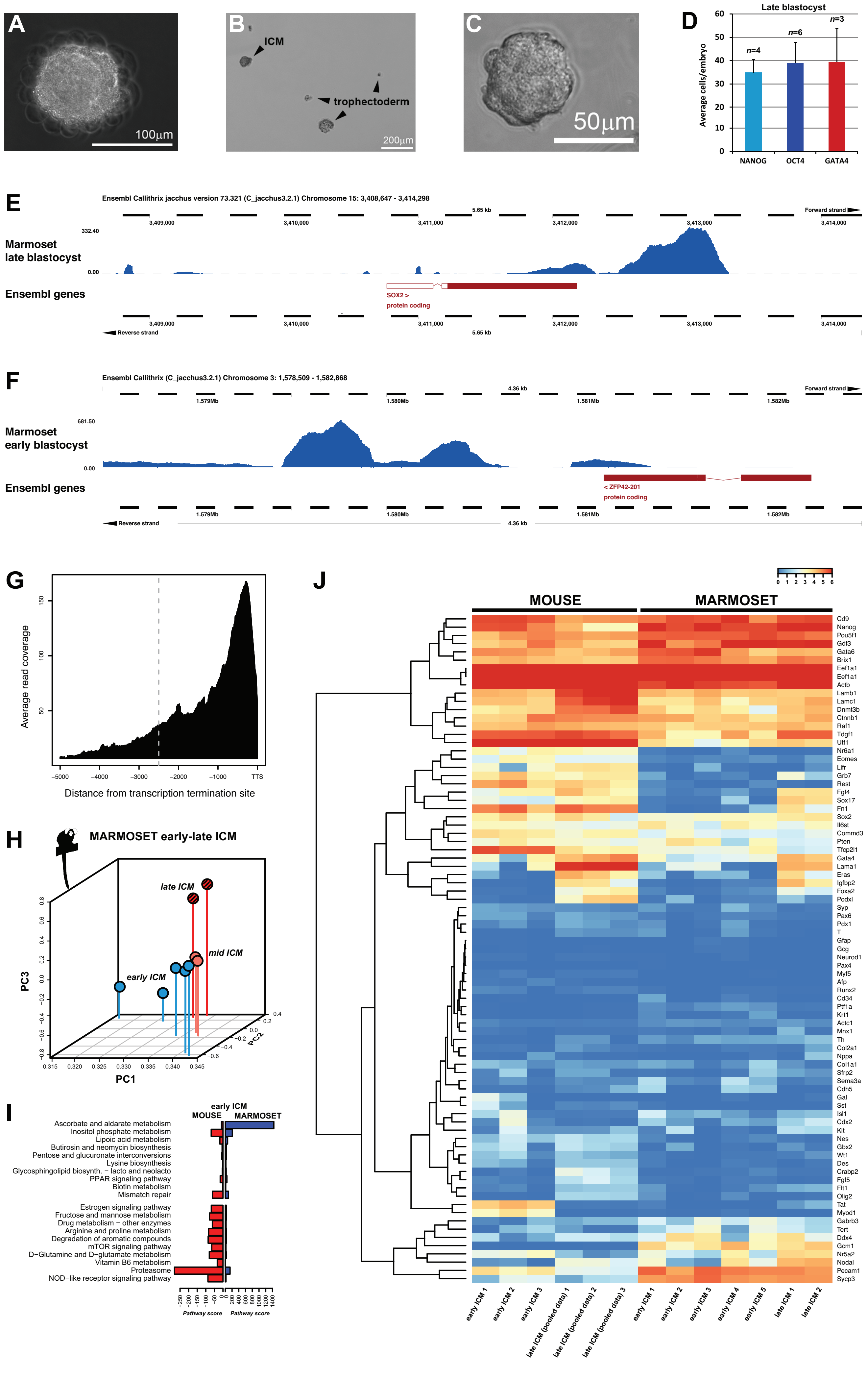

A
100µm
B
ICM
trophectoderm
200µm
C
50µm
D
Late blastocyst
Average cells/embryo
n=4
n=6
n=3
NANOG
OCT4
GATA4
E
Ensembl Callithrix jacchus version 73.321 (C_jacchus3.2.1) Chromosome 15: 3,408,647 - 3,414,298
5.65 kb
Forward strand
Marmoset
late blastocyst
332.40
0.00
Ensembl genes
SOX2 >
protein coding
Reverse strand
F
Ensembl Callithrix (C_jacchus3.2.1) Chromosome 3: 1,578,509 - 1,582,868
4.36 kb
Forward strand
1.579Mb
1.580Mb
1.581Mb
1.582Mb
Marmoset
early blastocyst
681.50
0.00
Ensembl genes
< ZFP42-201
protein coding
Reverse strand
G
Average read coverage
Distance from transcription termination site
TTS
H
MARMOSET early-late ICM
late ICM
mid ICM
early ICM
PC1
PC2
PC3
I
early ICM
MOUSE
MARMOSET
Ascorbate and aldarate metabolism
Inositol phosphate metabolism
Lipoic acid metabolism
Butirosin and neomycin biosynthesis
Pentose and glucuronate interconversions
Lysine biosynthesis
Glycosphingolipid biosynth. − lacto and neolacto
PPAR signaling pathway
Biotin metabolism
Mismatch repair
Estrogen signaling pathway
Fructose and mannose metabolism
Drug metabolism − other enzymes
Arginine and proline metabolism
Degradation of aromatic compounds
mTOR signaling pathway
D−Glutamine and D−glutamate metabolism
Vitamin B6 metabolism
Proteasome
NOD−like receptor signaling pathway
Pathway score
Pathway score
J
MOUSE
MARMOSET
Cd9
Nanog
Pou5f1
Gdf3
Gata6
Brix1
Eef1a1
Eef1a1
Actb
Lamb1
Lamc1
Dnmt3b
Ctnnb1
Raf1
Tdgf1
Utf1
Nr6a1
Eomes
Lifr
Grb7
Rest
Fgf4
Sox17
Fn1
Sox2
Il6st
Commd3
Pten
Tfcp2l1
Gata4
Lama1
Eras
Igfbp2
Foxa2
Podxl
Syp
Pax6
Pdx1
T
Gfap
Gcg
Neurod1
Pax4
Myf5
Afp
Runx2
Cd34
Ptf1a
Krt1
Actc1
Mnx1
Th
Col2a1
Nppa
Col1a1
Sfrp2
Sema3a
Cdh5
Gal
Sst
Isl1
Cdx2
Kit
Nes
Gbx2
Wt1
Des
Crabp2
Fgf5
Flt1
Olig2
Tat
Myod1
Gabrb3
Tert
Ddx4
Gcm1
Nr5a2
Nodal
Pecam1
Sycp3
early ICM 1
early ICM 2
early ICM 3
late ICM (pooled data) 1
late ICM (pooled data) 2
late ICM (pooled data) 3
early ICM 1
early ICM 2
early ICM 3
early ICM 4
early ICM 5
late ICM 1
late ICM 2

Figure S5, related to Figure 5: (A–C) Immunosurgery on marmoset blastocysts. Phase contrast images of (A) marmoset blastocyst after complement reaction, (B) roughly separated ICM and trophectoderm and (C) cleanly separated ICM after dissociation with a fine Pasteur pipette. (D) Manual quantification of NANOG, OCT4 and GATA4-positive cells from whole-mount immunostainings of in vivo derived, late marmoset blastocysts; n = number of embryos. (E) *SOX2* locus in the marmoset reference genome. Gene boundaries are annotated incorrectly with respect to transcribed sequence determined by RNA-seq. (F) A minority of genes such as *ZFP42* (*REX1*) were not accurately quantified due to pronounced discrepancies between marmoset gene loci and sequencing data that remained unreconciled by automated reannotation. (G) Read coverage for genes >3 kb illustrating 3′ bias and threshold implemented to restrict transcript length (dashed line). (H) Principal component analysis of marmoset embryonic samples. (I) Pathway expression scores for the 10 most up- and downregulated signaling pathways (KEGG). Scores were calculated by summing FPKM values of individual pathway components followed by normalization for pathway size. (J) Expression of pluripotency and lineage markers in mouse and marmoset early and late ICM samples. The mouse late ICM sample was generated by *in silico* pooling of RNA-seq reads from individual E4.5 EPI and E4.5 PrE samples at a ratio of 1:1. This reflects the ratio of epiblast to PrE cells in the late marmoset ICM (D) to allow comparison of late ICM in both species.

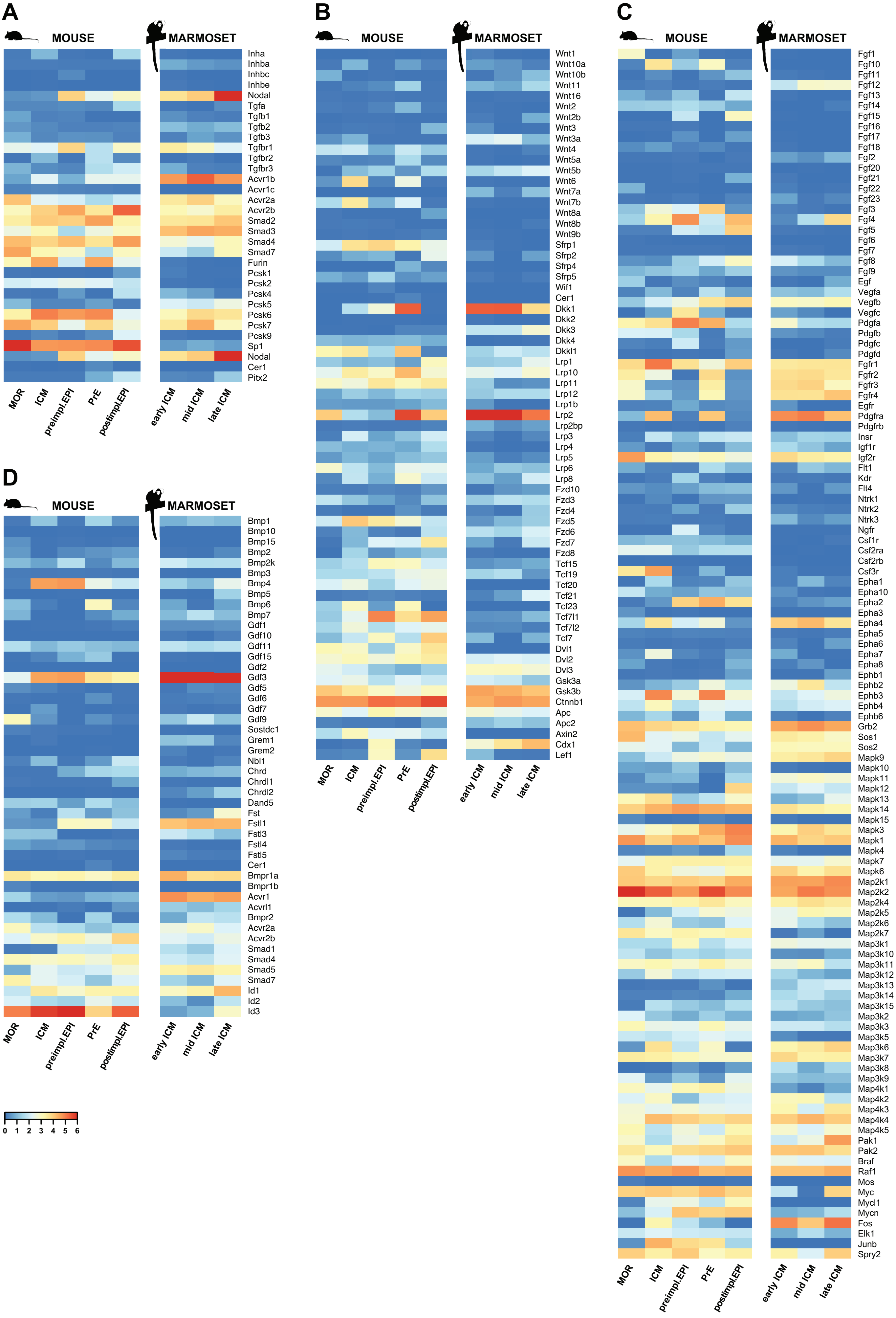

Figure S6, related to Figure 6: Expression data for (A) TGFß/NODAL, (B) WNT, (C) FGF and (D) BMP pathways.

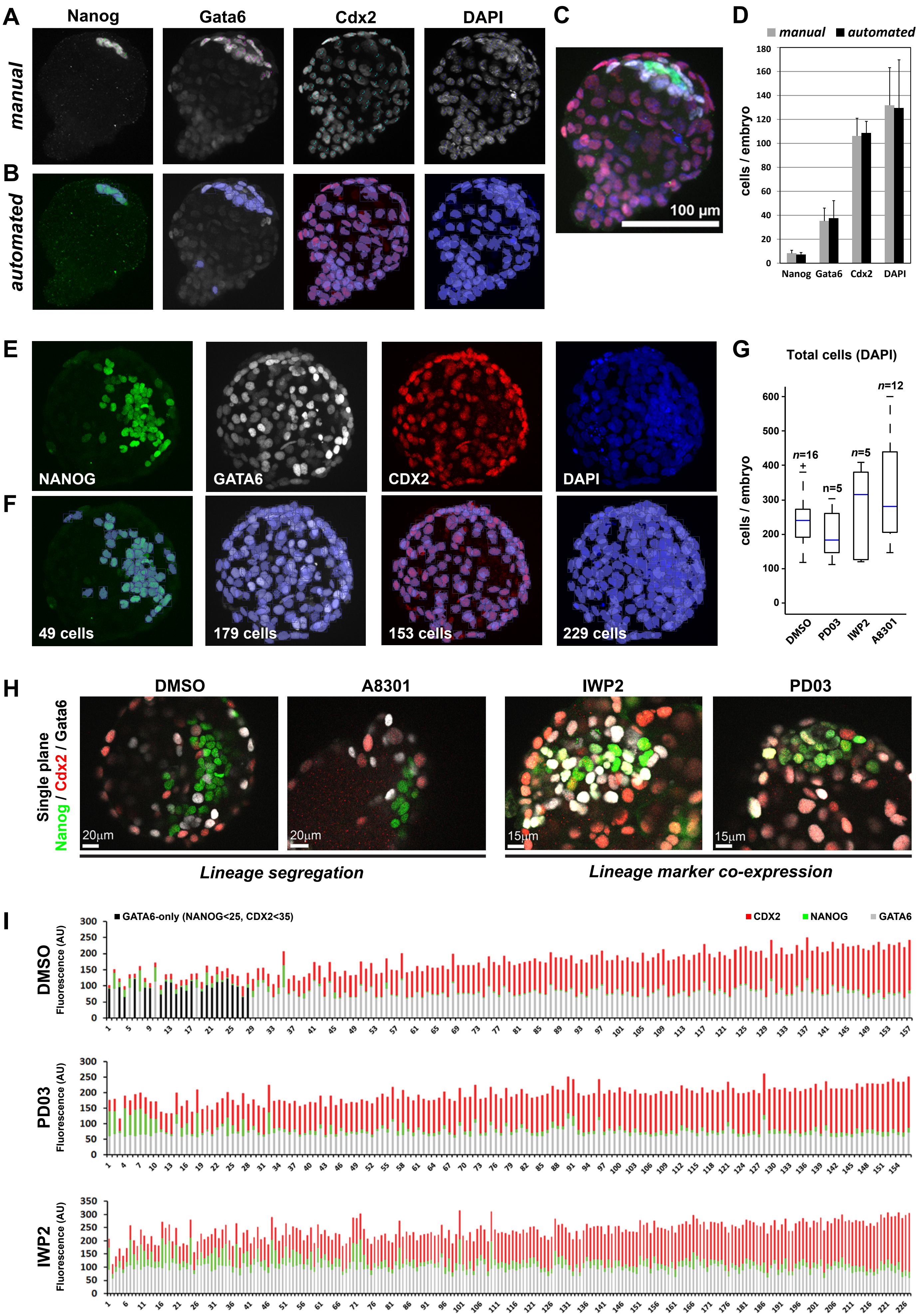

A
Nanog
Gata6
Cdx2
DAPI
manual
B
automated
C
100 µm
D
manual
automated
cells / embryo
Nanog
Gata6
Cdx2
DAPI
E
NANOG
GATA6
CDX2
DAPI
F
49 cells
179 cells
153 cells
229 cells
G
Total cells (DAPI)
cells / embryo
n=16
n=5
n=5
n=12
DMSO
PD03
IWP2
A8301
H
DMSO
A8301
IWP2
PD03
Single plane
Nanog / Cdx2 / Gata6
20µm
20µm
15µm
15µm
Lineage segregation
Lineage marker co-expression
I
GATA6-only (NANOG<25, CDX2<35)
CDX2
NANOG
GATA6
DMSO
Fluorescence (AU)
PD03
Fluorescence (AU)
IWP2
Fluorescence (AU)

Figure S7, related to Figure 6: (A–D) Comparison of manual and automated analysis of whole-mount embryo immunofluorescence confocal images. Late mouse blastocysts were stained for Nanog (epiblast), Gata6 (PrE), Cdx2 (trophectoderm) and DAPI (total cell number) and quantified by (B) manual counting of confocal *Z*-projections and (C) automated object quantification using Volocity. (D) Manual versus automated quantification; error bars = standard deviation. (E) Confocal *Z*-projection of a DMSO-treated (control) marmoset embryo stained for NANOG, GATA6, CDX2 and DAPI by immunocytochemistry. (F) Automated object quantification for the embryo shown in (E). The total number of cells identified is indicated in the lower left for each channel. (G) Quantification of total cell number (DAPI) from embryos cultured with and without inhibitors. Outliers are indicated with a cross. *P*-values were determined by one-way ANOVA with Tukey HSD testing. No significant differences were observed. (H) Single-plane confocal section of NANOG, CDX2 and GATA6 in inhibitor-treated marmoset embryos. (I) Fluorescence intensities of GATA6-positive cells in one representative embryo per experimental group. Bars represent individual cells. NANOG and CDX2 signal did not exceed background fluorescence in cells marked GATA6-only (black). For a cell to qualify as such, NANOG and CDX2 levels were no greater than background.

Table Legends

Table S1, related to Figure 1: Mouse gene expression levels for the developmental stages and samples analyzed in fragments per kilobase of exon per million fragments mapped (FPKM).

Table S2, related to Figure 2: Dynamically expressed genes in mouse embryonic lineages during development from the E2.5 morula stage to E5.5 postimplantation.

Table S3, related to Figure 2: Constitutively expressed genes in mouse development from morula to postimplantation stages.

Table S4, related to Figures 2 and 4: Mouse gene expression modules for the developmental stages and samples analyzed. Modules were derived by hierarchical clustering of scaled expression values.

Table S5, related to Figure 2: Genes differentially expressed between pairs of conditions in mouse embryonic development, diapaused epiblast and cultured ESC.

Table S6, related to Figure 5: Marmoset gene expression levels over the developmental stages analyzed in fragments per kilobase of exon per million fragments mapped (FPKM).

Table S7, related to Figure 5: Comparison of orthologous genes in mouse and marmoset ICM. Embryo-matched lineage-specific data from mouse epiblast and PrE samples were merged for compatibility with marmoset ICM.

Extended Experimental Procedures

Mouse strains

Embryos were generated by intercrosses between *Pdgfra::GFP* (Hamilton et al., 2003) and strain 129 estrus-checked females. Experiments were performed in accordance with EU guidelines for the care and use of laboratory animals, and under authority of UK governmental legislation. Use of animals in this project was approved by the ethical review committee for the University of Cambridge, and relevant Home Office licences are in place.

Mouse embryo staging and sample acquisition

Staging of mouse embryos was based on the assumption that, on average, mating occurred at midnight so that at midday embryos were assigned E0.5. Embryos were collected at the relevant stages from the oviduct or uterus in M2 medium (Sigma). Embryos were carefully matched by morphology and stage of development. An important aspect of this study was to profile freshly harvested, *in vivo* developed embryos. Consequently we avoided embryo culture and processed embryos as quickly as possible.

At the 8-cell stage embryos were flushed from the oviducts at 11.00am (just before E2.5). Embryos selected for precompaction with clearly separable eight individual cells. Zona pellucidae were removed with acidic Tyrode's solution (Sigma), cells were dissociated by brief exposure (<5min) to a 1:1 mixture of 0.025% trypsin plus EDTA (Invitrogen) and 0.025% trypsin (Invitrogen) plus 1% chick serum (Sigma). All individual samples (E2.5 MOR 1–3) contain 8 cells from one embryo.

Early blastocyst embryos represent the 32-cell stage harvested at 10.00am (prior to E3.5). For all early ICM samples blastocoel expansion was less than 50% of the total volume. Zona pellucidae were removed with acidic Tyrode's solution (Sigma), ICM were isolated by immunosurgery (Solter and Knowles, 1975, see below) and any residual trophectoderm was thoroughly removed by repetitive manual pipetting. ICM were dissociated by exposure (5–10min) to a 1:1 mixture of 0.025% trypsin plus EDTA (Invitrogen) and 0.025% trypsin (Invitrogen) plus 1% chick serum (Sigma). All cells were dissociated into singletons. Individual cell numbers of 12, 14 and 16 were processed immediately for RNA-seq.

Late blastocyst embryos were collected at E4.5 (11.00am). EPI and PrE have fully segregated at this stage (Plusa et al., 2008, Grabarak et al., 2012) and PrE cells can be visualized with the Pdgfra:GFP reporter which we used as an additional criterion to classify late blastocysts. Immunosurgery and single cell dissociation were carried out as described for early ICM with a slightly longer incubation time in dissociation buffer of 10–15min. Pulled glass capillaries of decreasing inner diameter were used to gently dissociate the ICM into single cells. We obtained 40–50 cells per embryo of which 10–20 remained in duplets or triplets. The vast majority of cells were either negative or strongly positive for Pdgfra-GFP. Single cells were sorted manually under a fluorescence dissection microscope based on GFP expression. Individual samples (e.g., E4.5 EPI) contained 10–20 single cells.

Postimplantation epiblast samples were isolated by manual dissection between 12.00–3.00 pm (just after E5.5). Embryos used had developed a cavity and the epiblast had formed an epithelium. Extraembryonic ectoderm was first removed with a wide mouth-controlled pipette, and the visceral endoderm was mechanically separated by drawing the embryo portion through a second pipette with a narrower aperture. Postimplantation epiblast samples were confirmed to be free of GFP-positive visceral endoderm. Epiblast dissociation was carried out as described for early ICM with a slightly longer incubation time of 10–15min. For individual samples (e.g., E5.5 EPI), 20 single cells were randomly selected and processed for RNA-seq.

Diapause induction

Embryonic diapause was induced by surgical removal of both ovaries from female mice on the morning of the third day of pregnancy (E2.5) without subsequent progesterone treatment. Diapaused embryos were flushed 4 days later and the epiblast was isolated in the same manner as for E4.5 embryos (see below).

Marmoset embryo collection

Marmoset blastocysts were staged based on embryonic day, diameter and blastocoel formation. Zona pellucidae were removed using acidic Tyrode's solution (Sigma) and embryos were subjected to immunosurgery as described below, using a custom rabbit polyclonal anti-marmoset antibody. Dissociation of marmoset ICM was carried out as for mouse embryos at the late blastocyst stage. Animals were obtained from self-sustaining colonies and housed according to standard husbandry guidelines. Protocols and institutional regulations for the care and experimental use of marmosets were strictly followed. Experiments at the DPZ were conducted under licence number AZ 33.42502–066/06. Experiments using marmosets at the CIEA were approved by the animal research committee (CIEA: 11028) and performed in compliance with guidelines set forth by the Science Council of Japan.

Immunosurgery

Mouse (E3.25 and E4.5) and marmoset blastocysts were processed by immunosurgery based on previously described procedures (Nichols et al., 1998; Solter and Knowles, 1975). Embryos were incubated for 15min in a 1:5 dilution of anti-mouse (Sigma) or anti-marmoset (in-house) rabbit serum in N2B27 medium, washed in N2B27 and incubated a further 15min in a 1:5 dilution of rat serum for the complement reaction. Embryos were transferred to N2B27 and incubated a further 15min for efficient cell lysis. ICM were dissociated from residual trophectoderm with a pulled Pasteur pipette of internal diameter just wider than the ICM.

Single cell dissociation

Dissociation of morula, ICM and postimplantation epiblast cells was performed in a 1:1 mixture of 0.025% trypsin plus EDTA (Invitrogen) and 0.025% trypsin (Invitrogen) plus 1% chick serum (Sigma). Morulae and early ICM were incubated for 5–10min at 37°C, and E4.5 mouse ICM and postimplantation epiblasts were incubated for 10–15min. Explants were subsequently washed in N2B27 supplemented with 10µM HEPES and 1.5mg/ml BSA (Gibco) and dissociated in a small drop of medium using blunted microcapillaries pulled to an inner diameter large enough to accommodate approximately 2–3 cells.

Embryonic stem cell culture

E14TG2a cells derived from mouse strain 129/Ola (Hooper et al., 1987) were used as a reference ESC line and cultured in 2i/LIF medium on gelatin (Ying et al., 2008). N2B27 (1:1 DMEM/F-12 and Neurobasal media, N2 (in-house) and B27 (Gibco) additives, 2mM L-glutamine and 100µM ß-mercaptoethanol) was supplemented with 1µM PD0325901, 3µM CHIR99021 and 10ng/ml LIF (in-house). Cells were dissociated by conventional methods in bulk culture. Single-cell suspensions were prepared and 20 cells were manually selected with a blunt microcapillary.

Immunofluorescence and cell counts

Embryos were stained as previously described (Nichols et al., 2009). Primary antibodies used were: Nanog (ReproCell RCAB0002P-F (1:300 dilution) or Cell Signaling Technology 4893 (1:400)), Gata4 (Santa Cruz sc1237 (1:100)), Oct4 (Santa Cruz sc8628 (1:300)), Gata6 (R&D AF1700 1:100)), Cdx2 (Biogenex MU392A 1:200)), Klf4 (Santa Cruz sc20691 (1:400)), Tfcp2l1 (R&D AF5726 (1:400)), Sox17 (R&D AF1924 (1:100)) and E-cadherin (Abcam ab15148 (1:200)). Cell counts from individual embryos were used for one-way ANOVA with Tukey HSD testing to identify significant changes between any two means (Figures 6C and 6D).

Sample processing and RNA-seq library construction

cDNA synthesis from small numbers of cells was carried out using whole-transcriptome preamplification (Tang et al., 2010b; Tang et al., 2009). Typically 10–20 cells (8 for morulae) were transferred immediately into 4.5μl single-cell lysis buffer under a dissection microscope and snap frozen on dry ice. cDNA was synthesized and amplified for 20 cycles using a single-temperature protocol in a Veriti 96-well thermocycler (Applied Biosystems). cDNA samples were sheared by ultrasonication on a Covaris S2 for 80s with parameter settings: Duty Cycle 10, Cycles per Burst 200, Intensity 4. Samples were ethanol precipitated and resuspended in Tris buffer for subsequent enzymatic modifications. End repair was carried out with T4 DNA polymerase and T4 polynucleotide kinase (New England Biolabs) and samples were purified with Ampure XP paramagnetic beads (Beckman Coulter). Blunt-end, 3′-phosphorylated products were 3′-adenylated with exo$^-$ Klenow fragment in the presence of dATPs (New England Biolabs), purified with Ampure beads, and ligated to sequencing adapters (Illumina and Bioo Scientific) by T4 DNA ligase at 20°C for 30 minutes. PCR amplification of library constructs was carried out with AccuPrime DNA polymerase (Invitrogen) for 13 cycles on a Bio-Rad C1000 thermocycler using denaturing and annealing conditions optimized for even A/T vs. G/C processing (Aird et al., 2011). Molarity and size distribution of sequencing libraries was assessed by DNA 1000 microfluidic chips on the 2100 Bioanalyzer (Agilent). Sequencing was performed in 100bp paired-end format on the Illumina HiSeq 2000 at high read depth (up to 250M per sample).

RNA-seq alignment and quantification

Remaining preamplification adapters were removed prior to alignment using AdapterRemoval (Lindgreen, 2012) at both 3′ and 5′ ends of reads. Sequencing reads were aligned to the GRCm38 assembly of the mouse genome (mm10) and the CalJac3.2.1 assembly of the common marmoset genome using GSNAP (Wu and Nacu, 2010), provided with splice junction annotation from Ensembl release 70 (Flicek et al., 2014). Only read pairs with unique mapping to the genome were considered further. Transcript quantification was performed with htseq-count, part of the Bioconductor *HTSeq* package (Anders et al., 2014), using gene annotation from Ensembl release 70. Samples were corrected for sequencing library size using the size factors computed by the *DESeq* package (Anders and Huber, 2010) and normalized for gene length to yield FPKM values. The cDNA preamplification protocol exhibits limited transcript processivity from the 3′ end (Tang et al., 2010a); we therefore restricted transcript lengths to 2500 bp for longer genes to mitigate this effect (Figure S5G). Global analyses, such as sample clustering and diffusion maps, were based on variance-stabilized counts computed by *DESeq*. In addition to the dataset described here, two previously published mouse E3.5 preimplantation epiblast samples produced as part of the same experiment and under identical conditions were analyzed in this study (ArrayExpress E-MTAB-2555, Boroviak et al., 2014). For comparison with published single-cell and bulk RNA-seq data, sequencing reads were obtained from the European Nucleotide Archive (Pakseresht et al., 2014) and processed as described above.

Marmoset gene reannotation

We observed that annotation of marmoset genes was often incorrect at the 3′ UTR (Figure S5E). As a result, documented 3′ end bias introduced by cDNA preamplification (Kurimoto et al., 2006, Tang et al., 2010b) led to underestimation of expression levels of affected genes. To address this, marmoset transcripts were assembled *de novo* from RNA-seq data using Trinity (Grabherr et al., 2011). Transcript contigs were then mapped to the marmoset reference genome and transcriptome sequences with BLAT (Kent, 2002). Newly-assembled transcripts that could be uniquely assigned to a known gene were incorporated into the genome annotation as a new isoform. To avoid false positives, regions of read coverage near annotated genes that could not be linked to the gene by an assembled transcript were not included in the reannotation (Figure S5F). This revised annotation was used to quantify the expression of marmoset genes and is provided with ArrayExpress record E-MTAB-2959.

Identification and clustering of dynamically regulated genes

Differential expression analysis was performed with *DESeq*. Since the expression of many genes changes over the developmental time course profiled, dispersions were estimated using fit type 'local'. Dynamically regulated genes for a set of embryonic stages were defined as genes that were 1) differentially expressed (FDR-corrected P-value < 0.05) between any two stages and 2) robustly transcribed (mean FPKM>10) in at least one. To identify clusters of genes that exhibit similar expression patterns, FPKM values were first scaled relative to the mean expression of the gene across all samples. Hierarchical clustering was then performed using the hclust algorithm in R. Clusters were extracted and manually classified into modules corresponding to the stage(s) of predominant expression. GO category and KEGG pathway enrichment analysis on differentially expressed and clustered genes was performed using the *GOstats* R package (Falcon and Gentleman, 2007). Genes were designated transcription factors if they were contained in a curated list of transcription factors (Vaquerizas et al., 2009) or were annotated with the GO term 'regulation of gene transcription (GO:0010468). Epigenetic regulators correspond to categories 'chromatin modification' and 'regulation of gene expression, epigenetic' (GO:0016568, GO:0040029), and signaling molecules to 'signaling' (GO:0023052).

Cross-species analysis

For comparative analyses between mouse and marmoset, orthologous genes were matched using data from Ensembl release 70 (Flicek et al., 2014). To obtain a comprehensive gene set, annotated one-to-one mouse-marmoset orthologs were supplemented with one-to-one marmoset-human orthologs corresponding to the mouse gene symbol. To render marmoset ICM samples comparable to the mouse data, EPI and PrE expression profiles from the same mouse embryos were pooled *in silico* at the observed marmoset ratio of 1:1 (Figure S5D). Expression data from mouse and marmoset orthologs are provided in Table S7.

Pathway analysis

Differential pathway analysis was performed for all 229 curated KEGG pathways. High-level comparative analyses between embryonic stages and species were based on pathway scores, defined as the sum of expression values of all components divided by the number comprising each pathway. For more detailed analyses, expression values were mapped onto pathway nodes centered around the mean expression of all genes (FPKM=24), with FPKM=0 and FPKM=100 as extremes, using PathVisio (Kutmon et al., 2015). For regulatory network analysis we produced a condensed version of PluriNetWork, curated based on published interactions (Som et al., 2010), by exclusively considering dynamically expressed genes. Dynamic expression of individual pathway components was determined by normalizing log-transformed expression values relative to the mean expression of the gene across all embryonic stages. A gene was deemed active at a given stage if relative expression was positive. Similarly, a network edge was considered active if both its source and target nodes were active. Cytoscape (Smoot et al., 2011) was used for network visualization.

Confocal imaging and analysis

Images were acquired with a Leica TCS SP5 confocal microscope. Optical section thickness ranged from 1–3µm. Images were processed using Leica software, Imaris, Volocity and ImageJ. Parameters for object identification were: guide size 500μm^3, separate objects 500 μm^3 and exclude objects larger than 500μm^3. Thresholds for background fluorescence (Gata6:45, Nanog:25, Cdx2:35, DAPI:20) were empirically determined to recapitulate manual cell counts in DMSO control embryos (Figures S7A–D). For two IWP2-treated embryos the threshold for Nanog was increased to 50 due to very bright signal to ensure accurate quantification of cell nuclei.

References

Aird, D., Ross, M.G., Chen, W.S., Danielsson, M., Fennell, T., Russ, C., Jaffe, D.B., Nusbaum, C., and Gnirke, A. (2011). Analyzing and minimizing PCR amplification bias in Illumina sequencing libraries. Genome Biol *12*, R18.

Anders, S., and Huber, W. (2010). Differential expression analysis for sequence count data. Genome Biol *11*, R106.

Anders, S., Pyl, P.T., and Huber, W. (2015). HTSeq—a Python framework to work with high-throughput sequencing data. Bioinformatics *31*, 166-9.

Boroviak, T., Loos, R., Bertone, P., Smith, A., and Nichols, J. (2014). The ability of inner-cell-mass cells to self-renew as embryonic stem cells is acquired following epiblast specification. Nat Cell Biol *16*, 516-528.

Chazaud, C., Yamanaka, Y., Pawson, T., and Rossant, J. (2006). Early lineage segregation between epiblast and primitive endoderm in mouse blastocysts through the Grb2-MAPK pathway. Dev Cell *10*, 615-624.

Falcon, S., and Gentleman, R. (2007). Using GOstats to test gene lists for GO term association. Bioinformatics *23*, 257-258.

Flicek, P., Amode, M.R., Barrell, D., Beal, K., Billis, K., Brent, S., Carvalho-Silva, D., Clapham, P., Coates, G., Fitzgerald, S.*, et al.* (2014). Ensembl 2014. Nucleic Acids Res *42*, D749-755.

Gerbe, F., Cox, B., Rossant, J., and Chazaud, C. (2008). Dynamic expression of Lrp2 pathway members reveals progressive epithelial differentiation of primitive endoderm in mouse blastocyst. Dev Biol *313*, 594-602.

Grabarek, J.B., Zyzyńska, K., Saiz, N., Piliszek, A., Frankenberg, S., Nichols, J., Hadjantonakis, A.K., and Plusa, B. (2012). Differential plasticity of epiblast and primitive endoderm precursors within the ICM of the early mouse embryo. Development *139*, 129-39.

Grabherr, M.G., Haas, B.J., Yassour, M., Levin, J.Z., Thompson, D.A., Amit, I., Adiconis, X., Fan, L., Raychowdhury, R., Zeng, Q.*, et al.* (2011). Full-length transcriptome assembly from RNA-Seq data without a reference genome. Nat Biotechnol *29*, 644-652.

Guo, G., Huss, M., Tong, G.Q., Wang, C., Li Sun, L., Clarke, N.D., and Robson, P. (2010). Resolution of cell fate decisions revealed by single-cell gene expression analysis from zygote to blastocyst. Dev Cell *18*, 675-685.

Hamilton, T.G., Klinghoffer, R.A., Corrin, P.D., and Soriano, P. (2003). Evolutionary divergence of platelet-derived growth factor alpha receptor signaling mechanisms. Mol Cell Biol *23*, 4013-4025.

Hooper, M., Hardy, K., Handyside, A., Hunter, S., and Monk, M. (1987). HPRT-deficient (Lesch-Nyhan) mouse embryos derived from germline colonization by cultured cells. Nature *326*, 292-295.

Kent, W.J. (2002). BLAT—the BLAST-like alignment tool. Genome Res *12*, 656-664.

Kutmon, M., van Iersel, M.P., Bohler, A., Kelder, T., Nunes, N., Pico, A.R., Evelo, C.T. (2015). PathVisio 3: an extendable pathway analysis toolbox. PLoS Comput Biol. *11*, e1004085.

Kurimoto, K., Yabuta, Y., Ohinata, Y., Ono, Y., Uno, K.D., Yamada, R.G., Ueda, H.R., and Saitou, M. (2006). An improved single-cell cDNA amplification method for efficient high-density oligonucleotide microarray analysis. Nucleic Acids Res *34*, e42.

Lindgreen, S. (2012). AdapterRemoval: easy cleaning of next-generation sequencing reads. BMC Res Notes *5*, 337.

Nichols, J., Silva, J., Roode, M., and Smith, A. (2009). Suppression of Erk signalling promotes ground state pluripotency in the mouse embryo. Development *136*, 3215-3222.

Nichols, J., Zevnik, B., Anastassiadis, K., Niwa, H., Klewe-Nebenius, D., Chambers, I., Scholer, H., and Smith, A. (1998). Formation of pluripotent stem cells in the mammalian embryo depends on the POU transcription factor Oct4. Cell *95*, 379-391.

Ohnishi, Y., Huber, W., Tsumura, A., Kang, M., Xenopoulos, P., Kurimoto, K., Oles, A.K., Arauzo-Bravo, M.J., Saitou, M., Hadjantonakis, A.K., et al. (2014). Cell-to-cell expression variability followed by signal reinforcement progressively segregates early mouse lineages. Nat Cell Biol *16*, 27-37.

Pakseresht, N., Alako, B., Amid, C., Cerdeno-Tarraga, A., Cleland, I., Gibson, R., Goodgame, N., Gur, T., Jang, M., Kay, S.*, et al.* (2014). Assembly information services in the European Nucleotide Archive. Nucleic Acids Res *42*, D38-43.

Plusa, B., Piliszek, A., Frankenberg, S., Artus, J., and Hadjantonakis, A.K. (2008). Distinct sequential cell behaviours direct primitive endoderm formation in the mouse blastocyst. Development *135*, 3081-3091.

Smoot, M.E., Ono, K., Ruscheinski, J., Wang, P.L., and Ideker, T. (2011). Cytoscape 2.8: new features for data integration and network visualization. Bioinformatics *27*, 431-432.

Solter, D., and Knowles, B.B. (1975). Immunosurgery of mouse blastocyst. Proc Natl Acad Sci USA *72*, 5099-5102.

Som, A., Harder, C., Greber, B., Siatkowski, M., Paudel, Y., Warsow, G., Cap, C., Scholer, H., and Fuellen, G. (2010). The PluriNetWork: an electronic representation of the network underlying pluripotency in mouse, and its applications. PLoS One *5*, e15165.

Tang, F., Barbacioru, C., Wang, Y., Nordman, E., Lee, C., Xu, N., Wang, X., Bodeau, J., Tuch, B.B., Siddiqui, A.*, et al.* (2009). mRNA-Seq whole-transcriptome analysis of a single cell. Nat Methods *6*, 377-382.

Tang, F., Barbacioru, C., Bao, S., Lee, C., Nordman, E., Wang, X., Lao, K., and Surani, M.A. (2010a). Tracing the derivation of embryonic stem cells from the inner cell mass by single-cell RNA-Seq analysis. Cell Stem Cell *6*, 468-478.

Tang, F., Barbacioru, C., Nordman, E., Li, B., Xu, N., Bashkirov, V.I., Lao, K., and Surani, M.A. (2010b). RNA-Seq analysis to capture the transcriptome landscape of a single cell. Nat Protoc *5*, 516-535.

Wu, T.D., Nacu, S. (2010) Fast and SNP-tolerant detection of complex variants and splicing in short reads. Bioinformatics *26*, 873-81.

Vaquerizas, J.M., Kummerfeld, S.K., Teichmann, S.A., and Luscombe, N.M. (2009). A census of human transcription factors: function, expression and evolution. Nat Rev Genet *10*, 252-263.

Ying, Q.L., Wray, J., Nichols, J., Batlle-Morera, L., Doble, B., Woodgett, J., Cohen, P., and Smith, A. (2008). The ground state of embryonic stem cell self-renewal. Nature *453*, 519-523.
