# Supplementary material for: Lineage-Specific Profiling Delineates the Emergence and Progression of Naive Pluripotency in Mammalian Embryogenesis
Source: Dev Cell. 2015 Nov 9;35(3):366–82. doi: 10.1016/j.devcel.2015.10.011 (PMC4643313; doi:10.1016/j.devcel.2015.10.011)
Supplement: Document S2. Article plus Supplemental Information [file mmc9.pdf]

Resource

Developmental Cell

Lineage-Specific Profiling Delineates the Emergence and Progression of Naive Pluripotency in Mammalian Embryogenesis

Highlights

- Transcriptome profiling establishes a reference map for early mouse development
- Lineage-specific RNA-seq captures the full dynamic range of gene expression
- ESCs and diapaused and preimplantation epiblasts share common pluripotency circuitry
- Primate early ICM utilizes additional signaling pathways for lineage specification

Authors

Thorsten Boroviak, Remco Loos, Patrick Lombard, ..., Jennifer Nichols, Austin Smith, Paul Bertone

Correspondence

austin.smith@cscr.cam.ac.uk (A.S.), bertone@ebi.ac.uk (P.B.)

In Brief

In this Resource article, Boroviak et al. perform lineage-specific transcriptome analysis of early mouse, diapaused, and marmoset embryos, defining regulatory modules mediating the establishment and loss of naive pluripotency. Cross-species comparisons reveal a conserved pluripotency transcriptional network but distinct signaling pathways for segregation of the inner cell mass.

CrossMark

Boroviak et al., 2015, Developmental Cell *35*, 366–382
November 9, 2015 ©2015 The Authors
http://dx.doi.org/10.1016/j.devcel.2015.10.011

CellPress

Lineage-Specific Profiling Delineates the Emergence and Progression of Naive Pluripotency in Mammalian Embryogenesis

Thorsten Boroviak,[1,10] Remco Loos,[2,10] Patrick Lombard,[1] Junko Okahara,[5] Rüdiger Behr,[3,4] Erika Sasaki,[5,6] Jennifer Nichols,[1,7] Austin Smith,[1,8,*] and Paul Bertone[1,2,9,*]
[1]Wellcome Trust–Medical Research Council Stem Cell Institute, University of Cambridge, Tennis Court Road, Cambridge CB2 1QR, UK
[2]European Bioinformatics Institute, European Molecular Biology Laboratory, Wellcome Trust Genome Campus, Cambridge CB10 1SD, UK
[3]Deutsches Primatenzentrum (German Primate Center), Leibniz-Institut für Primatenforschung, Kellnerweg 4, 37077 Göttingen, Germany
[4]DZHK (German Center for Cardiovascular Research), Wilhelmsplatz 1, 37073 Göttingen, Germany
[5]Department of Applied Developmental Biology, Central Institute for Experimental Animals, 3-25-12 Tonomachi, Kawasaki-ku, Kanagawa 210-0821, Japan
[6]Keio Advanced Research Center, Keio University, Shinjuku-ku, Tokyo 160-8582, Japan
[7]Department of Physiology, Development and Neuroscience, University of Cambridge, Tennis Court Road, Cambridge CB2 3EG, UK
[8]Department of Biochemistry, University of Cambridge, Tennis Court Road, Cambridge CB2 1GA, UK
[9]Genome Biology and Developmental Biology Units, European Molecular Biology Laboratory, Meyerhofstraße 1, 69117 Heidelberg, Germany
[10]Co-first author
*Correspondence: austin.smith@cscr.cam.ac.uk (A.S.), bertone@ebi.ac.uk (P.B.)
http://dx.doi.org/10.1016/j.devcel.2015.10.011
This is an open access article under the CC BY license (http://creativecommons.org/licenses/by/4.0/).

SUMMARY

Naive pluripotency is manifest in the preimplantation mammalian embryo. Here we determine transcriptome dynamics of mouse development from the eight-cell stage to postimplantation using lineage-specific RNA sequencing. This method combines high sensitivity and reporter-based fate assignment to acquire the full spectrum of gene expression from discrete embryonic cell types. We define expression modules indicative of developmental state and temporal regulatory patterns marking the establishment and dissolution of naive pluripotency in vivo. Analysis of embryonic stem cells and diapaused embryos reveals near-complete conservation of the core transcriptional circuitry operative in the preimplantation epiblast. Comparison to inner cell masses of marmoset primate blastocysts identifies a similar complement of pluripotency factors but use of alternative signaling pathways. Embryo culture experiments further indicate that marmoset embryos utilize WNT signaling during early lineage segregation, unlike rodents. These findings support a conserved transcription factor foundation for naive pluripotency while revealing species-specific regulatory features of lineage segregation.

INTRODUCTION

Pluripotency emerges in the mammalian epiblast during preimplantation blastocyst development. At the eight-cell stage, the embryo undergoes compaction and outer cells are directed toward the trophectoderm lineage. Interior cells become the inner cell mass (ICM) and subsequently diverge into pluripotent epiblast and extraembryonic primitive endoderm (PrE). In rodents, the preimplantation epiblast state can be captured in vitro as embryonic stem cells (ESCs) (Evans and Kaufman, 1981; Martin, 1981) and sustained indefinitely using defined media (Buehr et al., 2008; Li et al., 2008; Ying et al., 2008). The unrestricted potential of ESCs to generate all somatic tissues and the germline is termed "naive" pluripotency, and differs from other in vitro pluripotent states with respect to gene expression, signaling requirements, and epigenetic status (Brons et al., 2007; Leitch et al., 2013; Marks et al., 2012; Nichols and Smith, 2009; Tesar et al., 2007).

Individual epiblast cells rapidly lose the ability to give rise to self-renewing ESC colonies upon implantation (Boroviak et al., 2014), evidencing the transient nature of naive identity in normal development. The transition from the naive state is poorly characterized in vivo and in vitro. Upon implantation, epiblast cells acquire epithelial polarity (Bedzhov and Zernicka-Goetz, 2014) and lose naive pluripotency marker expression entirely by embryonic day (E)5.5 (Boroviak et al., 2014). However, the transcriptional network associated with this progression has been ill defined.

Pluripotency can be suspended in utero during diapause, a facultative condition of embryonic arrest (Mantalenakis and Ketchel, 1966; Mead, 1993; Renfree and Shaw, 2000). Embryonic diapause by delayed implantation has evolved to overcome conditions unfavorable for reproduction (Ketchel et al., 1966; Mantalenakis and Ketchel, 1966; Mead, 1993) and occurs in over 100 mammalian species (Renfree and Shaw, 2000). The distinction between transient, diapaused, and self-renewing pluripotent states remains elusive (Nichols and Smith, 2012).

Human and nonhuman primate pluripotent cells are maintained in vitro with culture regimes distinct from those used for mouse ESCs. They exhibit marked differences in gene

CrossMark

expression compared to primate preimplantation embryos (Nakatsuji and Suemori, 2002; Thomson et al., 1996, 1998; Yan et al., 2013). Propagation of nonhuman primate ESCs competent for chimera contribution has proven elusive (Kishi et al., 2014; Tachibana et al., 2012), although recent progress was reported for rhesus macaque using altered culture conditions (Fang et al., 2014). It is unclear why primate embryonic cells are more refractory to authentic ESC derivation compared to mouse. One contributing factor could be differences in signaling pathways driving lineage commitment and segregation in the ICM, which have not been defined in the primate.

Advances in sequencing protocols have enabled quantitative analysis of picogram amounts of RNA from individual cells and facilitated the study of early mammalian development at unprecedented resolution (Guo et al., 2010; Kurimoto et al., 2006; Tang et al., 2009). However, sequencing from minute quantities of starting material compromises the detection of low-abundance transcripts and impairs accurate estimation of expression levels (Brennecke et al., 2013; Grün et al., 2014). These limitations reduce the potential for comprehensive molecular characterization of individual cells. Here we show that this can be mitigated by applying single-cell sample preparation methods to small groups of cells. We adapted single-cell reverse-transcription and preamplification (Tang et al., 2009) for compatibility with Illumina sequencing and profiled clusters of 8–20 cells. This approach yields substantial enhancement in transcript detection and is suitable for experiments that call for accurate analysis of small cell numbers without a requirement to measure cell-to-cell variability.

We apply this technique to produce a transcriptional map of early mouse development from morula to postimplantation epiblast, and compare in vitro cultured ESCs and diapaused epiblasts in the context of this developmental sequence. We extend this analysis to the common marmoset (*Callithrix jacchus*), a small New World monkey presenting several practical advantages as a model for primate embryology. Profiling transcriptional activity in the inner cell mass of early-, mid-, and late-stage marmoset blastocysts allows direct comparison of rodent and primate development. These data reveal differences in regulatory timing and utilization of signaling pathways that we investigate through embryo culture experiments. Our results provide a framework for delineating the emergence and developmental progression of pluripotency in diverse mammals.

RESULTS

A Transcriptional Map of Early Mouse Development

We previously showed by qRT-PCR that a moderate increase in starting material for whole-transcriptome amplification (Tang et al., 2009) substantially increases signal fidelity and reproducibility (Boroviak et al., 2014). We extend this approach to RNA sequencing (RNA-seq) to profile cell lineages from individual mouse embryos. Four time points were analyzed from the eight-cell stage at E2.5 to the early postimplantation epiblast at E5.5 (Figure 1A; Table S1). *Pdgfra::GFP* knockin mice (Hamilton et al., 2003; Plusa et al., 2008) enabled fluorescence-based separation of PrE from epiblast cells in E4.5 and E5.5 blastocysts.

We assessed transcript detection and expression-level estimation relative to previously published single-cell (Xue et al., 2013; Yan et al., 2013) and conventional RNA-seq data (Chan et al., 2013; Marks et al., 2012). Transcription was measured from up to 30% of annotated genes by single-cell RNA-seq, consistent with previous reports (Brennecke et al., 2013; Grün et al., 2014). RNA-seq from 10–20 cells (8 in the case of E2.5 morulae) yielded detection rates of 60%–70%, comparable to the performance of sequencing protocols from microgram quantities of RNA (Figure 1B). Similar distribution profiles were observed from bulk RNA and small numbers of cells, with many genes expressed at low and intermediate levels and a small proportion showing high expression (Figure 1C). In contrast, single-cell data exhibit high expression-level estimates for many genes and missing values for low-abundance transcripts (Kharchenko et al., 2014). These results demonstrate that profiling small cell clusters overcomes limitations in sensitivity of single-cell analysis and allows quantification of gene expression levels comparable to that of conventional transcriptome sequencing.

Analysis of biological replicates spanning the five embryonic stages produced discrete clusters, recapitulating their developmental sequence (Figure S1A). Visualization by diffusion map, a nonlinear dimensionality reduction method (Lafon et al., 2006), shows that samples cluster primarily by stage, with the first coefficient capturing progression of development (Figure 1D). PrE and preimplantation epiblast cells retain a high degree of similarity despite the divergent developmental potential of the two lineages (Figure 1D; Figure S1A).

We examined pluripotency and lineage markers in more detail. Our data confirm the sequential activation of PrE specifiers previously described (Figure S1B) (Artus et al., 2011; Chazaud et al., 2006; Gerbe et al., 2008; Guo et al., 2010; Kurimoto et al., 2006; Ohnishi et al., 2014), whereas preimplantation epiblast cells exhibited robust expression of exclusive markers including *Sox2*, *Nanog*, *Klf2*, *Bmp4*, and *Fgf4* (Guo et al., 2010; Kurimoto et al., 2006; Tang et al., 2010a). Strikingly, however, several transcription factors associated with pluripotency were expressed in both lineages. *Pou5f1* (*Oct4*) was expressed at lower levels but was still present in PrE, consistent with known protein distribution (Frum et al., 2013; Le Bin et al., 2014; Palmieri et al., 1994). This pattern was evident for *Klf4*, *Dppa3*, *Nr0b1*, *Esrrb*, and *Zfp42* (*Rex1*). *Klf5* and *Tbx3* were expressed at higher levels in PrE (Figure 1E). This observation is reflected in the cluster proximity of PrE and the preimplantation epiblast (Figure 1D). These results illustrate that divergent potency of related embryonic lineages can be conferred by a small subset of regulatory factors in the context of a globally similar transcriptome.

Defining Stage-Specific Gene Expression Modules in Early Mouse Development

Lineage-specific profiling facilitated identification of gene sets indicative of developmental state, lineage segregation, and progression. We defined dynamically expressed genes as those transcribed differentially between at least two of the four embryonic stages analyzed and robustly detected in at least one (fragments per kilobase of exon per million fragments mapped [FPKM] $\geq$ 10). The resultant set comprises 1,857 genes (Table S2), and hierarchical clustering revealed ten expression modules (Figure 2A; Figure S2A).

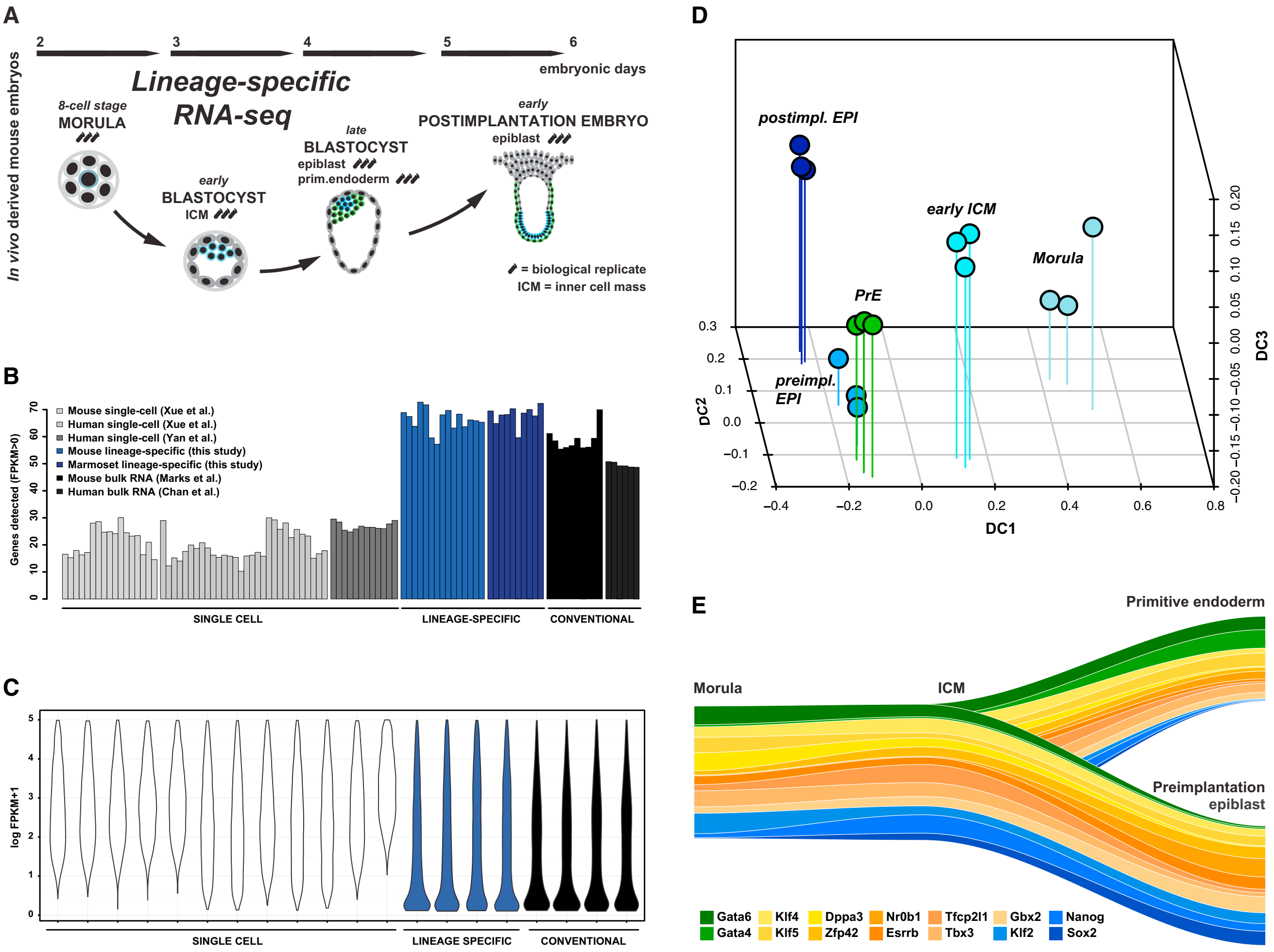

Figure 1. Transcriptome Profiling of Mouse Embryonic Lineages

(A) Overview of the developmental sequence analyzed.

(B) Percentage of detected genes in RNA-seq data from single cells (white), small numbers of cells (blue), and conventional bulk RNA (black) on comparable cell types (Xue et al., 2013; Yan et al., 2013, Marks et al., 2012).

(C) Distribution of nonzero expression values in $\log_2$ FPKM (fragments per kilobase of exon per million fragments mapped) for RNA-seq data from single cells (white), small numbers of cells (blue), and conventional bulk RNA (black).

(D) Diffusion map of embryonic samples from morula to postimplantation epiblast; DC, diffusion coefficient.

(E) Marker expression delineates the divergence of epiblast and PrE lineages. Genes specific to PrE and the preimplantation epiblast are marked in green and blue, respectively; shared genes are depicted in orange. Track width is scaled to relative expression normalized to the mean across all stages displayed.

Genes maximally expressed in morulae included *Tead4*, *Sox15*, *Klf17*, *Nr5a2*, and *Tfap2c*. Those common to morulae and the ICM were *Gata6*, as well as trophectodermal markers *Hand1*, *Elf3*, and *Eomes*. The latter group may underlie the capacity of early ICM cells to regenerate trophectoderm (Handyside, 1978; Nichols and Gardner, 1984; Rossant and Lis, 1979). Genes expressed throughout preimplantation development but at varying levels included ESC markers *Klf2*, *Klf4*, *Klf5*, *Nr0b1*, *Zfp42*, *Prdm14*, *Tbx3*, *Tfcp2l1*, and *Esrrb*. These data provide a comprehensive set of transcription factors and other genes downregulated upon implantation (Table S2).

In addition to *Pou5f1* (*Oct4*) and *Sall4*, pluripotency markers associated with both pre- and postimplantation epiblast include *Utf1*, *Foxd3*, *Zic3*, and *Fgf4*. *Tcf7l1* (*Tcf3*), a gene expressed in embryonic stem cells and involved in pluripotency repression (Martello et al., 2012; Wray et al., 2011; Yi et al., 2011), was upregulated at E4.5, potentially to prepare naive epiblast cells for transition. Notably, 220 genes were specifically upregulated during the preimplantation-to-postimplantation epiblast transition. These include known regulators of early postimplantation development, such as *Pou3f1* (*Oct6*), *Fgf5*, *Otx2*, and *Sox3*, as well as candidate regulators *Tead2*, *Sall2*, *Mapk12*, *Fzd2*, *Rspo1*, *Smo*, and *Notch3*. We also observed strong upregulation of de novo methyltransferases *Dnmt3a* and *Dnmt3b*, consistent with hypomethylation in naive pluripotent cells (Leitch et al., 2013; Smith et al., 2012) and increased DNA methylation upon implantation (Lee et al., 2014).

Genes expressed at high levels throughout the developmental stages analyzed are listed in Table S3, and expression modules specific to the epiblast versus PrE segregation process are

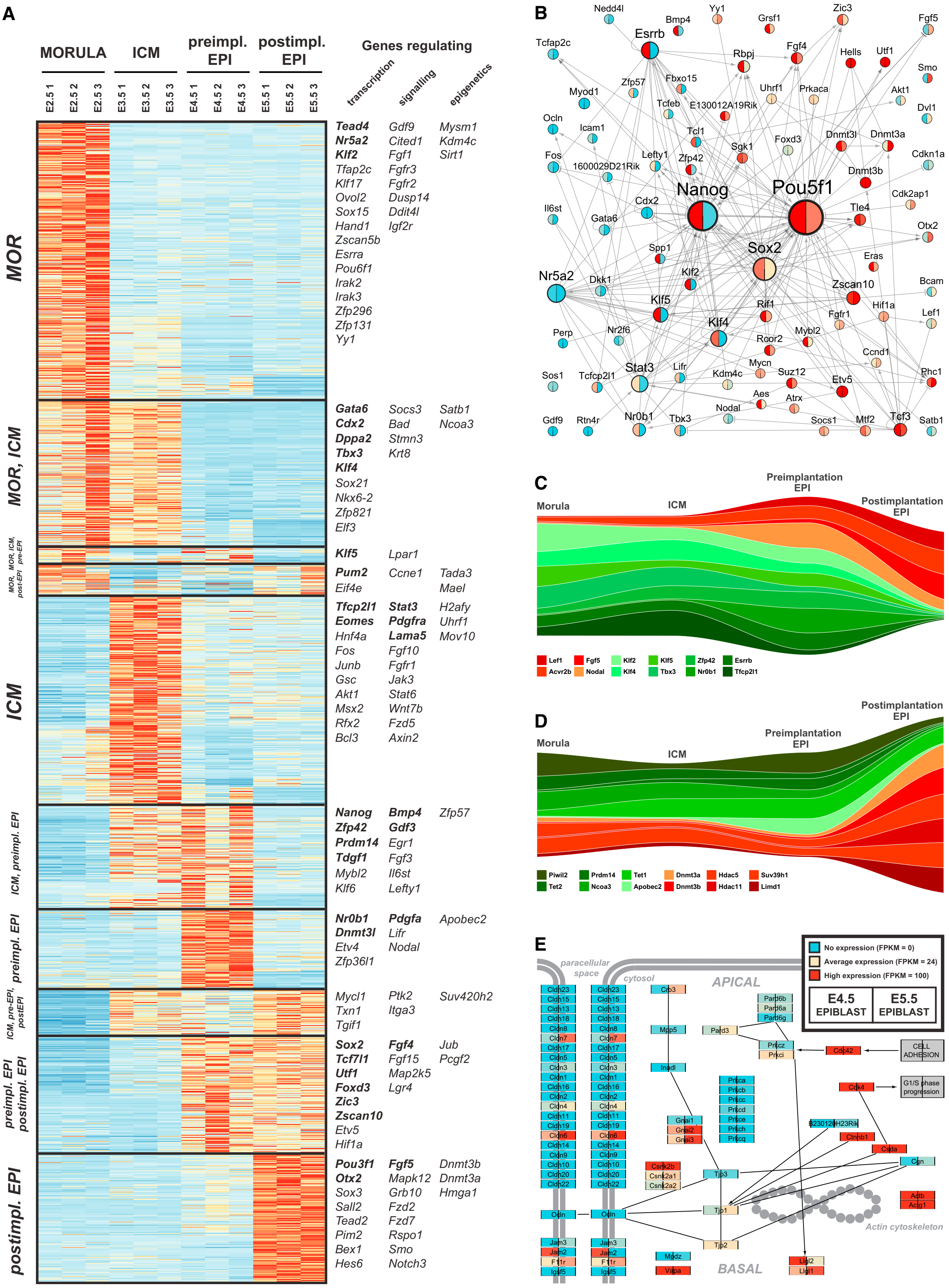

(legend on next page)

provided in Figure S2B and Table S4. These results expand the recent description of PrE-associated genes by single-cell microarray profiling (Ohnishi et al., 2014) and provide a resource to identify regulators of PrE differentiation.

The initial progression from naive pluripotency in embryonic development has not been well characterized. We analyzed the E4.5-to-E5.5 transition before epiblast cells undergo lineage priming in vivo and provide the complete set of differentially expressed genes in Table S5. To examine the behavior of an independent set of pluripotency-associated genes, we used PluriNetWork, curated from published interaction data (Som et al., 2010). Focusing on dynamically expressed genes and the core regulators *Pou5f1*, *Nanog*, and *Sox2*, we reduced the network to 82 nodes (PluriNet82; Figure 2B). Importantly, the condensed network includes all validated regulators of ESC pluripotency. Those downregulated during the E4.5-to-E5.5 transition include naive markers Esrrb, Nr0b1, Klf2, Klf4, Klf5 and Zfp42 (Rex1), as well as Lifr, Il6st (gp130), Spp1, Tcl1, and Zfp57. Conversely, we observed upregulation of *Foxd3*, *Lef1*, *Ccnd1*, *Zscan10*, *Phc1*, and *Nr216* upon implantation.

Visualization of temporal patterns shows robust expression of naive markers during preimplantation development followed by an abrupt shutdown and replacement with factors such as *Nodal*, *Lef1*, and *Fgf5*, along with the activin receptor *Acvr2b* (Figure 2C). Epigenetic modifiers associated with a permissive chromatin state (*Tet1*, *Tet2*, *Prdm14*, *Ncoa3*) were predominant during preimplantation development (Figure 2D). Upon implantation, these were exchanged for epigenetic regulators mediating DNA methylation (*Dnmt1*, *Dnmt3a*, *Dnm3b*) and transition to more closed chromatin configurations (*Hdac5*, *Hdac11*, *Suv39h1*).

We integrated the RNA-seq data with 229 annotated Kyoto Encyclopedia of Genes and Genomes (KEGG) pathways and generated expression maps for pairwise comparison of developmental stages (http://pathway-atlas.stemcells.cam.ac.uk). At the preimplantation-to-postimplantation epiblast transition, we noted differential expression of genes involved in tight junction formation (Figure 1E). In particular, we detected upregulation of *Cldn6*, important for epithelial formation (Turksen and Troy, 2001) and upregulated in mouse epiblast-derived stem cells (Tesar et al., 2007), and *Cldn7* with *Tjp1*, essential for tight junction establishment (Matter and Balda, 1999; Sleeman and Thiery, 2011). We also noted induction of *Crb3* and *Pard3*, pivotal for the establishment and maintenance of apical-basal polarity (Shin et al., 2006). These results comprise a reference dataset for regulatory network and pathway analysis during epiblast progression in vivo.

The Transcriptional Circuitry for ESC Identity Is Assembled in the Preimplantation Epiblast

We sequenced RNA from ESCs cultured in 2i/LIF (leukemia inhibitory factor) without feeders or serum (Ying et al., 2008), processed identically to embryonic samples to allow direct comparison. Global analysis showed tight correlation between biological replicates and confirmed the shared identity of ESCs and the preimplantation epiblast (Figures S3A and S3B). Restricting the comparison to dynamically expressed genes reveals concordance in the developmental state, with ESCs displaying a near-identical profile to the E4.5 epiblast (Figure 3A; Figure S3C). Consistent with this, genes differentially expressed between ESCs and embryonic cells were fewest for the E4.5 epiblast, indicating the greatest correspondence with emergent pluripotent cells in the blastocyst (Figure 3B). Pathway enrichment analyses showed most changes in ESCs to be associated with metabolism, potentially rooted in the biophysical environment and nutrient utilization (Figure 3C).

We examined the behavior of regulatory networks governing pluripotency in ESCs in the context of in vivo development. To capture changes over time, we computed normalized expression relative to the mean level of each gene across all developmental stages and defined genes with positive values as preferentially active at a given stage. Mapping these values to PluriNet82 reveals changes in network topology during early mouse development (Figure 3D). Few pluripotency genes exhibited robust expression at the morula stage, with substantially more upregulated in the early ICM. Most peaked at the preimplantation epiblast stage, resulting in maximum interconnectivity. This was abolished following implantation, marking the dissolution of naive pluripotency in vivo.

We proposed a reduced set of abstract Boolean network models for ESC self-renewal comprising 11 transcription factors and the extracellular signal-regulated kinase (Erk)/mitogen-activated protein (MAP) kinase (Dunn et al., 2014). To relate this to the embryo, we asked whether a developmental stage can be identified at which all components are present. Intersecting data from morula, early ICM, and pre- and postimplantation epiblast cells revealed that the only time point at which these regulators were coexpressed was the preimplantation epiblast (Figure 3E). Thus, transcriptional regulation in cultured ESCs correlates specifically with the naive phase of pluripotency in the embryo.

Diapaused Epiblasts Maintain All Features of Naive Pluripotency

Rodents have evolved the capacity for facultative developmental arrest at the late blastocyst stage (Mantalenakis and Ketchel,

Figure 2. Expression Modules Identified in Early Mouse Development and the Preimplantation-to-Postimplantation Epiblast Transition

(A) Expression of dynamically expressed genes. Modules were derived by hierarchical clustering of scaled expression values. Selected transcription factors, signaling pathway components, and epigenetic regulators are shown with pluripotency-associated genes in bold.

(B) PluriNet82 at the transition from pre- to postimplantation stages. Label and node sizes reflect interaction number. Colors represent expression in FPKM for preimplantation (E4.5; left) and postimplantation epiblasts (E5.5; right). Arrows indicate positive interactions; T bars indicate inhibitions.

(C) Genes characteristic of preimplantation (green) and early postimplantation development (red). Track width is scaled to relative expression normalized to the mean across all stages displayed.

(D) Epigenetic modifiers expressed predominantly at preimplantation (green) or early postimplantation (red) stages.

(E) Simplified representation of the KEGG "tight junction" pathway, with nodes colored according to expression in the preimplantation (left) and postimplantation epiblast (right).

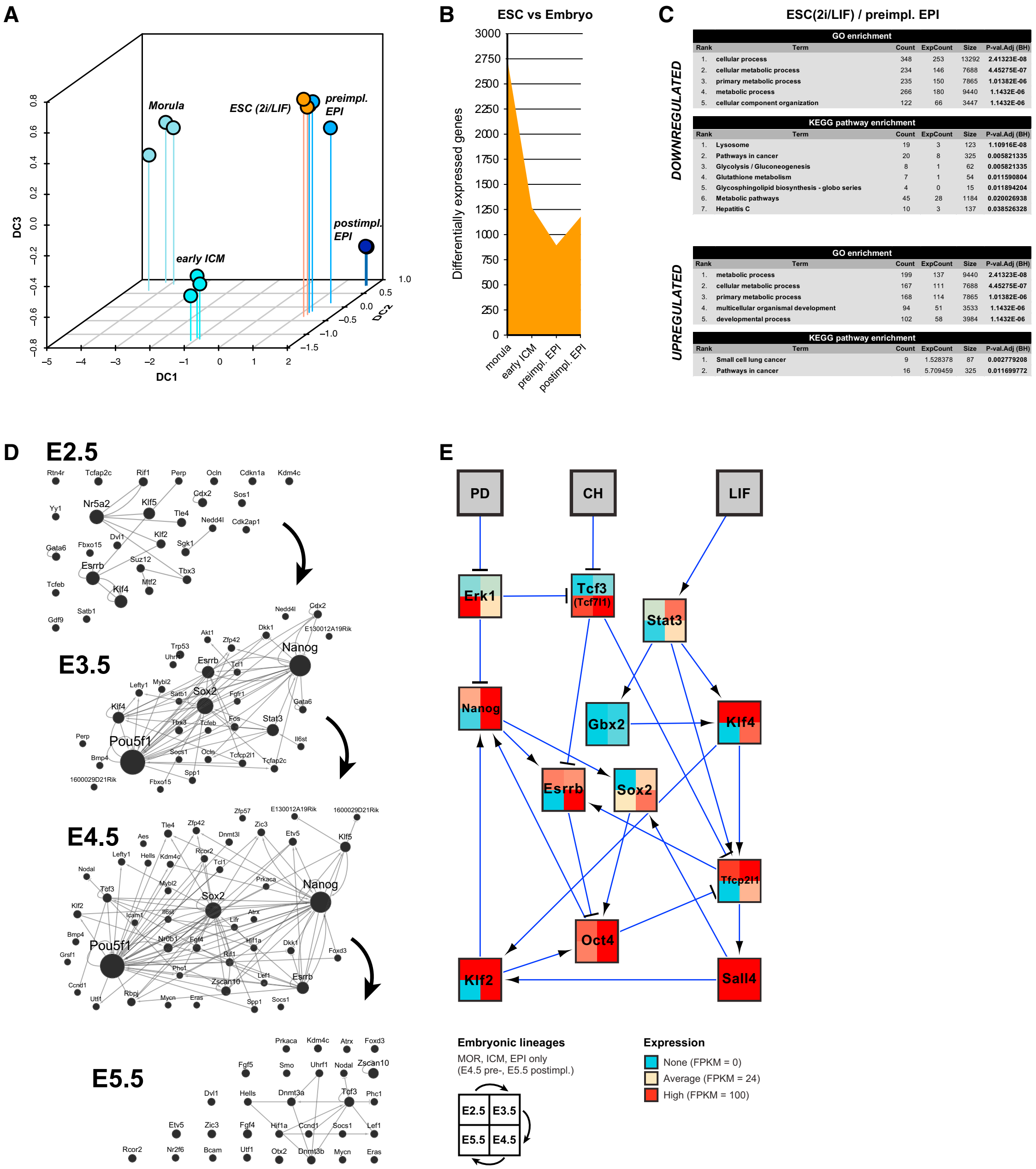

Figure 3. ESCs Retain Expression Modules Defining the Preimplantation Epiblast

(A) Diffusion map from morula to postimplantation epiblast and ESC cultured in 2i/LIF, based on dynamically expressed genes.

(B) Genes differentially expressed ($p < 0.05$) between ESCs and embryonic samples.

(C) Most significantly enriched GO and KEGG pathways based on up- and downregulated genes in ESC versus preimplantation epiblast.

(D) Changes in expression of PluriNet82 genes in the embryonic lineage. A node is displayed if the corresponding gene is predominantly active at that developmental stage, defined as positive log-transformed expression relative to the mean across all stages. An edge is displayed if both source and target nodes are active.

(E) Minimal set of transcription factors operative in mouse ESCs (Dunn et al., 2014). Colors represent gene expression in FPKM. Expression levels are depicted for morula, early ICM, and pre- and postimplantation epiblasts clockwise from the top left.

1966; Mead, 1993; Renfree and Shaw, 2000). ESCs were first obtained from embryos in diapause (Evans and Kaufman, 1981), and the condition is known to facilitate ESC derivation (Brook and Gardner, 1997). We induced implantation delay by ovariectomy (Weitlauf and Greenwald, 1968) and isolated diapaused epiblasts for RNA-seq as above. Diapaused embryos vary from those undergoing normal development (Figure 4A) and cluster separately by correlation analysis (Figure S4A). To characterize these differences, we examined Gene Ontology (GO) term enrichment for differentially expressed genes (Figure S4B). In

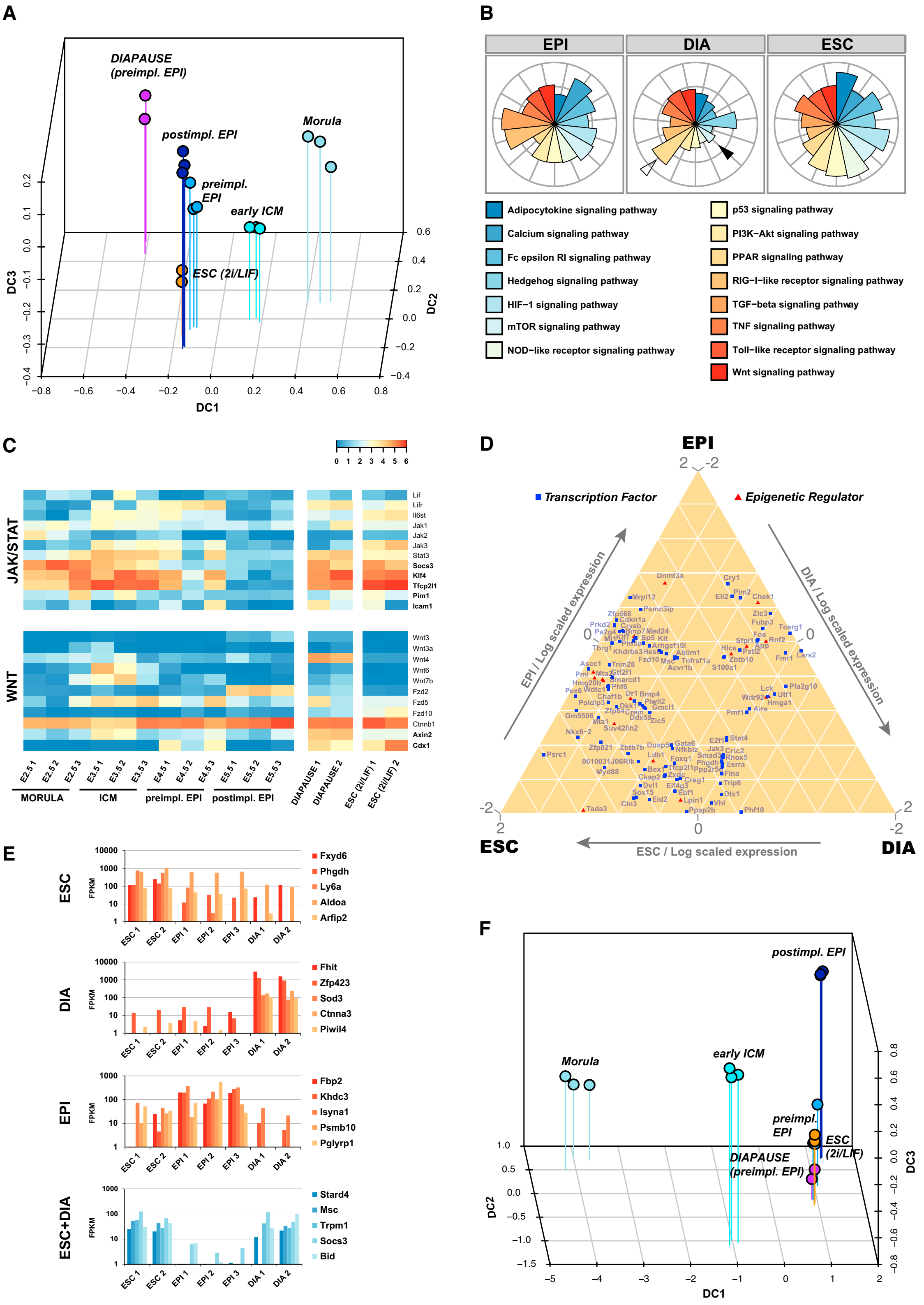

Figure 4. Relationship between Diapaused Epiblast, Normal Embryonic Development, and ESC

(A) Diffusion map of developmental stages from morula to postimplantation epiblast, ESC, and diapaused epiblast.

(B) Expression scores for selected signaling pathways, scaled to the mean across the three cell types, calculated by summing FPKM values of individual components followed by normalization for pathway size.

(legend continued on next page)

diapause, the most significant downregulated processes relate to metabolism, cell cycle, and biosynthesis. Conversely, those upregulated include negative regulation of metabolism and biosynthetic processes. Apart from peroxisome proliferator-activated receptor (PPAR) signaling, pathway expression scores were generally reduced in diapaused epiblasts and particularly components of the mechanistic target of rapamycin (mTOR) pathway (Figure 4B). These features likely reflect a general state of dormancy in diapaused embryos.

We found all pluripotency factors to be expressed in the diapaused epiblast, indicating retention of naive identity (Figure S4C). It has been shown that *Lifr* and *Il6st* serve an essential function in epiblast maintenance during diapause (Nichols et al., 2001), implicating the Janus kinase (JAK)/signal transducer and activator of transcription (STAT) pathway in sustaining pluripotency. Analysis of JAK/STAT components revealed that receptors, signal transducers, and downstream targets are robustly expressed in normal preimplantation development, ESCs, and diapaused epiblasts but are not maintained postimplantation (Figure 4C). Pluripotency factor targets of Stat3, Klf4, and Tfcp2l1 (Bourillot et al., 2009; Hall et al., 2009; Martello et al., 2013; Niwa et al., 2009; Ye et al., 2013) were expressed at high levels in diapause. We also observed substantial upregulation of *Wnt4*, concomitant with high expression of the WNT targets *Esrrb* (Martello et al., 2012), *Axin2* (Lustig et al., 2002), and *Cdx1* (Pilon et al., 2007) (Figure 4C; Figure S4C). *Wnt* expression in normal development was mainly confined to the early ICM (*Wnt6*, *Wnt7b*), with low levels of *Wnt4* present in all stages analyzed. Thus, WNT signaling may play a specific role in extended maintenance of the pluripotency network during diapause.

We assessed genome-wide differences in expression of transcription factors and epigenetic regulators between the preimplantation epiblast, diapause, and ESC (Figure 4D). Few factors were highly expressed in only one condition and most were present in at least two, in particular ESC and preimplantation epiblast or ESC and diapause. Expression modules including all genes for preimplantation epiblast, diapause, and ESC appear in Table S4. ESC-specific transcripts include *Phgdh* and *Aldoa*, involved in glycolysis and L-serine biosynthesis, respectively (Figure 4E). *Piwil4*, a repressor of transposable elements, and *Sod3*, a superoxide dismutase, may act to maintain genomic integrity during diapause. Genes highly upregulated in both diapause and ESC included *Socs3*, a JAK/STAT downstream target, and *Stard4*, encoding a putative lipid transfer protein. We examined the relationship of diapaused epiblasts to embryo samples and ESCs based on all 1,857 genes dynamically expressed during normal development. Remarkably, diapause samples were placed directly adjacent to the preimplantation epiblast and ESC in the diffusion map (Figure 4F). These results show self-renewing ESCs and diapaused embryos to embody an arrested state of the transient E4.5 epiblast that retains developmental identity, despite substantial changes in environment in the case of ESCs and profound changes in metabolism, proliferation, and biosynthetic activity in diapause.

Direct Comparison of Rodent and Primate ICM

Preimplantation embryogenesis in primates is protracted relative to rodents, and detailed molecular and functional characterizations are lacking. Protocols for minimally invasive embryo recovery have been developed for marmoset, providing access to embryos that have undergone normal gestation in utero (Thomson et al., 1994; Hanazawa et al., 2012), a resource that is not accessible from humans. We therefore utilized the common marmoset to investigate the transcriptional makeup of primate ICM in comparison to early mouse development.

Marmoset embryos were collected by nonsurgical uterine flush. The ICMs of early-, mid-, and late-stage blastocysts were isolated by immunosurgery (Figures S5A–S5C) and profiled by RNA-seq (Table S6). Marmoset ICMs were processed whole due to lack of reporter systems in the primate. Mixed expression of epiblast and PrE markers is therefore expected from late ICM (Figures 5C and 5D). Samples clustered largely by developmental stage (Figure S5H). Substantial differences between species were apparent (Figure 5B), embodied primarily in the first diffusion coefficient. The second coefficient appears to reflect progression of development in both mammals.

Expression of many pluripotency factors, including *Pou5f1*, *Sox2*, *Nanog*, *Esrrb*, *Klf4*, *Tbx3*, and *Tdgf1*, was conserved between species (Figure 5C). However, we noted changes in PluriNet82 connectivity imparted by the absence of *KLF2*, *FBXO15*, *NR0B1*, and *GBX2* in marmoset (Figure 2B). Two of these, *Klf2* and *Gbx2*, are proposed core naive pluripotency regulators in mouse ESCs (Figure 3E). We then assessed localization of naive pluripotency factors by immunofluorescence at the early-mid blastocyst stage. KLF4 and TFCP2L1 were predominantly expressed in the ICM and largely correlated with NANOG, albeit with slightly broader distribution (Figures 5D and 5E). These factors are thus coexpressed in a subset of ICM cells in the marmoset blastocyst, as also observed in human (Takashima et al., 2014). Intriguingly, E-CADHERIN staining was intense in the trophectoderm but diffuse in the ICM (Figure 5E).

Distinctions evident from the wider pluripotency network prompted us to investigate potential differences in epiblast and PrE specification in primate ICM. In mouse, sequential activation of early PrE markers (*Gata6*, *Pdgfra*) is followed by upregulation of late markers (*Gata4*, *Foxa2*) upon lineage segregation (Figure 5F). The majority of key PrE regulators, including *GATA6*, *SOX17*, and *GATA4*, were also present at high abundance in marmoset ICM. We performed further immunostaining for *GATA4* and *GATA6* to assess specificity to PrE. Initially, NANOG and GATA6 were coexpressed in the early ICM (Figure 5G), similar to mouse (Plusa et al., 2008; Schrode et al., 2014) and human (Roode et al., 2012). At the mid-blastocyst stage, we observed mutually exclusive staining of NANOG and SOX17 (Figure 5H). KLF4 was expressed at low levels throughout the

(C) Selected components of WNT and JAK/STAT signaling pathways for the samples indicated.

(D) Ternary plot of the most divergent transcriptional and epigenetic regulators between preimplantation epiblast, ESC, and diapaused epiblast. FPKM values are scaled to the mean across the three cell types and are log transformed.

(E) Differentially expressed genes in FPKM between preimplantation epiblast, diapaused epiblast, and ESC, as indicated.

(F) Diffusion map based on dynamically expressed genes from morula to postimplantation epiblast, ESC, and diapaused epiblast.

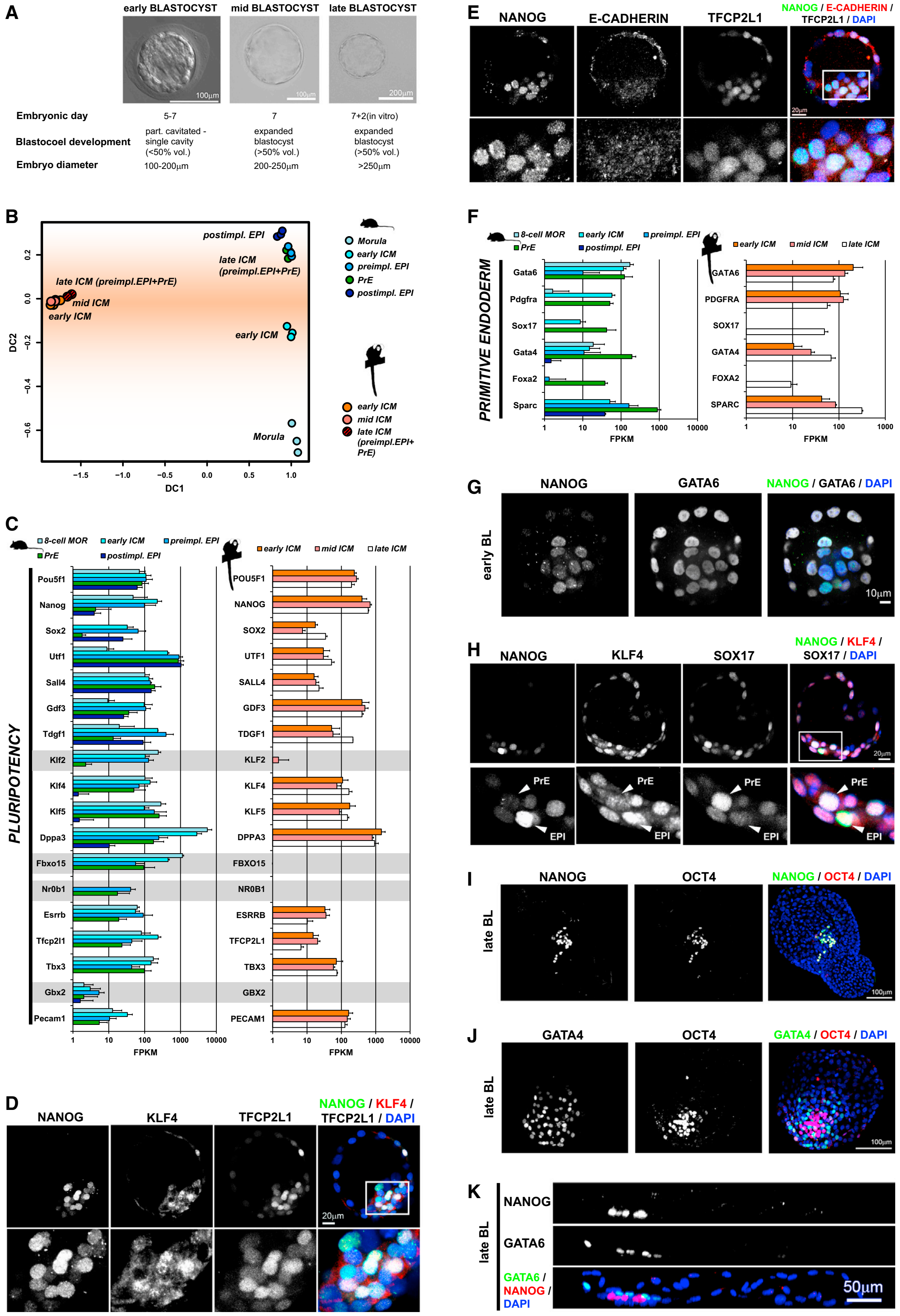

(legend on next page)

embryo, but with stronger signal in NANOG-positive cells of the ICM (Figure 5H). OCT4 tightly colocalized with NANOG in the presumptive epiblast of late blastocysts (Figure 5I). In contrast, GATA4 and OCT4 staining were mutually exclusive in the ICM, with GATA4 specific to PrE and OCT4 confined to the epiblast (Figure 5J). Confocal microscopy of late marmoset blastocysts revealed that GATA6-positive cells formed a distinct layer overlying NANOG-positive cells within the ICM, indicating restriction of GATA6 to PrE (Figure 5K). We conclude that mouse and marmoset share the majority of PrE specifiers.

FGF and WNT Signaling Are Required for Lineage Segregation in the Marmoset Blastocyst

To further relate mouse and marmoset embryos, we combined data from E4.5 epiblast and PrE mouse samples to allow comparison to the late marmoset ICM (Figure S5J; Table S7). Hierarchical clustering of an independent panel of genes selected by the International Stem Cell Initiative (Adewumi et al., 2007) revealed broad conservation in expression patterns of many pluripotency and lineage markers but differences in signaling pathway components, including *Lifr*, *Fgf4*, and *Nodal* (Figure S5J). Knowledge of pathways regulating PrE segregation and epiblast specification in primates is limited. We focused on the regulation of pathway components at the early ICM stage, when *NANOG* and *GATA6* are coexpressed (Figure 5G) and individual cells are indistinguishable at the transcriptome level in mouse (Ohnishi et al., 2014). The most significant differentially expressed pathways in marmoset included ascorbate and aldorate, inositol phosphate and lipoic acid metabolism, lysine biosynthesis, and PPAR signaling (Figure S5I). Pathways upregulated in mouse included arginine and proline metabolism and proteasome, estrogen receptor, and mTOR signaling. These results highlight metabolic differences, in particular with regard to amino acid biosynthesis, between the mouse and marmoset embryo.

We then examined signaling pathways by combining gene expression with KEGG pathway maps. We observed pronounced differences in major cascades such as transforming growth factor β (TGF-β), fibroblast growth factor (FGF), and WNT (Figures 6A–6C; Figure S6). Marmoset early ICM cells expressed high levels of *ACVR1B* (*ALK4*), *TGFBR1* (*ALK5*), and *ACVR2A* (Figure 6A; Figure S6A). Interestingly, *BMP4* is not detected in marmoset (Figure S6D), but *NODAL* is expressed from the early blastocyst stage (Figure 6A; Figure S6A). *FGF4* is absent in the early ICM and is upregulated at later stages. Additionally, we noted altered levels of FGF receptor expression (Figure 6C; Figure S6C). In relation to WNT signaling, the secreted Wnt inhibitor *DKK1* and the negative regulator of β-catenin *GSK3β* were highly upregulated, which together with reduced β-catenin (*CTNNB1*) (Figure 6B; Figure S6B) may indicate suppression of WNT signaling in the early primate ICM. At the late blastocyst stage, however, *DKK1* diminished, concomitant with an increase in *CDX1*. This suggests the possibility of a specific role for the WNT pathway in the marmoset blastocyst. Thus, although transcription factor expression is largely conserved between the rodent and primate embryo stages examined, the repertoire of signaling pathway components differs.

To investigate the functional relevance of these expression patterns, marmoset morulae were cultured to the late blastocyst stage in the presence of selective pathway inhibitors (Figure 6D). Embryos were subsequently immunostained for NANOG, GATA6, and CDX2. Cell number and fluorescence intensities were measured with automated analysis software (Volocity; PerkinElmer) (Figure 6E, left column; Figures S7A–S7F). Mouse embryos were cultured and analyzed under identical conditions for direct comparison. Inhibitor treatment did not affect total cell number (Figure S7G). In marmoset DMSO-treated controls, the epiblast and PrE had segregated in late blastocysts, as indicated by mutually exclusive NANOG (NANOG-only) and GATA6 (GATA6-only) staining in subsets of inner cells (Figure 6D, arrowheads). GATA6 was also expressed in the trophectoderm, but at lower levels and in combination with CDX2 (Figures S7G and S7H). The ratio of epiblast (NANOG-only) to PrE (GATA6-only) cells was higher in marmoset embryos compared to mouse (Figure 6E).

In mouse embryos, inhibition of FGF signaling ablated PrE formation and increased the epiblast compartment (Figure 6E), whereas inhibition of WNT and TGF-β/Nodal signaling did not elicit significant effects, consistent with previous reports (Biechele et al., 2013; Nichols et al., 2009; Yamanaka et al., 2010). In marmoset, blocking the type I TGF-β/activin/Nodal receptor with A8301 did not disrupt lineage segregation (arrowheads for GATA6-only cells in Figure 6D). However, inhibition of ERK or WNT signaling led to coexpression patterns rarely observed in control embryos (Figure 6E; Figure S7H). NANOG-only cells in marmoset embryos increased upon ERK pathway inhibition (Figures 6D–6F). WNT signaling inhibition led to strong upregulation of NANOG, GATA6, and CDX2, and in particular the number of NANOG-high cells was substantially greater (Figures 6D, 6E, and 6G). Strikingly, emergence of GATA6-only PrE cells was suppressed by both ERK and WNT signaling inhibition (Figure 6H; Figure S7I).

We assessed whether the observed effects of TGF-β, WNT, and ERK inhibition might be combined by carrying out dual-inhibition experiments (Figure 7A). Conditions including WNT inhibition led to greater proportions of NANOG-only cells (Figure 7B), with a substantial increase in NANOG-high cells (Figure 7C). PrE formation was impaired in all dual-inhibition experiments (Figures 7A and 7D). The most profound reduction occurred when both WNT and ERK signaling were inhibited (Figure 7D),

Figure 5. Pluripotency Factors Are Conserved, whereas Signaling Receptors Diverge, in Mouse and Marmoset ICM

(A) Staging criteria for isolation of early, mid, and late marmoset ICM and images of the blastocysts analyzed.

(B) Diffusion map of mouse and marmoset embryonic samples.

(C) Pluripotency gene expression for mouse and marmoset embryonic samples. Error bars represent SD.

(D and E) Confocal z projections of whole-mount marmoset early-mid blastocyst immunofluorescence stainings for the markers indicated.

(F) PrE-associated gene expression for mouse and marmoset embryonic samples. Error bars represent SD.

(G–J) Immunofluorescence stainings of early, mid (H), and late marmoset blastocyst.

(K) z-x cross-section of marmoset blastocyst stained for the markers indicated.

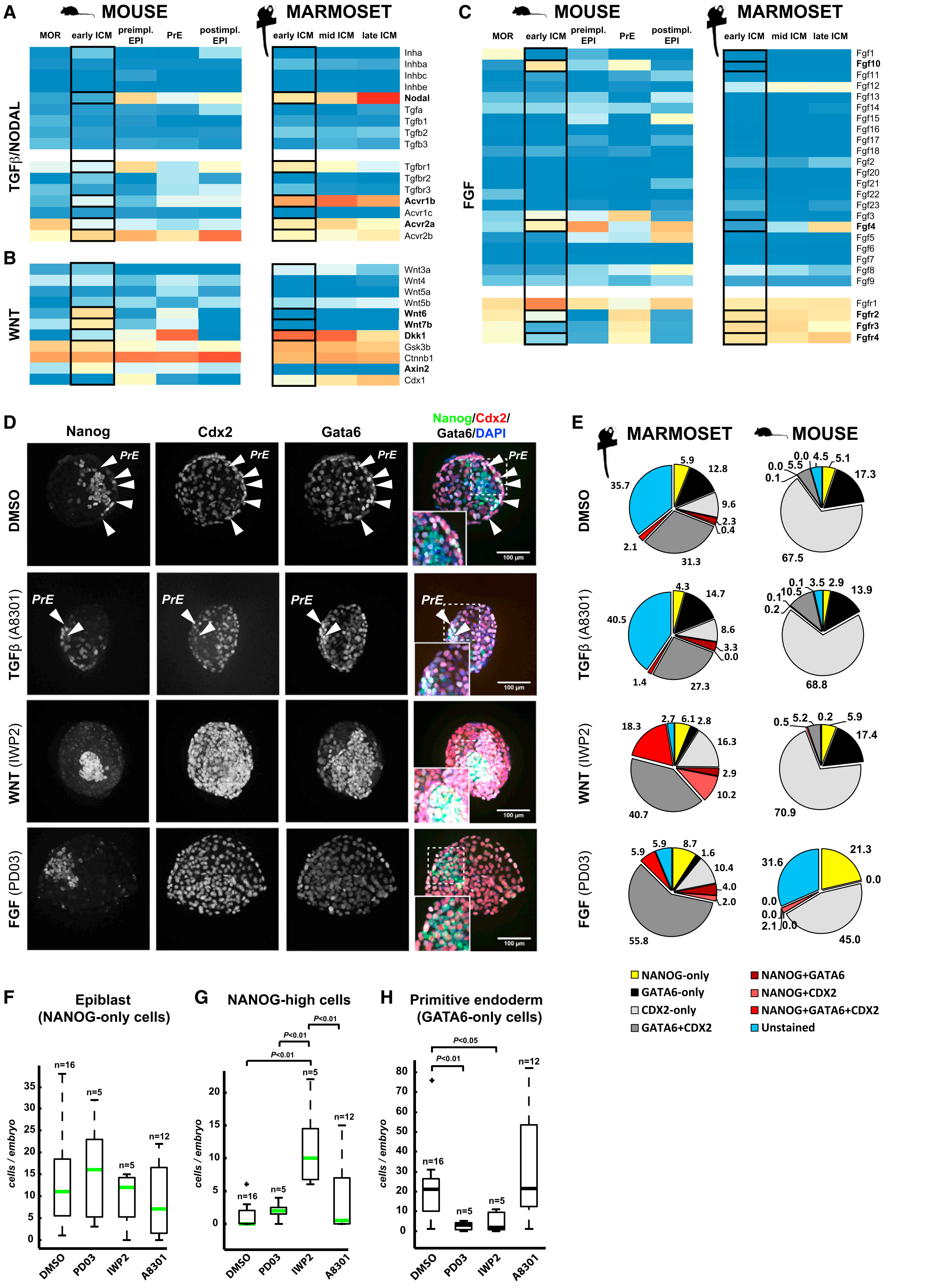

(legend on next page)

indicating an additive effect. These results support a conserved role for FGF/ERK signaling and a marmoset-specific function for WNT signaling in ICM lineage segregation (Figure 7E).

DISCUSSION

In this study, we applied single-cell RNA-seq protocols to clusters of cells to determine genome-wide transcriptional activity in developing embryonic lineages. This analysis defined stage-specific gene expression modules of lineage identity and fate specification. Furthermore, our data establish that mouse ESCs and diapaused epiblasts sustain preimplantation epiblast identity despite major environmental or metabolic changes. We found the regulatory network governing naive pluripotency in ESCs to be progressively activated in the embryo and maximally around E4.5. Heterogeneous ICM cells may individually progress to this stage over the preceding 12–18 hr. Indeed, from E3.75 onward, ESCs can be derived from single ICM cells in stringent 2i/LIF culture (Boroviak et al., 2014).

The gene expression module of the pre- and postimplantation epiblast contains drivers that act to dismantle the naive pluripotency circuitry. Prominent examples include *Fgf4*, an activator of Erk signaling and subsequent differentiation (Kunath et al., 2007), and *Tcf7l1* (*Tcf3*), a repressor of naive pluripotency (Martello et al., 2012; Wray et al., 2011; Yi et al., 2008). Consequently, naive pluripotency factors are abruptly eliminated upon implantation. These data are inconsistent with the proposition that pluripotency is an inherently precarious balance, wherein pluripotency factors act continuously as competing lineage specifiers (Loh and Lim, 2011). Restriction to preimplantation development precludes these factors from playing a role in lineage specification. We propose that pluripotency is not intrinsically poised for differentiation but progresses through at least three phases: naive, formative, and primed (Kalkan and Smith, 2014). Lineage-specific profiling of the E5.5 epiblast is expected to capture the fundamental attributes of the formative phase.

We uncovered parallels in signaling activity between ESC and diapause. Stabilization of β-catenin via GSK3 inhibition is important for maintenance of naive pluripotency in mouse ESCs (Wray et al., 2011; Ying et al., 2008). Abrogation of *Tcf7l1* function by β-catenin results in stable expression of the key pluripotency factors *Esrrb*, *Klf2*, and *Nanog* (Martello et al., 2012; Wray et al., 2011). This effect of GSK3 inhibition can be partially reproduced by WNT, and together with either LIF stimulation or MAPK/ERK kinase (MEK) inhibition allows continuous propagation of mouse ESCs (Dunn et al., 2014; ten Berge et al., 2011; Wray et al., 2011; Ying et al., 2008). In diapaused epiblasts, we identified high levels of the Jak/Stat downstream targets *Klf4* and *Tfcp2l1* (Bourillot et al., 2009; Martello et al., 2013) and expression of *Lifr* and *Il6st* (*gp130*), reflecting the essential role of LIF signaling in diapause (Nichols et al., 2001). We also observed strong *Wnt4* and *Axin2* expression in diapause, potentially indicative of WNT signaling activity. Because *Tcf7l1* is expressed during diapause, WNT signaling may be important for sustaining naive pluripotency in the dormant epiblast. We speculate that pathways evolved to mediate developmental arrest in vivo may facilitate in vitro capture of the naive state (Nichols et al., 2001; Nichols and Smith, 2012).

In primate embryos, we found the majority of pluripotency-associated genes to be expressed in the ICM. However, absence of KLF2, NR0B1, FBXO15, and BMP4 suggests adaptations in the wider pluripotency network. Similar patterns are observed in human embryos (Blakeley et al., 2015; Yan et al., 2013), lending support for a high degree of conservation of core regulatory interactions in primates. Indeed, the majority of human epiblast-specific factors including KLF17, LEFTY1, and NODAL (Blakeley et al., 2015) are also expressed in the late marmoset ICM. These data confirm similarities between human and marmoset, and highlight the relevance of tractable nonhuman primate species as a model for early development.

Transcriptional data revealed dissimilar expression of FGF, WNT, and TGF-β/Nodal pathway genes. In human, there are conflicting reports regarding the role of TGF-β/Nodal signaling in the embryo (Blakeley et al., 2015; Van der Jeught et al., 2014). We show that NANOG expression in the marmoset ICM does not require FGF, WNT, and TGF-β/Nodal signaling. In particular, we noted an increase in NANOG-positive cells when ERK activation is inhibited by PD03. Robust expression of *Nanog* in the absence of FGF/ERK signaling is reported in a variety of species, including mouse, rat, bovine, and human blastocysts (Kuijk et al., 2012; Nichols et al., 2009; Roode et al., 2012), and may present a general feature of naive pluripotency in mammals. Recent advances in the generation of naive pluripotent human ESCs (Takashima et al., 2014; Theunissen et al., 2014) provide support for this hypothesis.

Suppression of MEK via PD03 blocks PrE formation in rodent embryos (Arman et al., 1998; Kuijk et al., 2012; Nichols et al., 2009; Ralston and Rossant, 2005; Roode et al., 2012; Yamanaka et al., 2010). This effect seems to be attenuated in human (Kuijk et al., 2012; Roode et al., 2012), suggesting involvement of additional mechanisms. We found that WNT inhibition increased NANOG, GATA6, and CDX2 in marmoset embryos, leading to a blurring of lineage boundaries. Cells failing to undergo lineage specification may remain trapped at an earlier developmental stage when NANOG and GATA6 are coexpressed. Interference with ERK signaling reduced the number of PrE cells and broadened expression of CDX2 and NANOG. We propose that lineage specification in primate preimplantation development is regulated by both WNT and FGF/ERK pathways,

Figure 6. FGF and WNT Inhibition Disrupt Lineage Segregation in the Marmoset Blastocyst

(A–C) Expression of selected components of the TGF-β/NODAL, FGF, and WNT signaling pathways.

(D) Confocal z projections of inhibitor-treated marmoset late blastocysts stained for NANOG, CDX2, GATA6, and DAPI.

(E) Fluorescence signal from cells labeled for lineage markers in mouse and marmoset embryos. Morulae were cultured under identical conditions to the late blastocyst stage in the presence of A8301 (3 μM), PD0325901 (3 μM), IWP2 (3 μM), or DMSO (control) for 3 and 4 days in mouse and marmoset, respectively.

(F–H) Quantification of (F) NANOG-only, (G) NANOG-high, and (H) GATA6-only cells. Plotted are the first and second quartiles of data points (boxed) with error bars at minimum and maximum values. Outliers are indicated with a cross. NANOG-high cells displayed at least 1.5× average NANOG fluorescence intensity. p values were computed by one-way ANOVA with Tukey HSD (honest significant difference) testing.

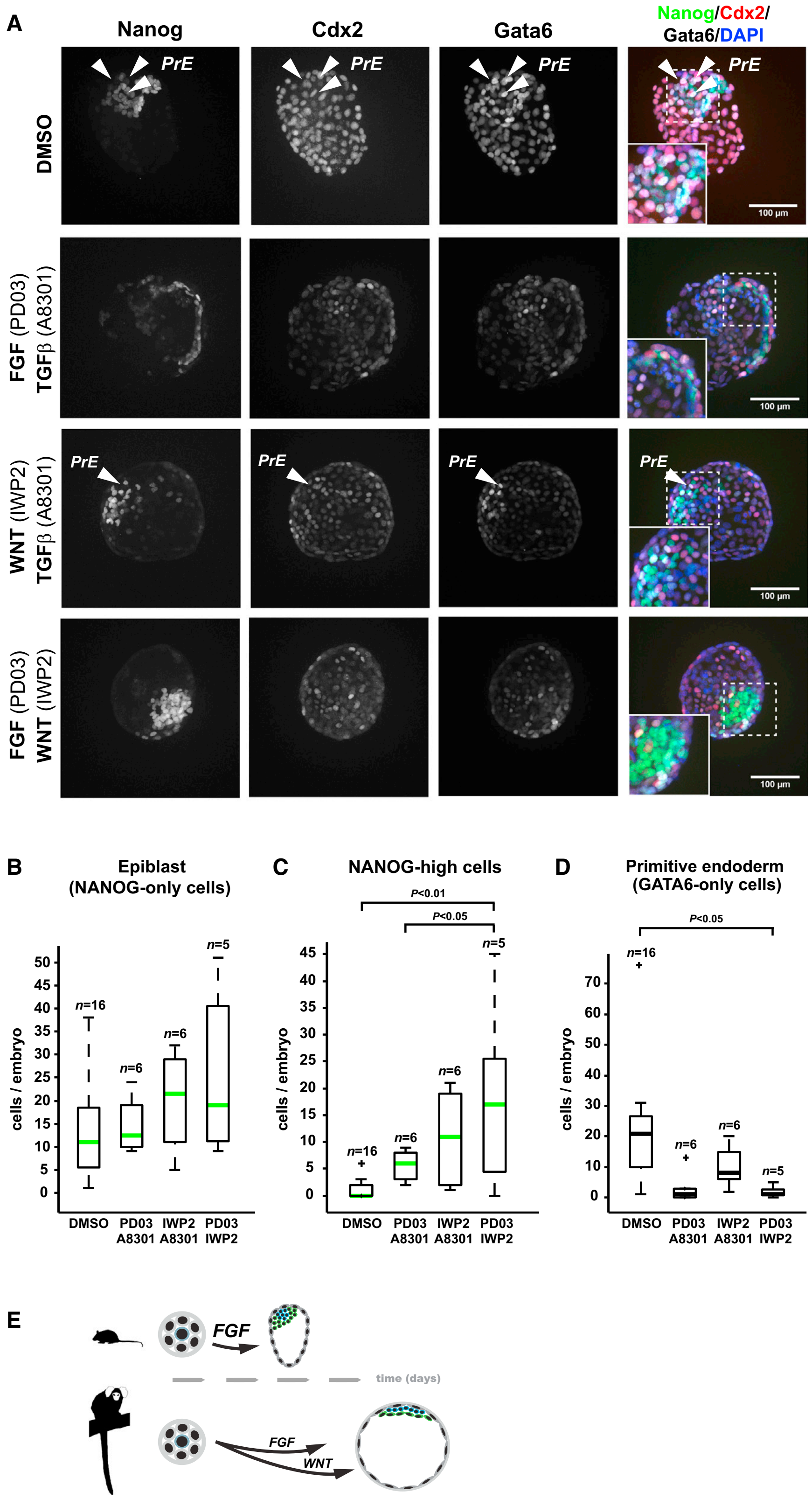

Figure 7. Dual FGF and WNT Inhibition Increases NANOG Levels and Blocks PrE Formation

(A) Confocal z projections of inhibitor-treated marmoset late blastocysts stained for NANOG, CDX2, GATA6, and DAPI.

(B–D) Fluorescence quantification of (B) NANOG-only, (C) NANOG-high, and (D) GATA6-only cells. Outliers are indicated with a cross. NANOG-high cells displayed at least 1.5× average NANOG fluorescence intensity. p values were computed by one-way ANOVA with Tukey HSD testing.

(E) Model of pathways driving lineage specification in mouse and marmoset ICM.

in contrast to mouse, where FGF/ERK is the primary and sufficient driver (Figure 7E).

This study provides a comprehensive resource for identifying the factors and pathways that ignite and extinguish naive pluripotency in vivo. Knowledge gained should be valuable for evaluating and optimizing ESC differentiation and reprogramming to pluripotency in vitro. A further application may be to influence lineage decisions in embryo culture in order to facilitate stem cell derivation or even to improve human blastocyst development for assisted conception.

EXPERIMENTAL PROCEDURES

Mouse Strains and Embryo Collection

Mice used were intercrosses of *Pdgfra::GFP* (Hamilton et al., 2003) and strain 129 estrus-checked females. Embryos were collected at the relevant stages from the oviduct or uterus in M2 medium (Sigma). Embryonic diapause was induced by surgical removal of both ovaries on the morning of the third day of pregnancy (E2.5). Diapaused embryos were flushed on the seventh day of pregnancy. Experiments were performed in accordance with EU guidelines for the care and use of laboratory animals, and under authority of UK governmental legislation. Use of animals in this project was approved by the ethical review committee for the University of Cambridge, and relevant Home Office licenses are in place.

Marmoset Colony Maintenance and Embryo Collection

Primate embryos were obtained from the German Primate Center, Göttingen (Deutsches Primatenzentrum; DPZ) and the Central Institute for Experimental Animals, Kanagawa, Japan (CIEA). Marmoset blastocysts were retrieved by nonsurgical uterine flush according to established methods using recently developed devices (Takahashi et al., 2014; Thomson et al., 1994). Because the time of conception must be determined retrospectively and with an accuracy of ±24 hr, we applied additional criteria to embryo staging such as blastocoel formation and diameter (Figure 5A). Staging of female reproductive cycles and protocols for embryo retrieval have been described (Hanazawa et al., 2012). Animals were obtained from self-sustaining colonies and housed according to standard husbandry guidelines. Protocols for the use of animals and institutional regulations for the care and experimental use of marmosets were strictly followed. Experiments at the DPZ were conducted under license number AZ 33.42502–066/06. Experiments using marmosets at the CIEA were approved by the animal research committee (CIEA 11028) and performed in compliance with guidelines set forth by the Science Council of Japan.

Isolation of Single-Cell Suspensions

Mouse (E3.25–E4.5) and marmoset blastocysts were subjected to immunosurgery as previously described (Nichols et al., 1998; Solter and Knowles, 1975). ICMs were subsequently dissociated from residual trophectoderm with a narrowly fitting Pasteur pipette. For postimplantation mouse embryos, the epiblast was isolated by manual dissection. Dissociation of morulae, ICM, and postimplantation epiblast was carried out in a mixture of trypsin and chick serum (see Supplemental Experimental Procedures). Cells were dissociated by repetitive pipetting using blunted microcapillaries.

Embryonic Stem Cell Culture

E14TG2a cells derived from mouse strain 129/Ola (Hooper et al., 1987) were used as a reference ESC line and cultured in 2i/LIF conditions (Ying et al., 2008). N2B27 (1:1 DMEM/F-12 and Neurobasal media, N2 [in-house] and B27 [GIBCO] additives, 2 mM L-glutamine, and 100 μM β-mercaptoethanol) was supplemented with 1 μM PD0325901, 3 μM CHIR99021, and 10 ng/ml LIF (in-house), and cells were maintained in gelatin-coated (0.1%) culture vessels. Cells were dissociated by conventional methods, and 20 cells per sample were manually selected with a blunt microcapillary.

Transcriptome Analysis

Library construction was carried out using whole-transcriptome preamplification (Tang et al., 2009, 2010b) followed by sonication of cDNA and preparation of Illumina-compatible sequencing constructs (see Supplemental Experimental Procedures). Sequencing reads were processed to remove preamplification adapters and were aligned with GSNAP (Wu and Nacu, 2010). Global analyses were based on variance-stabilized counts computed with the Bioconductor package DESeq (Anders and Huber, 2010). Differential expression analysis was performed in DESeq. Gene clusters were identified by hierarchical clustering on scaled FPKMs. The GOstats R package (Falcon and Gentleman, 2007) was used for GO category and KEGG pathway enrichment analysis. High-level comparative analysis of KEGG pathways was based on the mean expression of constituent genes. For analysis of individual pathways, expression values were mapped onto pathway nodes using PathVisio (Kutmon et al., 2015). PluriNet82 was created by intersecting PluriNetWork (Som et al., 2010) with dynamically expressed genes. Preferential activity at a given embryonic stage was defined as positive-scaled log-normalized expression for a network node; a network edge was required to have active source and target nodes. Cytoscape (Smoot et al., 2011) was used for network visualization.

ACCESSION NUMBERS

The accession numbers for the mouse and marmoset sequencing data reported in this paper are ArrayExpress: E-MTAB-2958, E-MTAB-2959.

SUPPLEMENTAL INFORMATION

Supplemental Information includes Supplemental Experimental Procedures, seven figures, and seven tables and can be found with this article online at http://dx.doi.org/10.1016/j.devcel.2015.10.011.

AUTHOR CONTRIBUTIONS

P.B., A.S., and J.N. conceived the study; T.B., P.B., and J.N. developed the experimental approach and embryo sequencing workflow; T.B. and P.B. processed samples and prepared sequencing libraries; R.L., P.L., and P.B. performed computational analyses; T.B. and J.O. carried out marmoset embryo experiments under the guidance of E.S. and R.B.; T.B., R.L., A.S., and P.B. wrote the paper.

ACKNOWLEDGMENTS

We thank Peter Humphreys for assistance with imaging, and Samuel Jameson and staff for mouse husbandry. We are grateful to Charis Drummer, Ayako Sedohara, Akiko Shimada, Yuko Yamada, Ryo Oiwa, and Takeshi Kuge for technical support with marmoset embryo recovery. Illumina sequencing was provided by Bettina Haase and Dinko Pavlinic at the EMBL Genomics Core Facility. This work was supported by funding from the Wellcome Trust, the Genome Biology Unit of the European Molecular Biology Laboratory, BBSRC grants BB/G015678/1 and BB/M004023/1, an MRC Centenary Award, and the Louis Jeantet Foundation. A.S. is a Medical Research Council Professor.

Received: October 3, 2014
Revised: September 1, 2015
Accepted: October 14, 2015
Published: November 9, 2015

REFERENCES

Adewumi, O., Aflatoonian, B., Ahrlund-Richter, L., Amit, M., Andrews, P.W., Beighton, G., Bello, P.A., Benvenisty, N., Berry, L.S., Bevan, S., et al.; International Stem Cell Initiative (2007). Characterization of human embryonic stem cell lines by the International Stem Cell Initiative. Nat. Biotechnol. *25*, 803–816.

Anders, S., and Huber, W. (2010). Differential expression analysis for sequence count data. Genome Biol. *11*, R106.

Arman, E., Haffner-Krausz, R., Chen, Y., Heath, J.K., and Lonai, P. (1998). Targeted disruption of fibroblast growth factor (FGF) receptor 2 suggests a role for FGF signaling in pregastrulation mammalian development. Proc. Natl. Acad. Sci. USA *95*, 5082–5087.

Artus, J., Piliszek, A., and Hadjantonakis, A.K. (2011). The primitive endoderm lineage of the mouse blastocyst: sequential transcription factor activation and regulation of differentiation by Sox17. Dev. Biol. *350*, 393–404.

Bedzhov, I., and Zernicka-Goetz, M. (2014). Self-organizing properties of mouse pluripotent cells initiate morphogenesis upon implantation. Cell *156*, 1032–1044.

Biechele, S., Cockburn, K., Lanner, F., Cox, B.J., and Rossant, J. (2013). Porcn-dependent Wnt signaling is not required prior to mouse gastrulation. Development *140*, 2961–2971.

Blakeley, P., Fogarty, N.M., Del Valle, I., Wamaitha, S.E., Hu, T.X., Elder, K., Snell, P., Christie, L., Robson, P., and Niakan, K.K. (2015). Defining the three cell lineages of the human blastocyst by single-cell RNA-seq. Development *142*, 3151–3165.

Boroviak, T., Loos, R., Bertone, P., Smith, A., and Nichols, J. (2014). The ability of inner-cell-mass cells to self-renew as embryonic stem cells is acquired following epiblast specification. Nat. Cell Biol. *16*, 516–528.

Bourillot, P.Y., Aksoy, I., Schreiber, V., Wianny, F., Schulz, H., Hummel, O., Hubner, N., and Savatier, P. (2009). Novel STAT3 target genes exert distinct roles in the inhibition of mesoderm and endoderm differentiation in cooperation with Nanog. Stem Cells *27*, 1760–1771.

Brennecke, P., Anders, S., Kim, J.K., Kołodziejczyk, A.A., Zhang, X., Proserpio, V., Baying, B., Benes, V., Teichmann, S.A., Marioni, J.C., and Heisler, M.G. (2013). Accounting for technical noise in single-cell RNA-seq experiments. Nat. Methods *10*, 1093–1095.

Brons, I.G., Smithers, L.E., Trotter, M.W., Rugg-Gunn, P., Sun, B., Chuva de Sousa Lopes, S.M., Howlett, S.K., Clarkson, A., Ahrlund-Richter, L., Pedersen, R.A., and Vallier, L. (2007). Derivation of pluripotent epiblast stem cells from mammalian embryos. Nature *448*, 191–195.

Brook, F.A., and Gardner, R.L. (1997). The origin and efficient derivation of embryonic stem cells in the mouse. Proc. Natl. Acad. Sci. USA *94*, 5709–5712.

Buehr, M., Meek, S., Blair, K., Yang, J., Ure, J., Silva, J., McLay, R., Hall, J., Ying, Q.L., and Smith, A. (2008). Capture of authentic embryonic stem cells from rat blastocysts. Cell *135*, 1287–1298.

Chan, Y.S., Göke, J., Ng, J.H., Lu, X., Gonzales, K.A., Tan, C.P., Tng, W.Q., Hong, Z.Z., Lim, Y.S., and Ng, H.H. (2013). Induction of a human pluripotent state with distinct regulatory circuitry that resembles preimplantation epiblast. Cell Stem Cell *13*, 663–675.

Chazaud, C., Yamanaka, Y., Pawson, T., and Rossant, J. (2006). Early lineage segregation between epiblast and primitive endoderm in mouse blastocysts through the Grb2-MAPK pathway. Dev. Cell *10*, 615–624.

Dunn, S.J., Martello, G., Yordanov, B., Emmott, S., and Smith, A.G. (2014). Defining an essential transcription factor program for naïve pluripotency. Science *344*, 1156–1160.

Evans, M.J., and Kaufman, M.H. (1981). Establishment in culture of pluripotential cells from mouse embryos. Nature *292*, 154–156.

Falcon, S., and Gentleman, R. (2007). Using GOstats to test gene lists for GO term association. Bioinformatics *23*, 257–258.

Fang, R., Liu, K., Zhao, Y., Li, H., Zhu, D., Du, Y., Xiang, C., Li, X., Liu, H., Miao, Z., et al. (2014). Generation of naive induced pluripotent stem cells from rhesus monkey fibroblasts. Cell Stem Cell *15*, 488–496.

Frum, T., Halbisen, M.A., Wang, C., Amiri, H., Robson, P., and Ralston, A. (2013). Oct4 cell-autonomously promotes primitive endoderm development in the mouse blastocyst. Dev. Cell *25*, 610–622.

Gerbe, F., Cox, B., Rossant, J., and Chazaud, C. (2008). Dynamic expression of Lrp2 pathway members reveals progressive epithelial differentiation of primitive endoderm in mouse blastocyst. Dev. Biol. *313*, 594–602.

Grün, D., Kester, L., and van Oudenaarden, A. (2014). Validation of noise models for single-cell transcriptomics. Nat. Methods *11*, 637–640.

Guo, G., Huss, M., Tong, G.Q., Wang, C., Li Sun, L., Clarke, N.D., and Robson, P. (2010). Resolution of cell fate decisions revealed by single-cell gene expression analysis from zygote to blastocyst. Dev. Cell *18*, 675–685.

Hall, J., Guo, G., Wray, J., Eyres, I., Nichols, J., Grotewold, L., Morfopoulou, S., Humphreys, P., Mansfield, W., Walker, R., et al. (2009). Oct4 and LIF/Stat3 additively induce Krüppel factors to sustain embryonic stem cell self-renewal. Cell Stem Cell *5*, 597–609.

Hamilton, T.G., Klinghoffer, R.A., Corrin, P.D., and Soriano, P. (2003). Evolutionary divergence of platelet-derived growth factor alpha receptor signaling mechanisms. Mol. Cell. Biol. *23*, 4013–4025.

Hanazawa, K., Mueller, T., Becker, T., Heistermann, M., Behr, R., and Sasaki, E. (2012). Minimally invasive transabdominal collection of preimplantation embryos from the common marmoset monkey (*Callithrix jacchus*). Theriogenology *78*, 811–816.

Handyside, A.H. (1978). Time of commitment of inside cells isolated from preimplantation mouse embryos. J. Embryol. Exp. Morphol. *45*, 37–53.

Hooper, M., Hardy, K., Handyside, A., Hunter, S., and Monk, M. (1987). HPRT-deficient (Lesch-Nyhan) mouse embryos derived from germline colonization by cultured cells. Nature *326*, 292–295.

Kalkan, T., and Smith, A. (2014). Mapping the route from naive pluripotency to lineage specification. Philos. Trans. R. Soc. Lond. B Biol. Sci. *369*, 20130540.

Ketchel, M.M., Banik, U.K., and Mantalenakis, S.J. (1966). A study of delayed implantation caused by parabiosis in pregnant rats. J. Reprod. Fertil. *11*, 213–219.

Kharchenko, P.V., Silberstein, L., and Scadden, D.T. (2014). Bayesian approach to single-cell differential expression analysis. Nat. Methods *11*, 740–742.

Kishi, N., Sato, K., Sasaki, E., and Okano, H. (2014). Common marmoset as a new model animal for neuroscience research and genome editing technology. Dev. Growth Differ. *56*, 53–62.

Kuijk, E.W., van Tol, L.T., Van de Velde, H., Wubbolts, R., Welling, M., Geijsen, N., and Roelen, B.A. (2012). The roles of FGF and MAP kinase signaling in the segregation of the epiblast and hypoblast cell lineages in bovine and human embryos. Development *139*, 871–882.

Kunath, T., Saba-El-Leil, M.K., Almousailleakh, M., Wray, J., Meloche, S., and Smith, A. (2007). FGF stimulation of the Erk1/2 signalling cascade triggers transition of pluripotent embryonic stem cells from self-renewal to lineage commitment. Development *134*, 2895–2902.

Kurimoto, K., Yabuta, Y., Ohinata, Y., Ono, Y., Uno, K.D., Yamada, R.G., Ueda, H.R., and Saitou, M. (2006). An improved single-cell cDNA amplification method for efficient high-density oligonucleotide microarray analysis. Nucleic Acids Res. *34*, e42.

Kutmon, M., van Iersel, M.P., Bohler, A., Kelder, T., Nunes, N., Pico, A.R., and Evelo, C.T. (2015). PathVisio 3: an extendable pathway analysis toolbox. PLoS Comput. Biol. *11*, e1004085.

Lafon, S., Keller, Y., and Coifman, R.R. (2006). Data fusion and multicue data matching by diffusion maps. IEEE Trans. Pattern Anal. Mach. Intell. *28*, 1784–1797.

Le Bin, G.C., Muñoz-Descalzo, S., Kurowski, A., Leitch, H., Lou, X., Mansfield, W., Etienne-Dumeau, C., Grabole, N., Mulas, C., Niwa, H., et al. (2014). Oct4 is required for lineage priming in the developing inner cell mass of the mouse blastocyst. Development *141*, 1001–1010.

Lee, H.J., Hore, T.A., and Reik, W. (2014). Reprogramming the methylome: erasing memory and creating diversity. Cell Stem Cell *14*, 710–719.

Leitch, H.G., McEwen, K.R., Turp, A., Encheva, V., Carroll, T., Grabole, N., Mansfield, W., Nashun, B., Knezovich, J.G., Smith, A., et al. (2013). Naive pluripotency is associated with global DNA hypomethylation. Nat. Struct. Mol. Biol. *20*, 311–316.

Li, P., Tong, C., Mehrian-Shai, R., Jia, L., Wu, N., Yan, Y., Maxson, R.E., Schulze, E.N., Song, H., Hsieh, C.L., et al. (2008). Germline competent embryonic stem cells derived from rat blastocysts. Cell *135*, 1299–1310.

Loh, K.M., and Lim, B. (2011). A precarious balance: pluripotency factors as lineage specifiers. Cell Stem Cell *8*, 363–369.

Lustig, B., Jerchow, B., Sachs, M., Weiler, S., Pietsch, T., Karsten, U., van de Wetering, M., Clevers, H., Schlag, P.M., Birchmeier, W., and Behrens, J. (2002). Negative feedback loop of Wnt signaling through upregulation of conductin/axin2 in colorectal and liver tumors. Mol. Cell. Biol. *22*, 1184–1193.

Mantalenakis, S.J., and Ketchel, M.M. (1966). Frequency and extent of delayed implantation in lactating rats and mice. J. Reprod. Fertil. *12*, 391–394.

Marks, H., Kalkan, T., Menafra, R., Denissov, S., Jones, K., Hofemeister, H., Nichols, J., Kranz, A., Stewart, A.F., Smith, A., and Stunnenberg, H.G. (2012). The transcriptional and epigenomic foundations of ground state pluripotency. Cell *149*, 590–604.

Martello, G., Sugimoto, T., Diamanti, E., Joshi, A., Hannah, R., Ohtsuka, S., Göttgens, B., Niwa, H., and Smith, A. (2012). Esrrb is a pivotal target of the Gsk3/Tcf3 axis regulating embryonic stem cell self-renewal. Cell Stem Cell *11*, 491–504.

Martello, G., Bertone, P., and Smith, A. (2013). Identification of the missing pluripotency mediator downstream of leukaemia inhibitory factor. EMBO J. *32*, 2561–2574.

Martin, G.R. (1981). Isolation of a pluripotent cell line from early mouse embryos cultured in medium conditioned by teratocarcinoma stem cells. Proc. Natl. Acad. Sci. USA *78*, 7634–7638.

Matter, K., and Balda, M.S. (1999). Occludin and the functions of tight junctions. Int. Rev. Cytol. *186*, 117–146.

Mead, R.A. (1993). Embryonic diapause in vertebrates. J. Exp. Zool. *266*, 629–641.

Nakatsuji, N., and Suemori, H. (2002). Embryonic stem cell lines of nonhuman primates. ScientificWorldJournal *2*, 1762–1773.

Nichols, J., and Gardner, R.L. (1984). Heterogeneous differentiation of external cells in individual isolated early mouse inner cell masses in culture. J. Embryol. Exp. Morphol. *80*, 225–240.

Nichols, J., and Smith, A. (2009). Naive and primed pluripotent states. Cell Stem Cell *4*, 487–492.

Nichols, J., and Smith, A. (2012). Pluripotency in the embryo and in culture. Cold Spring Harb. Perspect. Biol. *4*, a008128.

Nichols, J., Zevnik, B., Anastassiadis, K., Niwa, H., Klewe-Nebenius, D., Chambers, I., Schöler, H., and Smith, A. (1998). Formation of pluripotent stem cells in the mammalian embryo depends on the POU transcription factor Oct4. Cell *95*, 379–391.

Nichols, J., Chambers, I., Taga, T., and Smith, A. (2001). Physiological rationale for responsiveness of mouse embryonic stem cells to gp130 cytokines. Development *128*, 2333–2339.

Nichols, J., Silva, J., Roode, M., and Smith, A. (2009). Suppression of Erk signalling promotes ground state pluripotency in the mouse embryo. Development *136*, 3215–3222.

Niwa, H., Ogawa, K., Shimosato, D., and Adachi, K. (2009). A parallel circuit of LIF signalling pathways maintains pluripotency of mouse ES cells. Nature *460*, 118–122.

Ohnishi, Y., Huber, W., Tsumura, A., Kang, M., Xenopoulos, P., Kurimoto, K., Oleś, A.K., Araúzo-Bravo, M.J., Saitou, M., Hadjantonakis, A.K., and Hiiragi, T. (2014). Cell-to-cell expression variability followed by signal reinforcement progressively segregates early mouse lineages. Nat. Cell Biol. *16*, 27–37.

Palmieri, S.L., Peter, W., Hess, H., and Schöler, H.R. (1994). Oct-4 transcription factor is differentially expressed in the mouse embryo during establishment of the first two extraembryonic cell lineages involved in implantation. Dev. Biol. *166*, 259–267.

Pilon, N., Oh, K., Sylvestre, J.R., Savory, J.G., and Lohnes, D. (2007). Wnt signaling is a key mediator of Cdx1 expression in vivo. Development *134*, 2315–2323.

Plusa, B., Piliszek, A., Frankenberg, S., Artus, J., and Hadjantonakis, A.K. (2008). Distinct sequential cell behaviours direct primitive endoderm formation in the mouse blastocyst. Development *135*, 3081–3091.

Ralston, A., and Rossant, J. (2005). Genetic regulation of stem cell origins in the mouse embryo. Clin. Genet. *68*, 106–112.

Renfree, M.B., and Shaw, G. (2000). Diapause. Annu. Rev. Physiol. *62*, 353–375.

Roode, M., Blair, K., Snell, P., Elder, K., Marchant, S., Smith, A., and Nichols, J. (2012). Human hypoblast formation is not dependent on FGF signalling. Dev. Biol. *361*, 358–363.

Rossant, J., and Lis, W.T. (1979). Potential of isolated mouse inner cell masses to form trophectoderm derivatives in vivo. Dev. Biol. *70*, 255–261.

Schrode, N., Saiz, N., Di Talia, S., and Hadjantonakis, A.K. (2014). GATA6 levels modulate primitive endoderm cell fate choice and timing in the mouse blastocyst. Dev. Cell *29*, 454–467.

Shin, K., Fogg, V.C., and Margolis, B. (2006). Tight junctions and cell polarity. Annu. Rev. Cell Dev. Biol. *22*, 207–235.

Sleeman, J.P., and Thiery, J.P. (2011). SnapShot: the epithelial-mesenchymal transition. Cell *145*, 162–162.e1.

Smith, Z.D., Chan, M.M., Mikkelsen, T.S., Gu, H., Gnirke, A., Regev, A., and Meissner, A. (2012). A unique regulatory phase of DNA methylation in the early mammalian embryo. Nature *484*, 339–344.

Smoot, M.E., Ono, K., Ruscheinski, J., Wang, P.L., and Ideker, T. (2011). Cytoscape 2.8: new features for data integration and network visualization. Bioinformatics *27*, 431–432.

Solter, D., and Knowles, B.B. (1975). Immunosurgery of mouse blastocyst. Proc. Natl. Acad. Sci. USA *72*, 5099–5102.

Som, A., Harder, C., Greber, B., Siatkowski, M., Paudel, Y., Warsow, G., Cap, C., Schöler, H., and Fuellen, G. (2010). The PluriNetWork: an electronic representation of the network underlying pluripotency in mouse, and its applications. PLoS ONE *5*, e15165.

Tachibana, M., Sparman, M., Ramsey, C., Ma, H., Lee, H.S., Penedo, M.C., and Mitalipov, S. (2012). Generation of chimeric rhesus monkeys. Cell *148*, 285–295.

Takahashi, T., Hanazawa, K., Inoue, T., Sato, K., Sedohara, A., Okahara, J., Suemizu, H., Yagihashi, C., Yamamoto, M., Eto, T., et al. (2014). Birth of healthy offspring following ICSI in in vitro-matured common marmoset (*Callithrix jacchus*) oocytes. PLoS ONE *9*, e95560.

Takashima, Y., Guo, G., Loos, R., Nichols, J., Ficz, G., Krueger, F., Oxley, D., Santos, F., Clarke, J., Mansfield, W., et al. (2014). Resetting transcription factor control circuitry toward ground-state pluripotency in human. Cell *158*, 1254–1269.

Tang, F., Barbacioru, C., Wang, Y., Nordman, E., Lee, C., Xu, N., Wang, X., Bodeau, J., Tuch, B.B., Siddiqui, A., et al. (2009). mRNA-seq whole-transcriptome analysis of a single cell. Nat. Methods *6*, 377–382.

Tang, F., Barbacioru, C., Bao, S., Lee, C., Nordman, E., Wang, X., Lao, K., and Surani, M.A. (2010a). Tracing the derivation of embryonic stem cells from the inner cell mass by single-cell RNA-seq analysis. Cell Stem Cell *6*, 468–478.

Tang, F., Barbacioru, C., Nordman, E., Li, B., Xu, N., Bashkirov, V.I., Lao, K., and Surani, M.A. (2010b). RNA-seq analysis to capture the transcriptome landscape of a single cell. Nat. Protoc. *5*, 516–535.

ten Berge, D., Kurek, D., Blauwkamp, T., Koole, W., Maas, A., Eroglu, E., Siu, R.K., and Nusse, R. (2011). Embryonic stem cells require Wnt proteins to prevent differentiation to epiblast stem cells. Nat. Cell Biol. *13*, 1070–1075.

Tesar, P.J., Chenoweth, J.G., Brook, F.A., Davies, T.J., Evans, E.P., Mack, D.L., Gardner, R.L., and McKay, R.D. (2007). New cell lines from mouse epiblast share defining features with human embryonic stem cells. Nature *448*, 196–199.

Theunissen, T.W., Powell, B.E., Wang, H., Mitalipova, M., Faddah, D.A., Reddy, J., Fan, Z.P., Maetzel, D., Ganz, K., Shi, L., et al. (2014). Systematic identification of culture conditions for induction and maintenance of naive human pluripotency. Cell Stem Cell *15*, 471–487.

Thomson, J.A., Kalishman, J., and Hearn, J.P. (1994). Nonsurgical uterine stage preimplantation embryo collection from the common marmoset. J. Med. Primatol. *23*, 333–336.

Thomson, J.A., Kalishman, J., Golos, T.G., Durning, M., Harris, C.P., and Hearn, J.P. (1996). Pluripotent cell lines derived from common marmoset (*Callithrix jacchus*) blastocysts. Biol. Reprod. *55*, 254–259.

Thomson, J.A., Itskovitz-Eldor, J., Shapiro, S.S., Waknitz, M.A., Swiergiel, J.J., Marshall, V.S., and Jones, J.M. (1998). Embryonic stem cell lines derived from human blastocysts. Science *282*, 1145–1147.

Turksen, K., and Troy, T.C. (2001). Claudin-6: a novel tight junction molecule is developmentally regulated in mouse embryonic epithelium. Dev. Dyn. *222*, 292–300.

Van der Jeught, M., Heindryckx, B., O'Leary, T., Duggal, G., Ghimire, S., Lierman, S., Van Roy, N., Chuva de Sousa Lopes, S.M., Deroo, T., Deforce,

D., and De Sutter, P. (2014). Treatment of human embryos with the TGFβ inhibitor SB431542 increases epiblast proliferation and permits successful human embryonic stem cell derivation. Hum. Reprod. *29*, 41–48.

Weitlauf, H.M., and Greenwald, G.S. (1968). Survival of blastocysts in the uteri of ovariectomized mice. J. Reprod. Fertil. *17*, 515–520.

Wray, J., Kalkan, T., Gomez-Lopez, S., Eckardt, D., Cook, A., Kemler, R., and Smith, A. (2011). Inhibition of glycogen synthase kinase-3 alleviates Tcf3 repression of the pluripotency network and increases embryonic stem cell resistance to differentiation. Nat. Cell Biol. *13*, 838–845.

Wu, T.D., and Nacu, S. (2010). Fast and SNP-tolerant detection of complex variants and splicing in short reads. Bioinformatics *26*, 873–881.

Xue, Z., Huang, K., Cai, C., Cai, L., Jiang, C.Y., Feng, Y., Liu, Z., Zeng, Q., Cheng, L., Sun, Y.E., et al. (2013). Genetic programs in human and mouse early embryos revealed by single-cell RNA sequencing. Nature *500*, 593–597.

Yamanaka, Y., Lanner, F., and Rossant, J. (2010). FGF signal-dependent segregation of primitive endoderm and epiblast in the mouse blastocyst. Development *137*, 715–724.

Yan, L., Yang, M., Guo, H., Yang, L., Wu, J., Li, R., Liu, P., Lian, Y., Zheng, X., Yan, J., et al. (2013). Single-cell RNA-seq profiling of human preimplantation embryos and embryonic stem cells. Nat. Struct. Mol. Biol. *20*, 1131–1139.

Ye, S., Li, P., Tong, C., and Ying, Q.L. (2013). Embryonic stem cell self-renewal pathways converge on the transcription factor Tfcp2l1. EMBO J. *32*, 2548–2560.

Yi, F., Pereira, L., and Merrill, B.J. (2008). Tcf3 functions as a steady-state limiter of transcriptional programs of mouse embryonic stem cell self-renewal. Stem Cells *26*, 1951–1960.

Yi, F., Pereira, L., Hoffman, J.A., Shy, B.R., Yuen, C.M., Liu, D.R., and Merrill, B.J. (2011). Opposing effects of Tcf3 and Tcf1 control Wnt stimulation of embryonic stem cell self-renewal. Nat. Cell Biol. *13*, 762–770.

Ying, Q.L., Wray, J., Nichols, J., Batlle-Morera, L., Doble, B., Woodgett, J., Cohen, P., and Smith, A. (2008). The ground state of embryonic stem cell self-renewal. Nature *453*, 519–523.

Developmental Cell

Supplemental Information

Lineage-Specific Profiling Delineates the Emergence and Progression of Naive Pluripotency in Mammalian Embryogenesis

Thorsten Boroviak, Remco Loos, Patrick Lombard, Junko Okahara, Rüdiger Behr, Erika Sasaki, Jennifer Nichols, Austin Smith, and Paul Bertone

Supplemental Figures

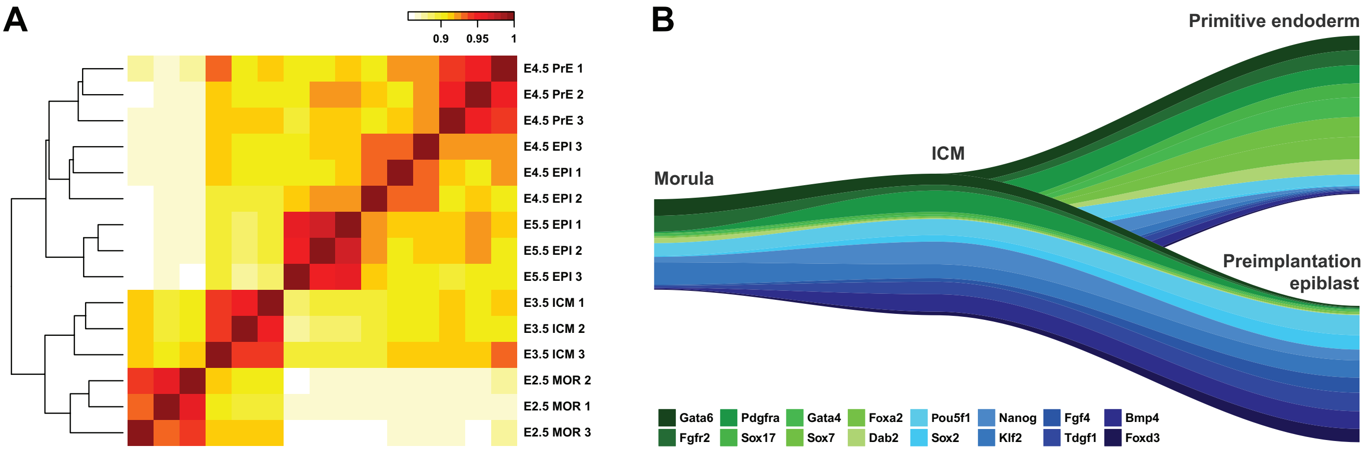

Figure S1, related to Figure 1: (A) Global correlation of mouse embryonic samples. (B) Stream plot of 16 lineage markers for PrE (green) and preimplantation epiblast (blue). Track widths represent the relative expression of the gene normalized to the mean expression across the developmental stages displayed. Genes specifically expressed in PrE or preimplantation epiblast include all established lineage markers. The early PrE markers *Gata6* and *Fgfr2* were expressed from the morula stage, with upregulation of *Pdgfra* in the early ICM. Late PrE markers, including *Gata4*, *Sox7*, *Foxa2* and *Dab2*, were specifically upregulated in PrE at E4.5, with *Gata6*, *Fgfr2* and *Pdgfra* expression maintained, consistent with the proposed sequential activation of Gata6, Sox17, Gata4 and Sox7 during PrE specification (Artus et al., 2011) and expression patterns observed by others (Chazaud et al., 2006; Gerbe et al., 2008; Guo et al., 2010; Ohnishi et al., 2014). Conversely, preimplantation epiblast cells exhibited robust expression of epiblast-specific genes, including *Sox2*, *Nanog*, *Klf2*, *Bmp4* and *Fgf4* (Guo et al., 2010; Kurimoto et al., 2006; Tang et al., 2010a).

A

MOR

Term	P-val. Adj (BH)
amine metabolic process	0.02269575
cellular biogenic amine metabolic process	0.02269575
cellular amine metabolic process	0.0292063
in utero embryonic development	0.04885411

MOR, ICM

Term	P-val. Adj (BH)
organ development	5.065E-05
multicellular organismal process	0.0004266
system development	0.0004266
regulation of cell differentiation	0.00494365
tissue development	0.00641332
regulation of multicellular organismal process	0.00641332
multicellular organismal development	0.00641332
regulation of developmental process	0.01695561
cell differentiation	0.02443758
cell development	0.02450709
anatomical structure development	0.02478382
regulation of multicellular organismal development	0.04258611
positive regulation of developmental process	0.0484731
small molecule biosynthetic process	0.04881227
response to external stimulus	0.05219916
cellular developmental process	0.05219916

MOR, ICM, pre-EPI

Term	P-val. Adj (BH)
detoxification of copper ion	0.01028629
nitric oxide mediated signal transduction	0.01537011
regulation of humoral immune response	0.01537011
response to copper ion	0.01537011
regulation of autophagy	0.02056179
regulation of acute inflammatory response	0.02176294
regulation of developmental growth	0.02176294
regulation of response to external stimulus	0.02176294
humoral immune response	0.02176294
response to external stimulus	0.02176294
developmental growth	0.02571741
negative regulation of autophagy	0.03044577
cellular zinc ion homeostasis	0.04756802
zinc ion homeostasis	0.04756802
positive regulation of developmental process	0.04909514
humoral immune response	0.04909514
response to metal ion	0.04940919
positive regulation of developmental growth	0.04940919
autophagy	0.04940919
acute inflammatory response	0.04940919
cellular response to metal ion	0.04940919
cellular divalent inorganic cation homeostasis	0.04940919
response to toxin	0.04940919
cellular response to inorganic substance	0.04940919
divalent inorganic cation homeostasis	0.04940919
cellular response to chemical stimulus	0.04940919
immunoglobulin mediated immune response	0.04940919
B cell mediated immunity	0.04940919
cell projection assembly	0.04940919
second-messenger-mediated signaling	0.04940919
positive regulation of transmembrane transport	0.04940919
growth	0.04940919
developmental cell growth	0.04940919

ICM

Term	P-val. Adj (BH)
response to chemical stimulus	0.00052285
response to wounding	0.00175971
intracellular protein kinase cascade	0.00175971
cellular response to chemical stimulus	0.00175971
cell adhesion	0.00412043
biological adhesion	0.00412043
response to stimulus	0.00461262
odontogenesis	0.00461262
cellular response to organic substance	0.00764043
regulation of multicellular organismal process	0.00865531
regulation of intracellular protein kinase cascade	0.00865531
multicellular organismal process	0.00977462
organ development	0.01234617
cell communication	0.01375272
phospholipid homeostasis	0.01375272
defense response	0.01375272
response to external stimulus	0.01458803
response to organic substance	0.01458803
inflammatory response	0.01458803
regulation of response to stress	0.01642693
cell-cell adhesion	0.01642693
gland development	0.01642693
odontogenesis of dentin-containing tooth	0.01642693
response to metal ion	0.01646228
MAPK cascade	0.01714087
cytokine production	0.01857364
system development	0.01999361

ICM, preiml. EPI

Term	P-val. Adj (BH)
response to chemical stimulus	0.00010514
multicellular organismal process	0.00013049
response to stimulus	0.00254199
cellular response to organic substance	0.00254199
response to organic substance	0.00254199
developmental process	0.00254199
anatomical structure development	0.00297988
cell migration	0.00323982
cellular response to chemical stimulus	0.00347489
cell motility	0.00585354
localization of cell	0.00585354
multicellular organismal development	0.00698981
cellular response to cytokine stimulus	0.00839442
anatomical structure morphogenesis	0.00839442
cell-cell signaling	0.00839442
immune system process	0.00839442
negative regulation of multicellular organismal process	0.00839442
secretion	0.00839442
segmentation	0.00839442
cytokine secretion	0.00839442
regulation of cell migration	0.00948099
secretion by cell	0.00948099
BMP signaling pathway	0.00991232
tissue development	0.01001977
response to nutrient	0.0102139
polysaccharide metabolic process	0.01024069
regulation of cell motility	0.01129983
cell activation	0.01129983
locomotion	0.01129983
regulation of locomotion	0.01214547
regulation of multicellular organismal process	0.01214547

ICM, pre-EPI, postEPI

Term	P-val. Adj (BH)
oxidation-reduction process	0.00796682

preimpl. EPI

Term	P-val. Adj (BH)
multicellular organismal process	0.00029974
high-density lipoprotein particle clearance	0.01591473
developmental process	0.01591473
multicellular organismal development	0.02802533
anatomical structure development	0.03692507
trophoblast cell migration	0.03692507
regulation of trophoblast cell migration	0.03692507
negative regulation of trophoblast cell migration	0.03692507
negative regulation of cell migration	0.03692507
negative regulation of cell motility	0.04204448
negative regulation of locomotion	0.04775401
negative regulation of cellular component movement	0.04871758
trophectodermal cellular morphogenesis	0.05865734
negative regulation of platelet activation	0.05865734
chylomicron remnant clearance	0.05865734
triglyceride-rich lipoprotein particle clearance	0.05865734
blood vessel development	0.05865734
system development	0.05865734
regulation of tube size	0.05865734
regulation of blood vessel size	0.05865734
embryo implantation	0.06594336

preimpl. EPI, postimpl. EPI

Term	P-val. Adj (BH)
response to chemical stimulus	0.00516425
cell proliferation	0.00516425
organ morphogenesis	0.00516425
positive regulation of cell development	0.00516425
positive regulation of MAP kinase activity	0.00516425
generation of neurons	0.00516425
neural crest cell migration	0.00516425
anatomical structure morphogenesis	0.00516425
positive regulation of neurogenesis	0.00516425
branching morphogenesis of a tube	0.00516425
cell morphogenesis involved in differentiation	0.00516425
cell death	0.00516425
organ development	0.00516425
cellular response to chemical stimulus	0.00516425
programmed cell death	0.00516425
small molecule metabolic process	0.00516425
death	0.00516425
response to superoxide	0.00516425
response to oxygen radical	0.00516425
negative regulation of cellular process	0.00589999
regulation of developmental process	0.00589999
response to organic substance	0.00589999
neurogenesis	0.00643218
apoptotic process	0.00675768

postimpl. EPI

Term	P-val. Adj (BH)
nervous system development	7.0003E-05
cell differentiation	7.0003E-05
cell communication	7.0003E-05
signaling	7.0003E-05
anatomical structure development	7.0003E-05
system development	9.567E-05
cellular developmental process	0.00015016
multicellular organismal development	0.00015016
positive regulation of biological process	0.00016447
positive regulation of cellular process	0.00016447
regulation of multicellular organismal development	0.00021907
signal transduction	0.00021907
developmental process	0.00022631
neurogenesis	0.00022631
regulation of response to stimulus	0.00032091
multicellular organismal process	0.00032091
cellular response to stimulus	0.00043936
response to stimulus	0.00063096
neuron differentiation	0.00078691
cell surface receptor signaling pathway	0.00079899
regulation of cell differentiation	0.00095642
generation of neurons	0.0016368
regulation of signaling	0.00197571
regulation of nervous system development	0.00253872

B

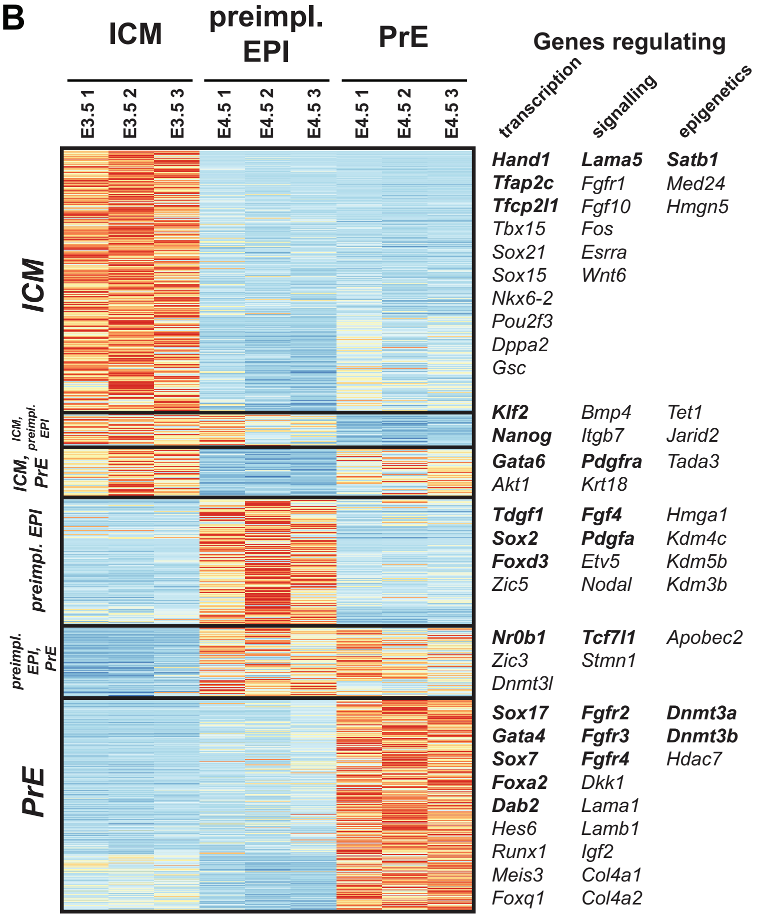

Figure S2, related to Figure 2: (A) GO enrichment for stage-specific expression in early mouse development. (B) Dynamically expressed genes during epiblast and PrE specification. Selected transcription factors, signaling pathway components and epigenetic regulators are shown to the right, with a subset of lineage markers in bold.

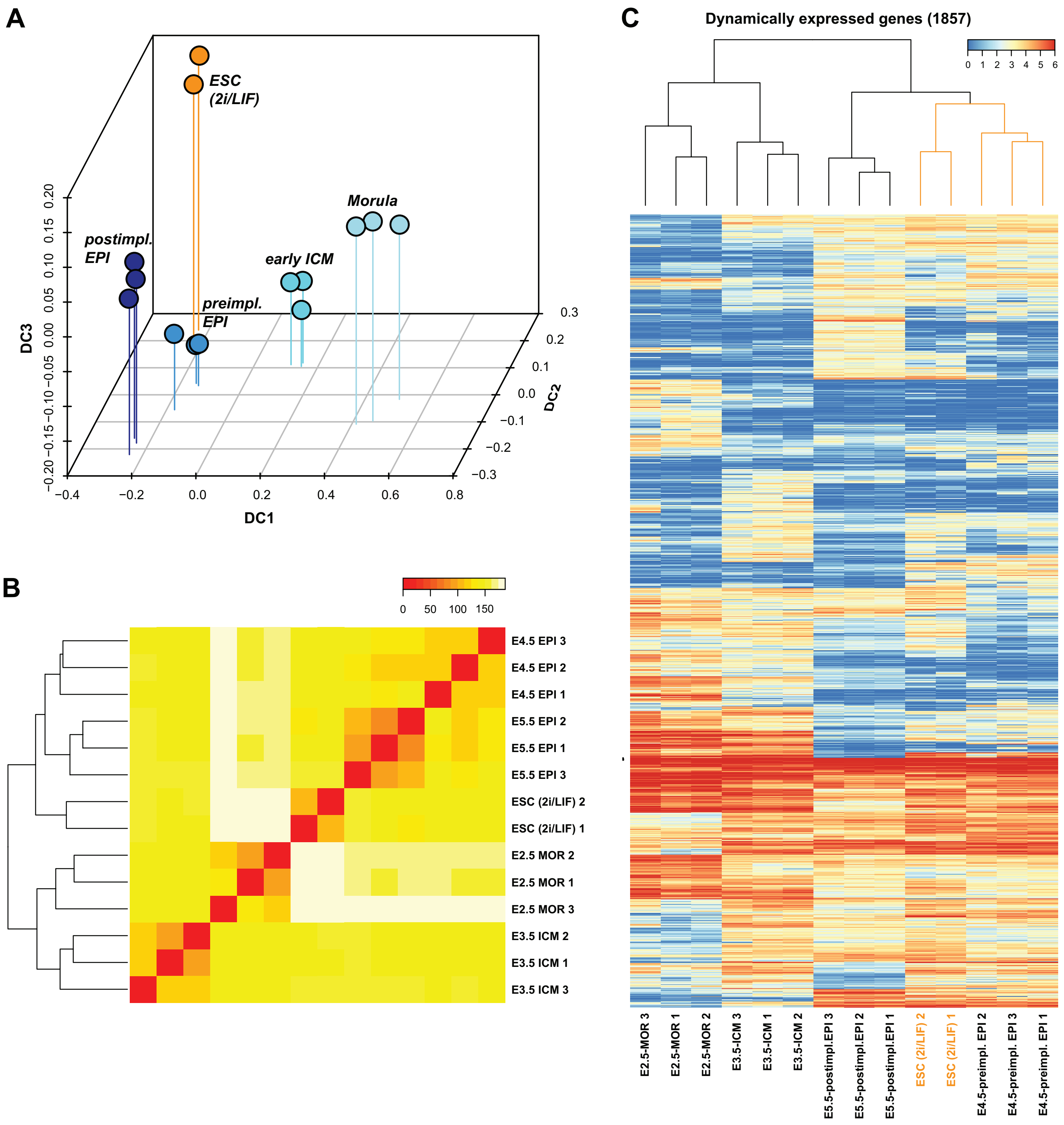

Figure S3, related to Figure 3: (A) Diffusion map of mouse embryonic samples and ESC cultured in 2i/LIF, based on the full transcriptome; DC = diffusion coefficient. (B) Global gene expression and hierarchical clustering of mouse embryonic samples and ESC in 2i/LIF. (C) Heatmap and hierarchical clustering based on the expression of dynamically regulated genes. The subgroup shared by ESC and E4.5 epiblast is marked in orange.

A

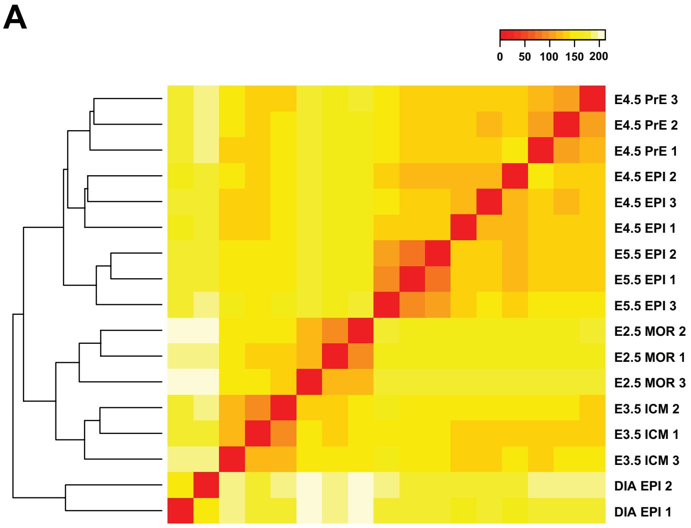

B

ESC(2i/LIF) / preimpl. EPI

DOWNREGULATED

GO enrichment					
Rank	Term	Count	ExpCount	Size	P-val.Adj (BH)
1.	cellular metabolic process	191	109	7688	4.4926E-17
2.	primary metabolic process	193	111	7865	4.9243E-17
3.	metabolic process	214	133	9440	4.8979E-16
4.	cellular process	255	188	13292	1.6293E-11
5.	cellular protein metabolic process	92	41	2908	2.0459E-11
6.	protein metabolic process	101	51	3574	6.8434E-10
7.	catabolic process	66	26	1845	6.8434E-10
8.	cellular macromolecule metabolic process	137	79	5619	6.8434E-10
9.	macromolecule metabolic process	149	91	6434	1.9745E-09
10.	cellular component organization or biogenesis	99	51	3578	2.905E-09
11.	cellular component organization	94	49	3447	2.4584E-08
12.	translation	32	8	598	3.0756E-08
13.	cellular catabolic process	55	22	1584	1.1601E-07
14.	cellular component biogenesis	50	21	1451	9.455E-07
15.	small molecule metabolic process	60	27	1937	1.1433E-06
16.	cell cycle	42	16	1132	2.0568E-06
17.	cellular component organization or biogenesis at cellular level	77	40	2851	2.1182E-06
18.	biosynthetic process	101	60	4235	3.8393E-06
19.	macromolecular complex subunit organization	35	13	944	3.1918E-05
20.	cellular biosynthetic process	95	58	4093	3.9861E-05
21.	organophosphate metabolic process	40	17	1183	4.1803E-05
22.	cellular component organization at cellular level	70	39	2731	6.6093E-05
23.	cellular component assembly	42	19	1312	8.0252E-05
24.	cell cycle process	30	11	776	8.2357E-05
25.	heterocycle metabolic process	37	16	1127	0.00020108

UPREGULATED

GO enrichment					
Rank	Term	Count	ExpCount	Size	P-val.Adj (BH)
1.	cellular biosynthetic process	31	12	4093	0.00028531
2.	biosynthetic process	31	13	4235	0.00028531
3.	cellular process	60	40	13292	0.00028531
4.	melanosome organization	3	0	11	0.0017611
5.	pigment granule organization	3	0	12	0.00187446
6.	positive regulation of aldosterone metabolic process	2	0	2	0.0021249
7.	positive regulation of aldosterone biosynthetic process	2	0	2	0.0021249
8.	cellular metabolic process	41	23	7688	0.00239135
9.	establishment of localization	24	10	3277	0.00317487
10.	regulation of cellular biosynthetic process	22	9	2903	0.0037436
11.	negative regulation of cellular biosynthetic process	12	3	959	0.0037436
12.	cellular pigmentation	3	0	20	0.0037436
13.	negative regulation of biosynthetic process	12	3	976	0.0037436
14.	regulation of biosynthetic process	22	9	2938	0.0037436
15.	cellular nitrogen compound metabolic process	29	14	4674	0.00391938
16.	positive regulation of transcription, DNA-dependent	12	3	998	0.00391938
17.	cellular component organization	24	10	3447	0.00391938
18.	nitrogen compound metabolic process	29	14	4716	0.00422368
19.	positive regulation of RNA metabolic process	12	3	1020	0.00422368
20.	nucleobase-containing compound metabolic process	28	13	4490	0.00433432
21.	negative regulation of cellular macromolecule biosynthetic process	11	3	893	0.00525252
22.	cellular component organization or biogenesis	24	11	3578	0.00525252
23.	cellular macromolecule biosynthetic process	23	10	3345	0.00525252
24.	negative regulation of nucleobase-containing compound metabolic p	11	3	903	0.00525252
25.	negative regulation of nitrogen compound metabolic process	11	3	916	0.00566842

C

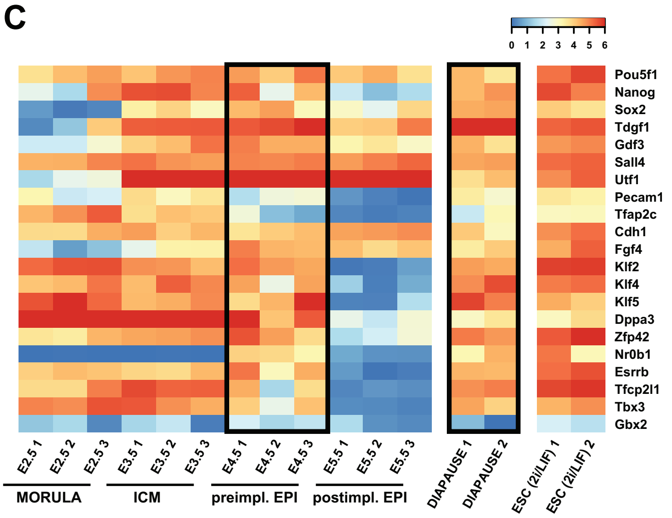

Figure S4, related to Figure 4: (A) Global expression and hierarchical clustering of mouse embryonic samples, ESC cultured in 2i/LIF and diapaused preimplantation epiblast. (B) GO term enrichment analysis based on up- and downregulated genes in normal preimplantation versus diapaused epiblast. (C) Expression of pluripotency markers in mouse embryonic samples, ESC in 2i/LIF and diapaused epiblast.

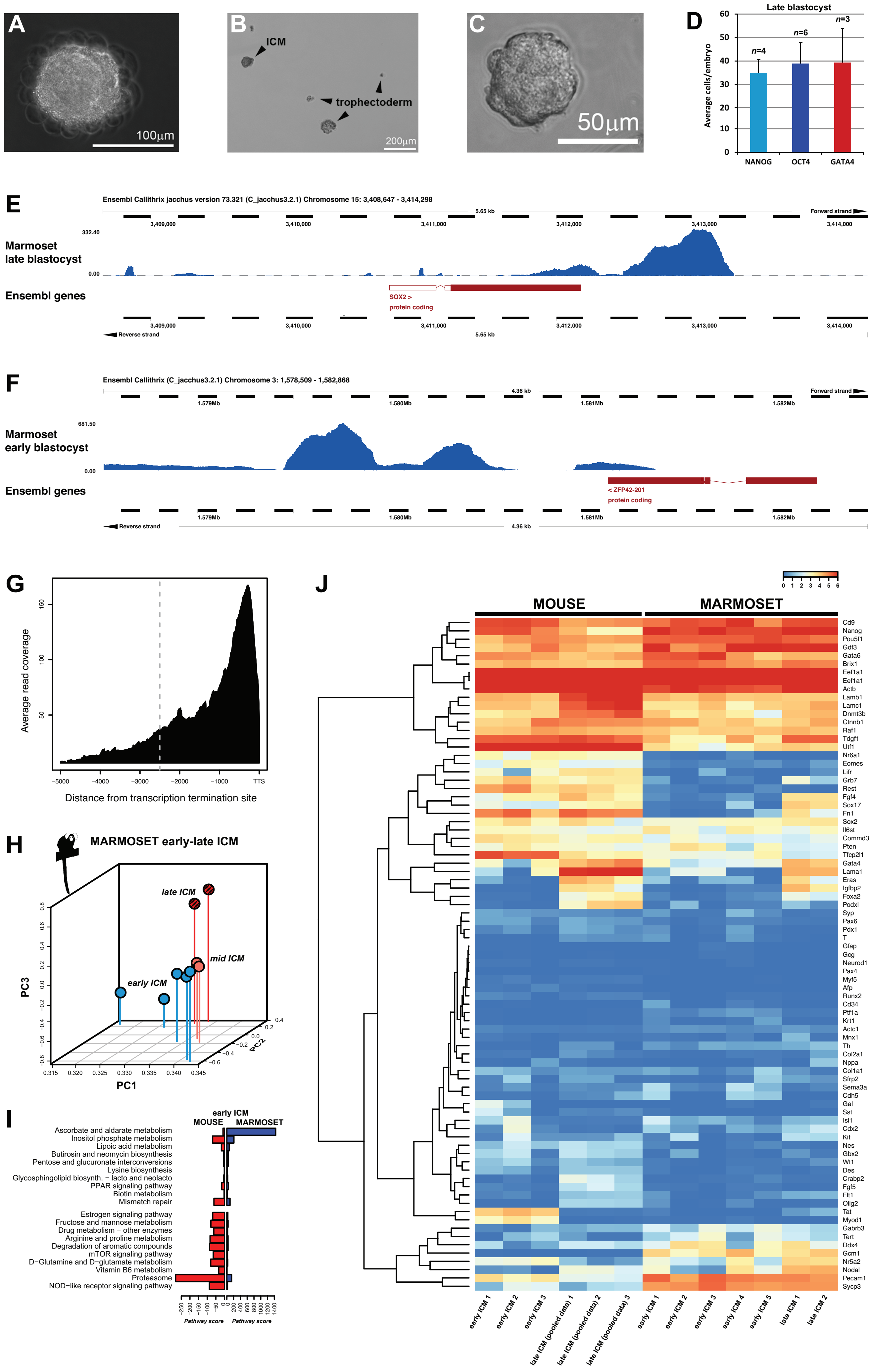
A
100μm
B
ICM
trophectoderm
200μm
C
50μm
D
Late blastocyst
Average cells/embryo
n=4
n=6
n=3
NANOG
OCT4
GATA4
E
Ensembl Callithrix jacchus version 73.321 (C_jacchus3.2.1) Chromosome 15: 3,408,647 - 3,414,298
5.65 kb
Forward strand
3,409,000
3,410,000
3,411,000
3,412,000
3,413,000
3,414,000
332.40
Marmoset late blastocyst
0.00
Ensembl genes
SOX2 >
protein coding
Reverse strand
F
Ensembl Callithrix (C_jacchus3.2.1) Chromosome 3: 1,578,509 - 1,582,868
4.36 kb
Forward strand
1.579Mb
1.580Mb
1.581Mb
1.582Mb
681.50
Marmoset early blastocyst
0.00
Ensembl genes
< ZFP42-201
protein coding
Reverse strand
G
Average read coverage
Distance from transcription termination site
TTS
H
MARMOSET early-late ICM
late ICM
mid ICM
early ICM
PC1
PC2
PC3
I
early ICM
MOUSE
MARMOSET
Ascorbate and aldarate metabolism
Inositol phosphate metabolism
Lipoic acid metabolism
Butirosin and neomycin biosynthesis
Pentose and glucuronate interconversions
Lysine biosynthesis
Glycosphingolipid biosynth. − lacto and neolacto
PPAR signaling pathway
Biotin metabolism
Mismatch repair
Estrogen signaling pathway
Fructose and mannose metabolism
Drug metabolism − other enzymes
Arginine and proline metabolism
Degradation of aromatic compounds
mTOR signaling pathway
D−Glutamine and D−glutamate metabolism
Vitamin B6 metabolism
Proteasome
NOD−like receptor signaling pathway
Pathway score
Pathway score
J
MOUSE
MARMOSET
Cd9
Nanog
Pou5f1
Gdf3
Gata6
Brix1
Eef1a1
Eef1a1
Actb
Lamb1
Lamc1
Dnmt3b
Ctnnb1
Raf1
Tdgf1
Utf1
Nr6a1
Eomes
Lifr
Grb7
Rest
Fgf4
Sox17
Fn1
Sox2
Il6st
Commd3
Pten
Tfcp2l1
Gata4
Lama1
Eras
Igfbp2
Foxa2
Podxl
Syp
Pax6
Pdx1
T
Gfap
Gcg
Neurod1
Pax4
Myf5
Afp
Runx2
Cd34
Ptf1a
Krt1
Actc1
Mnx1
Th
Col2a1
Nppa
Col1a1
Sfrp2
Sema3a
Cdh5
Gal
Sst
Isl1
Cdx2
Kit
Nes
Gbx2
Wt1
Des
Crabp2
Fgf5
Flt1
Olig2
Tat
Myod1
Gabrb3
Tert
Ddx4
Gcm1
Nr5a2
Nodal
Pecam1
Sycp3
early ICM 1
early ICM 2
early ICM 3
late ICM (pooled data) 1
late ICM (pooled data) 2
late ICM (pooled data) 3
early ICM 1
early ICM 2
early ICM 3
early ICM 4
early ICM 5
late ICM 1
late ICM 2

Figure S5, related to Figure 5: (A–C) Immunosurgery on marmoset blastocysts. Phase contrast images of (A) marmoset blastocyst after complement reaction, (B) roughly separated ICM and trophectoderm and (C) cleanly separated ICM after dissociation with a fine Pasteur pipette. (D) Manual quantification of NANOG, OCT4 and GATA4-positive cells from whole-mount immunostainings of in vivo derived, late marmoset blastocysts; *n* = number of embryos. (E) *SOX2* locus in the marmoset reference genome. Gene boundaries are annotated incorrectly with respect to transcribed sequence determined by RNA-seq. (F) A minority of genes such as *ZFP42* (*REX1*) were not accurately quantified due to pronounced discrepancies between marmoset gene loci and sequencing data that remained unreconciled by automated reannotation. (G) Read coverage for genes >3 kb illustrating 3′ bias and threshold implemented to restrict transcript length (dashed line). (H) Principal component analysis of marmoset embryonic samples. (I) Pathway expression scores for the 10 most up- and downregulated signaling pathways (KEGG). Scores were calculated by summing FPKM values of individual pathway components followed by normalization for pathway size. (J) Expression of pluripotency and lineage markers in mouse and marmoset early and late ICM samples. The mouse late ICM sample was generated by *in silico* pooling of RNA-seq reads from individual E4.5 EPI and E4.5 PrE samples at a ratio of 1:1. This reflects the ratio of epiblast to PrE cells in the late marmoset ICM (D) to allow comparison of late ICM in both species.

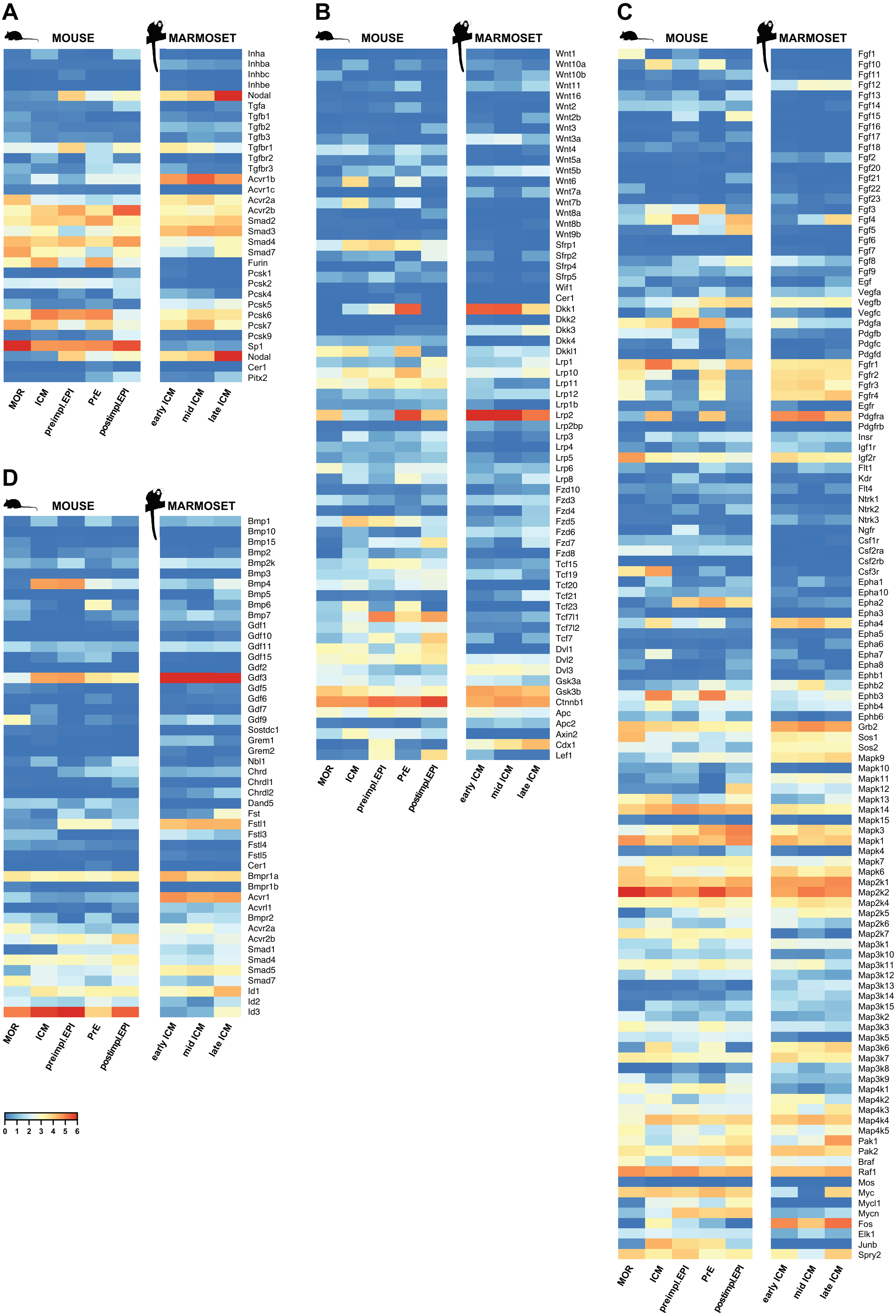

Figure S6, related to Figure 6: Expression data for (A) TGFß/NODAL, (B) WNT, (C) FGF and (D) BMP pathways.

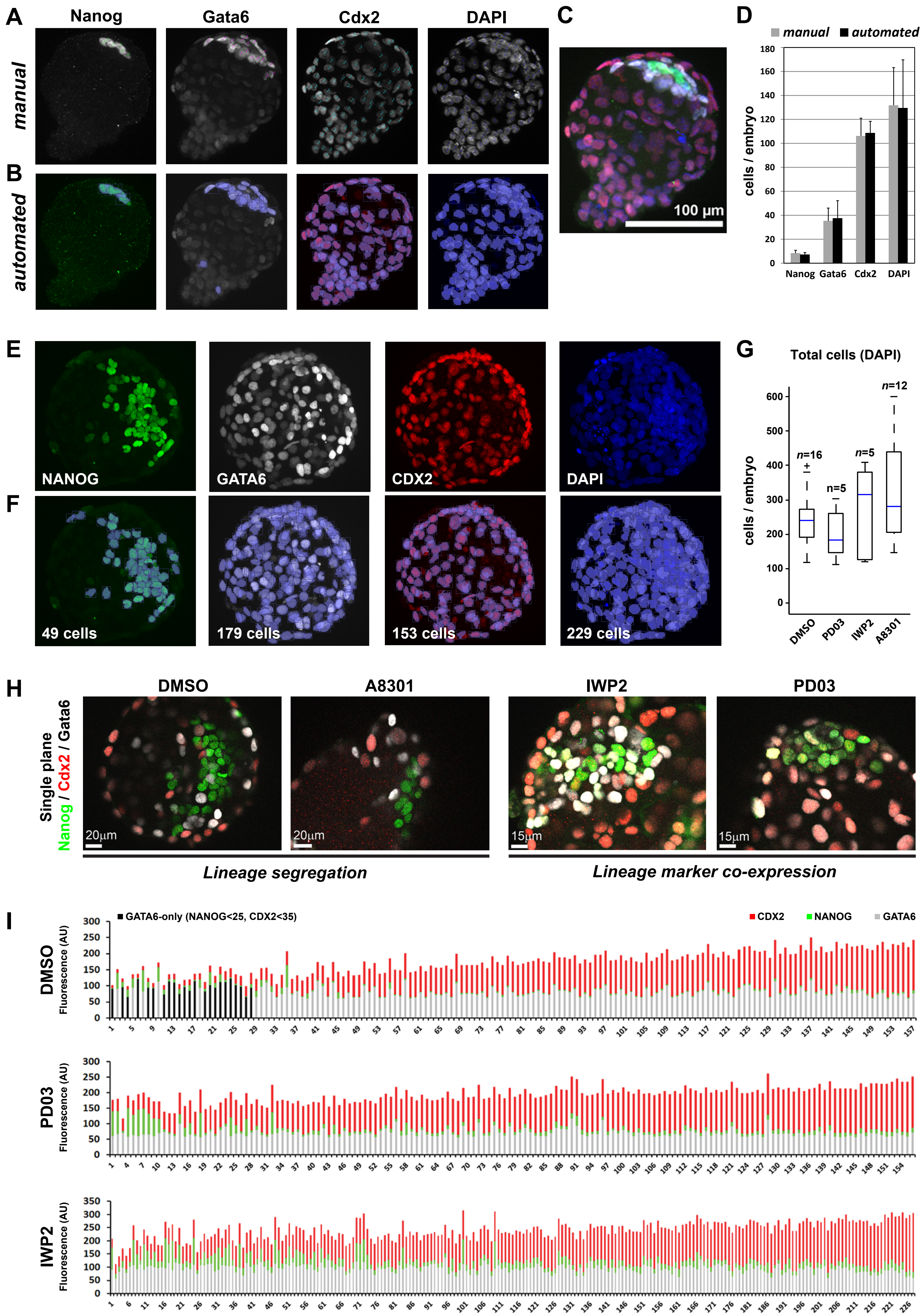

A
B
C
D
E
F
G
H
I
Nanog
Gata6
Cdx2
DAPI
manual
automated
100 µm
manual
automated
cells / embryo
Nanog
Gata6
Cdx2
DAPI
NANOG
GATA6
CDX2
DAPI
49 cells
179 cells
153 cells
229 cells
Total cells (DAPI)
n=16
n=5
n=5
n=12
DMSO
PD03
IWP2
A8301
Single plane
Nanog / Cdx2 / Gata6
DMSO
A8301
IWP2
PD03
20µm
15µm
Lineage segregation
Lineage marker co-expression
GATA6-only (NANOG<25, CDX2<35)
CDX2
NANOG
GATA6
Fluorescence (AU)
DMSO
PD03
IWP2

Figure S7, related to Figure 6: (A–D) Comparison of manual and automated analysis of whole-mount embryo immunofluorescence confocal images. Late mouse blastocysts were stained for Nanog (epiblast), Gata6 (PrE), Cdx2 (trophectoderm) and DAPI (total cell number) and quantified by (B) manual counting of confocal *Z*-projections and (C) automated object quantification using Volocity. (D) Manual versus automated quantification; error bars = standard deviation. (E) Confocal *Z*-projection of a DMSO-treated (control) marmoset embryo stained for NANOG, GATA6, CDX2 and DAPI by immunocytochemistry. (F) Automated object quantification for the embryo shown in (E). The total number of cells identified is indicated in the lower left for each channel. (G) Quantification of total cell number (DAPI) from embryos cultured with and without inhibitors. Outliers are indicated with a cross. *P*-values were determined by one-way ANOVA with Tukey HSD testing. No significant differences were observed. (H) Single-plane confocal section of NANOG, CDX2 and GATA6 in inhibitor-treated marmoset embryos. (I) Fluorescence intensities of GATA6-positive cells in one representative embryo per experimental group. Bars represent individual cells. NANOG and CDX2 signal did not exceed background fluorescence in cells marked GATA6-only (black). For a cell to qualify as such, NANOG and CDX2 levels were no greater than background.

Table Legends

Table S1, related to Figure 1: Mouse gene expression levels for the developmental stages and samples analyzed in fragments per kilobase of exon per million fragments mapped (FPKM).

Table S2, related to Figure 2: Dynamically expressed genes in mouse embryonic lineages during development from the E2.5 morula stage to E5.5 postimplantation.

Table S3, related to Figure 2: Constitutively expressed genes in mouse development from morula to postimplantation stages.

Table S4, related to Figures 2 and 4: Mouse gene expression modules for the developmental stages and samples analyzed. Modules were derived by hierarchical clustering of scaled expression values.

Table S5, related to Figure 2: Genes differentially expressed between pairs of conditions in mouse embryonic development, diapaused epiblast and cultured ESC.

Table S6, related to Figure 5: Marmoset gene expression levels over the developmental stages analyzed in fragments per kilobase of exon per million fragments mapped (FPKM).

Table S7, related to Figure 5: Comparison of orthologous genes in mouse and marmoset ICM. Embryo-matched lineage-specific data from mouse epiblast and PrE samples were merged for compatibility with marmoset ICM.

Extended Experimental Procedures

Mouse strains

Embryos were generated by intercrosses between *Pdgfra::GFP* (Hamilton et al., 2003) and strain 129 estrus-checked females. Experiments were performed in accordance with EU guidelines for the care and use of laboratory animals, and under authority of UK governmental legislation. Use of animals in this project was approved by the ethical review committee for the University of Cambridge, and relevant Home Office licences are in place.

Mouse embryo staging and sample acquisition

Staging of mouse embryos was based on the assumption that, on average, mating occurred at midnight so that at midday embryos were assigned E0.5. Embryos were collected at the relevant stages from the oviduct or uterus in M2 medium (Sigma). Embryos were carefully matched by morphology and stage of development. An important aspect of this study was to profile freshly harvested, *in vivo* developed embryos. Consequently we avoided embryo culture and processed embryos as quickly as possible.

At the 8-cell stage embryos were flushed from the oviducts at 11.00am (just before E2.5). Embryos selected for precompaction with clearly separable eight individual cells. Zona pellucidae were removed with acidic Tyrode's solution (Sigma), cells were dissociated by brief exposure (<5min) to a 1:1 mixture of 0.025% trypsin plus EDTA (Invitrogen) and 0.025% trypsin (Invitrogen) plus 1% chick serum (Sigma). All individual samples (E2.5 MOR 1–3) contain 8 cells from one embryo.

Early blastocyst embryos represent the 32-cell stage harvested at 10.00am (prior to E3.5). For all early ICM samples blastocoel expansion was less than 50% of the total volume. Zona pellucidae were removed with acidic Tyrode's solution (Sigma), ICM were isolated by immunosurgery (Solter and Knowles, 1975, see below) and any residual trophectoderm was thoroughly removed by repetitive manual pipetting. ICM were dissociated by exposure (5–10min) to a 1:1 mixture of 0.025% trypsin plus EDTA (Invitrogen) and 0.025% trypsin (Invitrogen) plus 1% chick serum (Sigma). All cells were dissociated into singletons. Individual cell numbers of 12, 14 and 16 were processed immediately for RNA-seq.

Late blastocyst embryos were collected at E4.5 (11.00am). EPI and PrE have fully segregated at this stage (Plusa et al., 2008, Grabarak et al., 2012) and PrE cells can be visualized with the Pdgfra:GFP reporter which we used as an additional criterion to classify late blastocysts. Immunosurgery and single cell dissociation were carried out as described for early ICM with a slightly longer incubation time in dissociation buffer of 10–15min. Pulled glass capillaries of decreasing inner diameter were used to gently dissociate the ICM into single cells. We obtained 40–50 cells per embryo of which 10–20 remained in duplets or triplets. The vast majority of cells were either negative or strongly positive for Pdgfra-GFP. Single cells were sorted manually under a fluorescence dissection microscope based on GFP expression. Individual samples (e.g., E4.5 EPI) contained 10–20 single cells.

Postimplantation epiblast samples were isolated by manual dissection between 12.00–3.00 pm (just after E5.5). Embryos used had developed a cavity and the epiblast had formed an epithelium. Extraembryonic ectoderm was first removed with a wide mouth-controlled pipette, and the visceral endoderm was mechanically separated by drawing the embryo portion through a second pipette with a narrower aperture. Postimplantation epiblast samples were confirmed to be free of GFP-positive visceral endoderm. Epiblast dissociation was carried out as described for early ICM with a slightly longer incubation time of 10–15min. For individual samples (e.g., E5.5 EPI), 20 single cells were randomly selected and processed for RNA-seq.

Diapause induction

Embryonic diapause was induced by surgical removal of both ovaries from female mice on the morning of the third day of pregnancy (E2.5) without subsequent progesterone treatment. Diapaused embryos were flushed 4 days later and the epiblast was isolated in the same manner as for E4.5 embryos (see below).

Marmoset embryo collection

Marmoset blastocysts were staged based on embryonic day, diameter and blastocoel formation. Zona pellucidae were removed using acidic Tyrode's solution (Sigma) and embryos were subjected to immunosurgery as described below, using a custom rabbit polyclonal anti-marmoset antibody. Dissociation of marmoset ICM was carried out as for mouse embryos at the late blastocyst stage. Animals were obtained from self-sustaining colonies and housed according to standard husbandry guidelines. Protocols and institutional regulations for the care and experimental use of marmosets were strictly followed. Experiments at the DPZ were conducted under licence number AZ 33.42502–066/06. Experiments using marmosets at the CIEA were approved by the animal research committee (CIEA: 11028) and performed in compliance with guidelines set forth by the Science Council of Japan.

Immunosurgery

Mouse (E3.25 and E4.5) and marmoset blastocysts were processed by immunosurgery based on previously described procedures (Nichols et al., 1998; Solter and Knowles, 1975). Embryos were incubated for 15min in a 1:5 dilution of anti-mouse (Sigma) or anti-marmoset (in-house) rabbit serum in N2B27 medium, washed in N2B27 and incubated a further 15min in a 1:5 dilution of rat serum for the complement reaction. Embryos were transferred to N2B27 and incubated a further 15min for efficient cell lysis. ICM were dissociated from residual trophectoderm with a pulled Pasteur pipette of internal diameter just wider than the ICM.

Single cell dissociation

Dissociation of morula, ICM and postimplantation epiblast cells was performed in a 1:1 mixture of 0.025% trypsin plus EDTA (Invitrogen) and 0.025% trypsin (Invitrogen) plus 1% chick serum (Sigma). Morulae and early ICM were incubated for 5–10min at 37°C, and E4.5 mouse ICM and postimplantation epiblasts were incubated for 10–15min. Explants were subsequently washed in N2B27 supplemented with 10µM HEPES and 1.5mg/ml BSA (Gibco) and dissociated in a small drop of medium using blunted microcapillaries pulled to an inner diameter large enough to accommodate approximately 2–3 cells.

Embryonic stem cell culture

E14TG2a cells derived from mouse strain 129/Ola (Hooper et al., 1987) were used as a reference ESC line and cultured in 2i/LIF medium on gelatin (Ying et al., 2008). N2B27 (1:1 DMEM/F-12 and Neurobasal media, N2 (in-house) and B27 (Gibco) additives, 2mM L-glutamine and 100µM ß-mercaptoethanol) was supplemented with 1µM PD0325901, 3µM CHIR99021 and 10ng/ml LIF (in-house). Cells were dissociated by conventional methods in bulk culture. Single-cell suspensions were prepared and 20 cells were manually selected with a blunt microcapillary.

Immunofluorescence and cell counts

Embryos were stained as previously described (Nichols et al., 2009). Primary antibodies used were: Nanog (ReproCell RCAB0002P-F (1:300 dilution) or Cell Signaling Technology 4893 (1:400)), Gata4 (Santa Cruz sc1237 (1:100)), Oct4 (Santa Cruz sc8628 (1:300)), Gata6 (R&D AF1700 1:100)), Cdx2 (Biogenex MU392A 1:200)), Klf4 (Santa Cruz sc20691 (1:400)), Tfcp2l1 (R&D AF5726 (1:400)), Sox17 (R&D AF1924 (1:100)) and E-cadherin (Abcam ab15148 (1:200)). Cell counts from individual embryos were used for one-way ANOVA with Tukey HSD testing to identify significant changes between any two means (Figures 6C and 6D).

Sample processing and RNA-seq library construction

cDNA synthesis from small numbers of cells was carried out using whole-transcriptome preamplification (Tang et al., 2010b; Tang et al., 2009). Typically 10–20 cells (8 for morulae) were transferred immediately into 4.5μl single-cell lysis buffer under a dissection microscope and snap frozen on dry ice. cDNA was synthesized and amplified for 20 cycles using a single-temperature protocol in a Veriti 96-well thermocycler (Applied Biosystems). cDNA samples were sheared by ultrasonication on a Covaris S2 for 80s with parameter settings: Duty Cycle 10, Cycles per Burst 200, Intensity 4. Samples were ethanol precipitated and resuspended in Tris buffer for subsequent enzymatic modifications. End repair was carried out with T4 DNA polymerase and T4 polynucleotide kinase (New England Biolabs) and samples were purified with Ampure XP paramagnetic beads (Beckman Coulter). Blunt-end, 3′-phosphorylated products were 3′-adenylated with exo^{-} Klenow fragment in the presence of dATPs (New England Biolabs), purified with Ampure beads, and ligated to sequencing adapters (Illumina and Bioo Scientific) by T4 DNA ligase at 20°C for 30 minutes. PCR amplification of library constructs was carried out with AccuPrime DNA polymerase (Invitrogen) for 13 cycles on a Bio-Rad C1000 thermocycler using denaturing and annealing conditions optimized for even A/T vs. G/C processing (Aird et al., 2011). Molarity and size distribution of sequencing libraries was assessed by DNA 1000 microfluidic chips on the 2100 Bioanalyzer (Agilent). Sequencing was performed in 100bp paired-end format on the Illumina HiSeq 2000 at high read depth (up to 250M per sample).

RNA-seq alignment and quantification

Remaining preamplification adapters were removed prior to alignment using AdapterRemoval (Lindgreen, 2012) at both 3′ and 5′ ends of reads. Sequencing reads were aligned to the GRCm38 assembly of the mouse genome (mm10) and the CalJac3.2.1 assembly of the common marmoset genome using GSNAP (Wu and Nacu, 2010), provided with splice junction annotation from Ensembl release 70 (Flicek et al., 2014). Only read pairs with unique mapping to the genome were considered further. Transcript quantification was performed with htseq-count, part of the Bioconductor *HTSeq* package (Anders et al., 2014), using gene annotation from Ensembl release 70. Samples were corrected for sequencing library size using the size factors computed by the *DESeq* package (Anders and Huber, 2010) and normalized for gene length to yield FPKM values. The cDNA preamplification protocol exhibits limited transcript processivity from the 3′ end (Tang et al., 2010a); we therefore restricted transcript lengths to 2500 bp for longer genes to mitigate this effect (Figure S5G). Global analyses, such as sample clustering and diffusion maps, were based on variance-stabilized counts computed by *DESeq*. In addition to the dataset described here, two previously published mouse E3.5 preimplantation epiblast samples produced as part of the same experiment and under identical conditions were analyzed in this study (ArrayExpress E-MTAB-2555, Boroviak et al., 2014). For comparison with published single-cell and bulk RNA-seq data, sequencing reads were obtained from the European Nucleotide Archive (Pakseresht et al., 2014) and processed as described above.

Marmoset gene reannotation

We observed that annotation of marmoset genes was often incorrect at the 3′ UTR (Figure S5E). As a result, documented 3′ end bias introduced by cDNA preamplification (Kurimoto et al., 2006, Tang et al., 2010b) led to underestimation of expression levels of affected genes. To address this, marmoset transcripts were assembled *de novo* from RNA-seq data using Trinity (Grabherr et al., 2011). Transcript contigs were then mapped to the marmoset reference genome and transcriptome sequences with BLAT (Kent, 2002). Newly-assembled transcripts that could be uniquely assigned to a known gene were incorporated into the genome annotation as a new isoform. To avoid false positives, regions of read coverage near annotated genes that could not be linked to the gene by an assembled transcript were not included in the reannotation (Figure S5F). This revised annotation was used to quantify the expression of marmoset genes and is provided with ArrayExpress record E-MTAB-2959.

Identification and clustering of dynamically regulated genes

Differential expression analysis was performed with *DESeq*. Since the expression of many genes changes over the developmental time course profiled, dispersions were estimated using fit type 'local'. Dynamically regulated genes for a set of embryonic stages were defined as genes that were 1) differentially expressed (FDR-corrected *P*-value < 0.05) between any two stages and 2) robustly transcribed (mean FPKM>10) in at least one. To identify clusters of genes that exhibit similar expression patterns, FPKM values were first scaled relative to the mean expression of the gene across all samples. Hierarchical clustering was then performed using the hclust algorithm in R. Clusters were extracted and manually classified into modules corresponding to the stage(s) of predominant expression. GO category and KEGG pathway enrichment analysis on differentially expressed and clustered genes was performed using the *GOstats* R package (Falcon and Gentleman, 2007). Genes were designated transcription factors if they were contained in a curated list of transcription factors (Vaquerizas et al., 2009) or were annotated with the GO term 'regulation of gene transcription (GO:0010468). Epigenetic regulators correspond to categories 'chromatin modification' and 'regulation of gene expression, epigenetic' (GO:0016568, GO:0040029), and signaling molecules to 'signaling' (GO:0023052).

Cross-species analysis

For comparative analyses between mouse and marmoset, orthologous genes were matched using data from Ensembl release 70 (Flicek et al., 2014). To obtain a comprehensive gene set, annotated one-to-one mouse-marmoset orthologs were supplemented with one-to-one marmoset-human orthologs corresponding to the mouse gene symbol. To render marmoset ICM samples comparable to the mouse data, EPI and PrE expression profiles from the same mouse embryos were pooled *in silico* at the observed marmoset ratio of 1:1 (Figure S5D). Expression data from mouse and marmoset orthologs are provided in Table S7.

Pathway analysis

Differential pathway analysis was performed for all 229 curated KEGG pathways. High-level comparative analyses between embryonic stages and species were based on pathway scores, defined as the sum of expression values of all components divided by the number comprising each pathway. For more detailed analyses, expression values were mapped onto pathway nodes centered around the mean expression of all genes (FPKM=24), with FPKM=0 and FPKM=100 as extremes, using PathVisio (Kutmon et al., 2015). For regulatory network analysis we produced a condensed version of PluriNetWork, curated based on published interactions (Som et al., 2010), by exclusively considering dynamically expressed genes. Dynamic expression of individual pathway components was determined by normalizing log-transformed expression values relative to the mean expression of the gene across all embryonic stages. A gene was deemed active at a given stage if relative expression was positive. Similarly, a network edge was considered active if both its source and target nodes were active. Cytoscape (Smoot et al., 2011) was used for network visualization.

Confocal imaging and analysis

Images were acquired with a Leica TCS SP5 confocal microscope. Optical section thickness ranged from 1–3µm. Images were processed using Leica software, Imaris, Volocity and ImageJ. Parameters for object identification were: guide size 500μm^3, separate objects 500 μm^3 and exclude objects larger than 500μm^3. Thresholds for background fluorescence (Gata6:45, Nanog:25, Cdx2:35, DAPI:20) were empirically determined to recapitulate manual cell counts in DMSO control embryos (Figures S7A–D). For two IWP2-treated embryos the threshold for Nanog was increased to 50 due to very bright signal to ensure accurate quantification of cell nuclei.

References

Aird, D., Ross, M.G., Chen, W.S., Danielsson, M., Fennell, T., Russ, C., Jaffe, D.B., Nusbaum, C., and Gnirke, A. (2011). Analyzing and minimizing PCR amplification bias in Illumina sequencing libraries. Genome Biol *12*, R18.

Anders, S., and Huber, W. (2010). Differential expression analysis for sequence count data. Genome Biol *11*, R106.

Anders, S., Pyl, P.T., and Huber, W. (2015). HTSeq—a Python framework to work with high-throughput sequencing data. Bioinformatics *31*, 166-9.

Boroviak, T., Loos, R., Bertone, P., Smith, A., and Nichols, J. (2014). The ability of inner-cell-mass cells to self-renew as embryonic stem cells is acquired following epiblast specification. Nat Cell Biol *16*, 516-528.

Chazaud, C., Yamanaka, Y., Pawson, T., and Rossant, J. (2006). Early lineage segregation between epiblast and primitive endoderm in mouse blastocysts through the Grb2-MAPK pathway. Dev Cell *10*, 615-624.

Falcon, S., and Gentleman, R. (2007). Using GOstats to test gene lists for GO term association. Bioinformatics *23*, 257-258.

Flicek, P., Amode, M.R., Barrell, D., Beal, K., Billis, K., Brent, S., Carvalho-Silva, D., Clapham, P., Coates, G., Fitzgerald, S.*, et al.* (2014). Ensembl 2014. Nucleic Acids Res *42*, D749-755.

Gerbe, F., Cox, B., Rossant, J., and Chazaud, C. (2008). Dynamic expression of Lrp2 pathway members reveals progressive epithelial differentiation of primitive endoderm in mouse blastocyst. Dev Biol *313*, 594-602.

Grabarek, J.B., Zyzyńska, K., Saiz, N., Piliszek, A., Frankenberg, S., Nichols, J., Hadjantonakis, A.K., and Plusa, B. (2012). Differential plasticity of epiblast and primitive endoderm precursors within the ICM of the early mouse embryo. Development *139*, 129-39.

Grabherr, M.G., Haas, B.J., Yassour, M., Levin, J.Z., Thompson, D.A., Amit, I., Adiconis, X., Fan, L., Raychowdhury, R., Zeng, Q.*, et al.* (2011). Full-length transcriptome assembly from RNA-Seq data without a reference genome. Nat Biotechnol *29*, 644-652.

Guo, G., Huss, M., Tong, G.Q., Wang, C., Li Sun, L., Clarke, N.D., and Robson, P. (2010). Resolution of cell fate decisions revealed by single-cell gene expression analysis from zygote to blastocyst. Dev Cell *18*, 675-685.

Hamilton, T.G., Klinghoffer, R.A., Corrin, P.D., and Soriano, P. (2003). Evolutionary divergence of platelet-derived growth factor alpha receptor signaling mechanisms. Mol Cell Biol *23*, 4013-4025.

Hooper, M., Hardy, K., Handyside, A., Hunter, S., and Monk, M. (1987). HPRT-deficient (Lesch-Nyhan) mouse embryos derived from germline colonization by cultured cells. Nature *326*, 292-295.

Kent, W.J. (2002). BLAT—the BLAST-like alignment tool. Genome Res *12*, 656-664.

Kutmon, M., van Iersel, M.P., Bohler, A., Kelder, T., Nunes, N., Pico, A.R., Evelo, C.T. (2015). PathVisio 3: an extendable pathway analysis toolbox. PLoS Comput Biol. *11*, e1004085.

Kurimoto, K., Yabuta, Y., Ohinata, Y., Ono, Y., Uno, K.D., Yamada, R.G., Ueda, H.R., and Saitou, M. (2006). An improved single-cell cDNA amplification method for efficient high-density oligonucleotide microarray analysis. Nucleic Acids Res *34*, e42.

Lindgreen, S. (2012). AdapterRemoval: easy cleaning of next-generation sequencing reads. BMC Res Notes *5*, 337.

Nichols, J., Silva, J., Roode, M., and Smith, A. (2009). Suppression of Erk signalling promotes ground state pluripotency in the mouse embryo. Development *136*, 3215-3222.

Nichols, J., Zevnik, B., Anastassiadis, K., Niwa, H., Klewe-Nebenius, D., Chambers, I., Scholer, H., and Smith, A. (1998). Formation of pluripotent stem cells in the mammalian embryo depends on the POU transcription factor Oct4. Cell *95*, 379-391.

Ohnishi, Y., Huber, W., Tsumura, A., Kang, M., Xenopoulos, P., Kurimoto, K., Oles, A.K., Arauzo-Bravo, M.J., Saitou, M., Hadjantonakis, A.K., et al. (2014). Cell-to-cell expression variability followed by signal reinforcement progressively segregates early mouse lineages. Nat Cell Biol *16*, 27-37.

Pakseresht, N., Alako, B., Amid, C., Cerdeno-Tarraga, A., Cleland, I., Gibson, R., Goodgame, N., Gur, T., Jang, M., Kay, S.*, et al.* (2014). Assembly information services in the European Nucleotide Archive. Nucleic Acids Res *42*, D38-43.

Plusa, B., Piliszek, A., Frankenberg, S., Artus, J., and Hadjantonakis, A.K. (2008). Distinct sequential cell behaviours direct primitive endoderm formation in the mouse blastocyst. Development *135*, 3081-3091.

Smoot, M.E., Ono, K., Ruscheinski, J., Wang, P.L., and Ideker, T. (2011). Cytoscape 2.8: new features for data integration and network visualization. Bioinformatics *27*, 431-432.

Solter, D., and Knowles, B.B. (1975). Immunosurgery of mouse blastocyst. Proc Natl Acad Sci USA *72*, 5099-5102.

Som, A., Harder, C., Greber, B., Siatkowski, M., Paudel, Y., Warsow, G., Cap, C., Scholer, H., and Fuellen, G. (2010). The PluriNetWork: an electronic representation of the network underlying pluripotency in mouse, and its applications. PLoS One *5*, e15165.

Tang, F., Barbacioru, C., Wang, Y., Nordman, E., Lee, C., Xu, N., Wang, X., Bodeau, J., Tuch, B.B., Siddiqui, A.*, et al.* (2009). mRNA-Seq whole-transcriptome analysis of a single cell. Nat Methods *6*, 377-382.

Tang, F., Barbacioru, C., Bao, S., Lee, C., Nordman, E., Wang, X., Lao, K., and Surani, M.A. (2010a). Tracing the derivation of embryonic stem cells from the inner cell mass by single-cell RNA-Seq analysis. Cell Stem Cell *6*, 468-478.

Tang, F., Barbacioru, C., Nordman, E., Li, B., Xu, N., Bashkirov, V.I., Lao, K., and Surani, M.A. (2010b). RNA-Seq analysis to capture the transcriptome landscape of a single cell. Nat Protoc *5*, 516-535.

Wu, T.D., Nacu, S. (2010) Fast and SNP-tolerant detection of complex variants and splicing in short reads. Bioinformatics *26*, 873-81.

Vaquerizas, J.M., Kummerfeld, S.K., Teichmann, S.A., and Luscombe, N.M. (2009). A census of human transcription factors: function, expression and evolution. Nat Rev Genet *10*, 252-263.

Ying, Q.L., Wray, J., Nichols, J., Batlle-Morera, L., Doble, B., Woodgett, J., Cohen, P., and Smith, A. (2008). The ground state of embryonic stem cell self-renewal. Nature *453*, 519-523.
